# The role of selection and evolution in changing parturition date in a red deer population

**DOI:** 10.1371/journal.pbio.3000493

**Published:** 2019-11-05

**Authors:** Timothée Bonnet, Michael B. Morrissey, Alison Morris, Sean Morris, Tim H. Clutton-Brock, Josephine M. Pemberton, Loeske E. B. Kruuk

**Affiliations:** 1 Research School of Biology, The Australian National University, Canberra, Australia; 2 School of Biology, University of St Andrews, St Andrews, United Kingdom; 3 Institute of Evolutionary Biology, School of Biological Sciences, University of Edinburgh, Edinburgh, United Kingdom; 4 Department of Zoology, University of Cambridge, Cambridge, United Kingdom; Centre National de la Recherche Scientifique, FRANCE

## Abstract

Changing environmental conditions cause changes in the distributions of phenotypic traits in natural populations. However, determining the mechanisms responsible for these changes—and, in particular, the relative contributions of phenotypic plasticity versus evolutionary responses—is difficult. To our knowledge, no study has yet reported evidence that evolutionary change underlies the most widely reported phenotypic response to climate change: the advancement of breeding times. In a wild population of red deer, average parturition date has advanced by nearly 2 weeks in 4 decades. Here, we quantify the contribution of plastic, demographic, and genetic components to this change. In particular, we quantify the role of direct phenotypic plasticity in response to increasing temperatures and the role of changes in the population structure. Importantly, we show that adaptive evolution likely played a role in the shift towards earlier parturition dates. The observed rate of evolution was consistent with a response to selection and was less likely to be due to genetic drift. Our study provides a rare example of observed rates of genetic change being consistent with theoretical predictions, although the consistency would not have been detected with a solely phenotypic analysis. It also provides, to our knowledge, the first evidence of both evolution and phenotypic plasticity contributing to advances in phenology in a changing climate.

## Abstract 2

Adaptive genetic evolution and phenotypic plasticity both contribute to a two-week advancement of birth dates earlier in spring in a deer population subject to temperature warming over four decades.

## Introduction

Climate change affects various aspects of biodiversity across the planet (e.g., [1, 2]). In particular, shifts in phenotypic distributions within populations are widely reported, for a variety of morphological, phenological, or life-history traits [2–4]. Surprisingly, however, little is still known about the relative contributions of mechanisms underlying these shifts [5]. Within a population, phenotypic distributions may change due to a change in population structure (e.g., age structure or sex ratio), due to phenotypic plasticity (within or between individuals), and due to genetic change [6–8]. The exact mixture of mechanisms driving phenotypic change will determine the future of a population facing a prolonged change in environmental conditions [9], for several reasons. First, the consequences of changing population structure are variable and may be idiosyncratic (e.g., [8, 10]). Second, phenotypic plasticity can provide an efficient way to cope with a changing environment, but its effect may be short-lived and even maladaptive [11–13]. Third, genetic evolution, when driven by natural selection, can improve population growth rate, potentially contributing to long-term population persistence [12].

In wild populations, the respective contributions of plasticity versus evolution remain unknown for the vast majority of documented phenotypic changes [14, 15] (note that by evolution we mean genetic change, here and in the rest of the manuscript). To date, most of the evidence for evolutionary responses to climate change comes from plants [16]. In contrast, despite numerous examples of phenotypic changes apparently related to climate, there have been surprisingly few examples demonstrating unambiguously that a vertebrate population is evolving in response to climate change (see discussions in [17–20]). This lack of evidence may, in part, be due to the question not being prioritized [14, 15]. However, it probably also reflects the substantial challenges inherent in testing for adaptive evolution, in terms of requirements for appropriate data and statistical methods. For wild populations in which experimental manipulations are not feasible, the most plausible means of testing for the genetic basis of phenotypic changes is to use long-term pedigree data to test for changes in “breeding values,” the estimated genetic merit of individuals as ascertained from the phenotypes of their relatives [21]. This needs to be done with care, as trends in predicted breeding values can be confounded with environmental trends unless appropriately controlled for [22], and the precision of estimates of evolutionary rates can be inflated if the correlation structure of breeding value estimates is not properly handled [23]. To our knowledge, among the studies of wild vertebrate populations that properly account for uncertainty in breeding value predictions, only 3 have found evidence of genetic change underlying phenotypic change in line with selection pressures changing with climate: plumage colouration in collared flycatchers [20], and body size in Siberian Jays [24] and snow voles [25]. However, only with more empirical studies explicitly testing for evolution will it become possible to say whether the current lack of evidence also reflects a generally slow rate of adaptation to environmental change in natural populations [26].

Climate change may have impacts on numerous aspects of an organism’s biology, but phenology (i.e., the seasonal timing of life-history events) appears to be particularly affected [3, 27–29]. Dramatic changes of phenologies in response to earlier onset of spring are particularly well documented in mid- and high-latitude passerines, where breeding times are occurring earlier in numerous populations and species [18, 30]. The study of avian systems in particular has shown that a fine-tuning of phenology to the climate is crucial in determining individual fitness. Mistiming between mean breeding date and a fitness optimum that shifts with climate may re-shape selective pressures and hence potentially reduce population growth rate [31, 32], although establishing the link between individual-level and population-level processes is challenging [33, 34]. The effects of climate change on mammalian phenology are less well documented and less clear than those of birds [29] and may be more complex because mammals’ long gestation times likely make their breeding phenology sensitive to climate across a longer timeframe [17]. Furthermore, despite the extensive evidence for phenotypic shifts in phenology, the few studies that test for a genetic basis to changes in phenology in wild populations have not found evidence of genetic changes [35–38]. One possible exception is the change of egg hatching date in winter moths [39], for which a common garden experiment suggested a contribution of genetic change.

In a population of red deer (*Cervus elaphus*, Linnaeus 1758) on the Isle of Rum, Northwest Scotland, parturition date has advanced at a rate of 4.2 days per decade since 1980, a change that has been linked to temperatures and other weather conditions in the year preceding parturition, especially around the time of conception [40, 41]. Previous studies of this population have shown that phenotypic plasticity in response to temperature and population structure explain a substantial proportion (23%) of the advance in parturition dates [41] and also that within-individual plasticity is sufficient to explain the population-level relationship between temperature and parturition date [42]. However, the documented plasticity does not explain the majority of the observed phenotypic change over time, leaving room for processes that have not been investigated as of yet. It is plausible that evolution plays a role because the observed phenotypic change is qualitatively consistent with a genetic response to selection: parturition date is heritable in this population [43] and also under selection for earlier dates [44].

In this study, we use quantitative genetic animal models [21, 45] to estimate the rate of evolution in parturition date and the contribution of plastic and demographic processes to the observed shift in phenology in the Rum red deer study population. We start by considering the response to selection that might be expected from the observed strength of selection and (narrow-sense) heritability of parturition date, based on a simple “breeder’s equation” prediction [46]. One of the most striking conclusions from the recent application of quantitative genetic theory in evolutionary ecology has been the failure of univariate “breeder’s equation” predictions to capture trait dynamics in wild populations [47, 48]. This may be for multiple reasons, foremost of which is likely to be the unrealistic assumption that only the focal trait is relevant. We therefore also consider a multivariate breeder’s equation [49] and ask how selection on offspring size and the genetic correlation between parturition date and size alters the expected evolutionary response. However, there is a second, less well-explored reason for the failure of the theory: predicted genetic responses to selection are often compared to observed rates of phenotypic change rather than of genetic change. Phenotypic changes are generally affected not only by genetic changes but also by numerous nongenetic processes and therefore may poorly reflect underlying genetic changes. As the central analysis of this work we use trends in breeding values and the secondary theorem of natural selection (STS) to estimate the rate of evolution in parturition date. We then test whether the estimated rate of evolution is compatible with the response to selection predicted by either the univariate or multivariate “breeder’s equation,” or with genetic drift. We also consider the effect of nongenetic processes contributing to phenotypic change and quantify the role of phenotypic plasticity in response to warming temperatures and of changes in population structure.

## Results

### Phenotypic change

**Fig 1:**
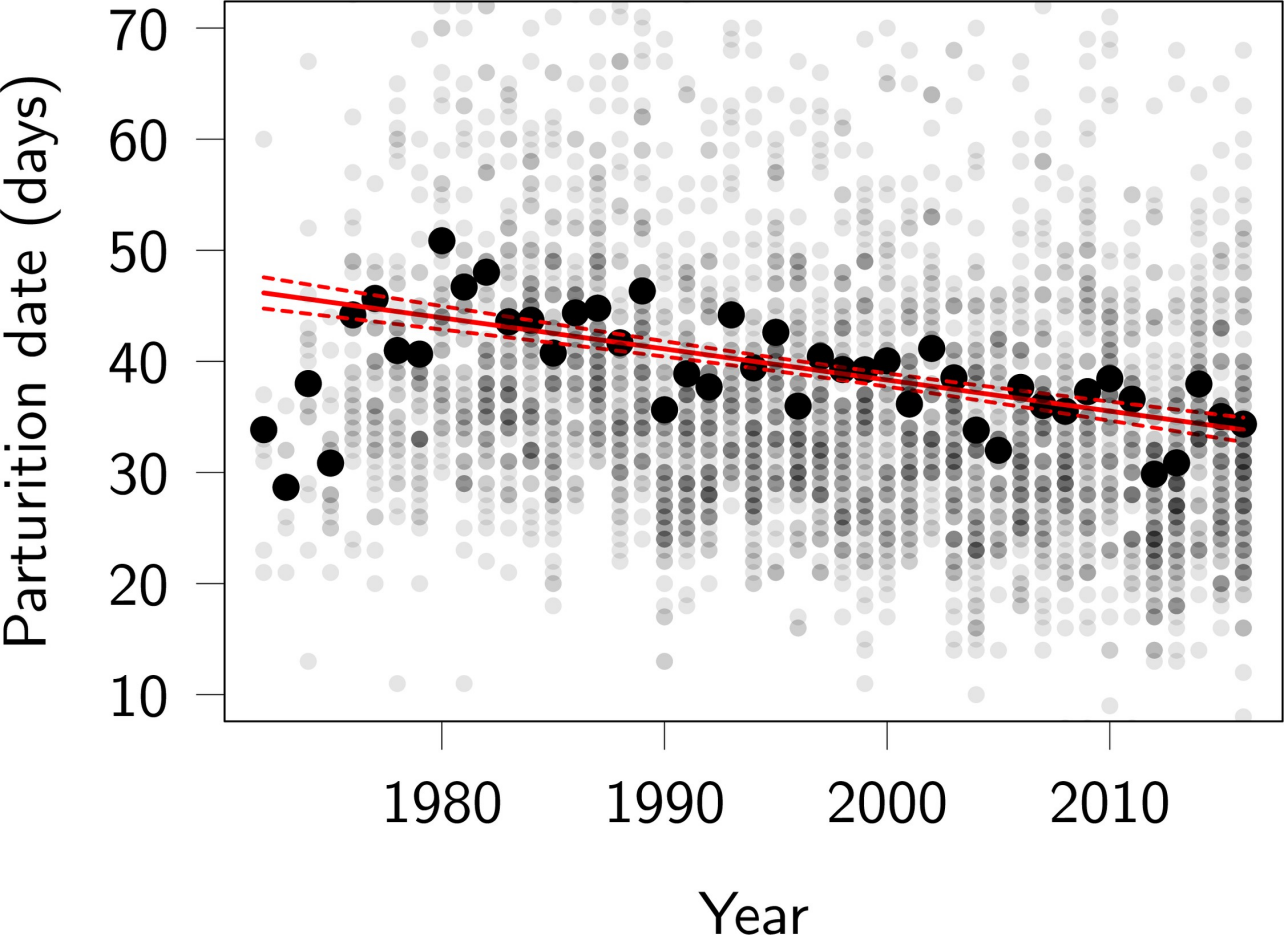
Phenotypic trend in red deer parturition dates, in days after May 1. Large black dots represent annual means, small grey dots represent individual parturition dates, with the darker shades indicating more calves being born on a given day. About 4% of individual parturition dates fall outside the plotted region (10 May–12 July; note these are still included in the analyses). The red lines represent the slope and associated 95% confidence interval of a linear regression of all individual parturition dates on year of parturition. Note that the years 1972–1975 have very negative residuals and that the rate of change over 1980–2016 is slightly underestimated by the linear regression being fitted over all years. Code for this figure is on page 5 of S1 Code.

The average parturition dates for female red deer became later from 1972 to 1980 (probably reflecting increased population density [50]), after which they advanced at an apparently constant rate (Fig 1). A linear regression estimates the change in parturition date to be a total of −12.3 (95% CI −14.5 to −10.1) days over the 45-year study period (from 1972 to 2016). The slope was identical (−12.3) when data were aggregated among females (i.e., estimated using mean parturition date over mean breeding year for each female), which may be more comparable to subsequent results.

### Sources of parturition date variation

Parturition date was influenced by a female’s reproductive status and age, but there was no clear evidence for effects of her inbreeding coefficient, of offspring sex, or of the proportion of immigrant genetic ancestry (S1 Table). Parturition date was heritable, with additive genetic variance accounting for 17% (95% CI 11% to 21%) of phenotypic variance. The individual-level repeatability of parturition date (estimated as the sum of proportions of all variance components except offspring birth year and residual) was 19%, of which additive genetic variance was most important, with permanent environment effects and maternal effects (i.e., effects related to grandmother identity) both accounting for less than 1% of total phenotypic variance in parturition date (S1 Table). The random effect for offspring birth year (which captures the variance among years over and above that corrected for the temporal linear trend) accounted for about 8% of the phenotypic variance (S1 Table). Note that proportions are essentially invariant under monotonic transformation and that these proportions of variances are equivalent on the transformed (i.e., log) and on the original data scale.

### Univariate selection and predicted response

Females with earlier parturition dates had, on average, higher lifetime breeding success (LBS): the selection differential of parturition dates estimated with LBS was −1.37 days of change within a generation (95% CI−2.43 to −0.65; see S2 Table for random effect and fixed effect estimates of the model described in Eq 2). Given the heritability of parturition date of 0.17, the univariate breeder’s equation predicts a response to selection of −0.25 days per generation (95% CI −0.55 to −0.11), that is, −1.45 days over the 45-year study period (95% CI −3.01 to −0.60). The predicted response also corresponds to −0.031 days per year (95% CI −0.068 to −0.014) or −0.019 Haldanes (95% CI −0.042 to −0.008).

Selection was weaker among females that were culled than among females that died of natural causes (S2 Fig). Considering the subset of females that died of natural causes, the univariate breeder’s equation predicts a response of −2.04 days (95% CI −3.37 to −0.95) over the study period. In contrast, using the subset of females that were culled, the univariate breeder’s equation predicts a response of 0.11 days (95% CI −0.64 to 0.93) over the study period.

### Bivariate selection and predicted response

Conditional on the fixed effects affecting each trait, the phenotypic correlation between (log) parturition date and birth weight was positive but weak (correlation = 0.12; 95% CI 0.05 to 0.16). The gradient of direct selection on (log) parturition date was negative (mode *β*_*z*_ = −0.0003; 95% CI −0.0004 to −0.0002), and that on birth weight was positive (*β*_*bw*_ = 0.0138; 95% CI 0.009 to 0.017). There was also additive genetic variance in offspring birth weight (0.68; 95% CI 0.57 to 0.90), corresponding to a heritability of 0.46 (95% CI 0.37 to 0.62). The additive genetic covariance between (log) parturition date and offspring birth weight was −1.78 (95% CI −4.38 to 0.56), corresponding to a weak negative genetic correlation of −0.16 (95% CI −0.32 to 0.05). The multivariate breeder's equation predicts a rate of evolution of −1.41 days (95% CI −2.70 to 0.11) over the study period, which is actually similar to the univariate breeder’s equation prediction of −1.45 days (difference = −0.01 days; 95% CI −0.71 to 0.55).

### Genetic contribution to phenotypic change

**Fig 2:**
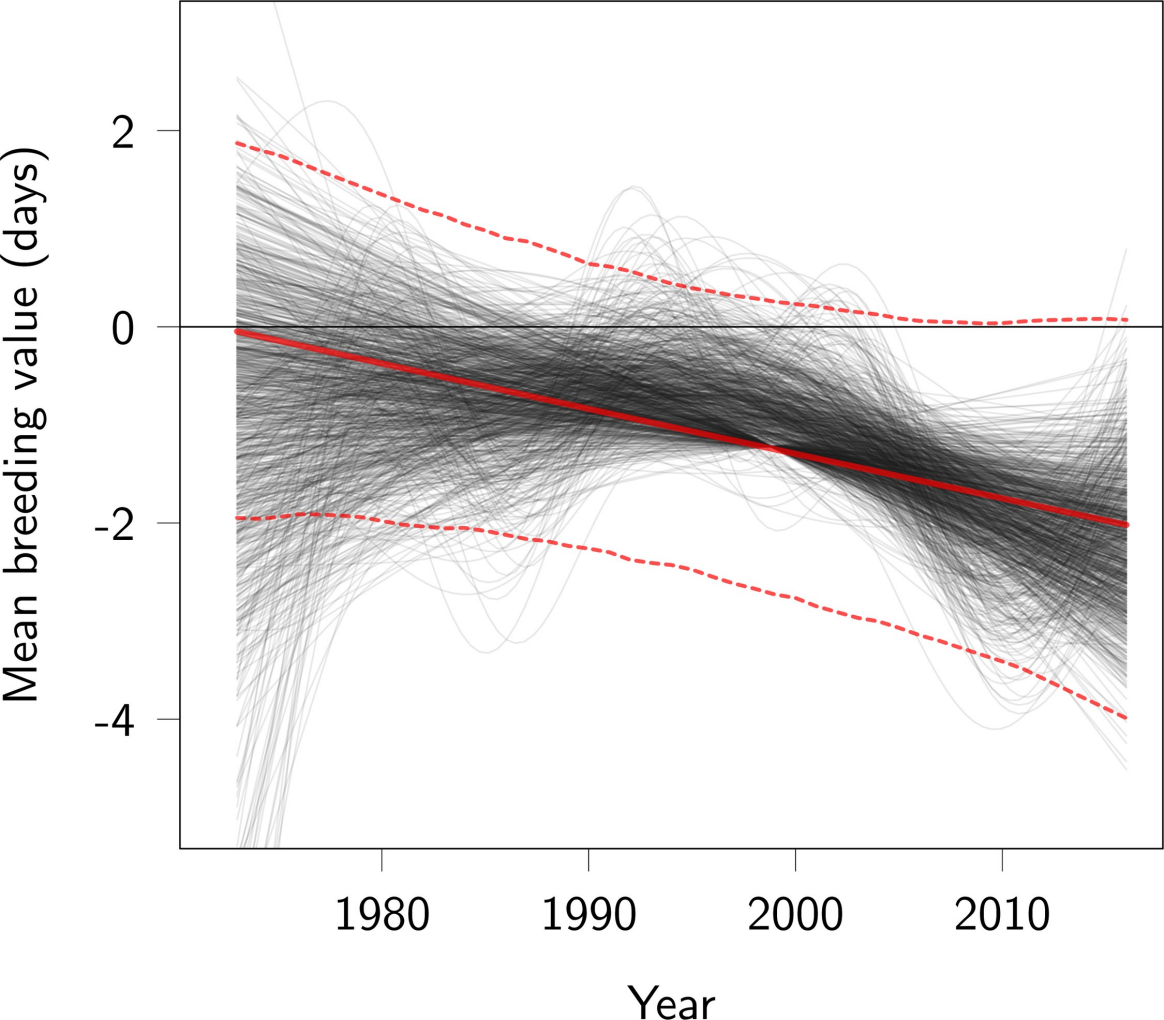
Trend in breeding values for parturition date. Red lines represent the linear regression of predicted breeding values on year (posterior mode as a thick continuous line, 95% CI as thin dotted lines). Thin black lines represent time splines of the change in predicted breeding values, allowing nonlinear change. Each black line was obtained from a different MCMC posterior sample, by fitting a spline to the mean of estimated breeding values among individuals living in the same year. Some black lines are straight because the smoother function used penalizes complex polynoms. Code for this figure is on page 18 of S1 Code. MCMC, Markov Chain Monte Carlo.

**Fig 3:**
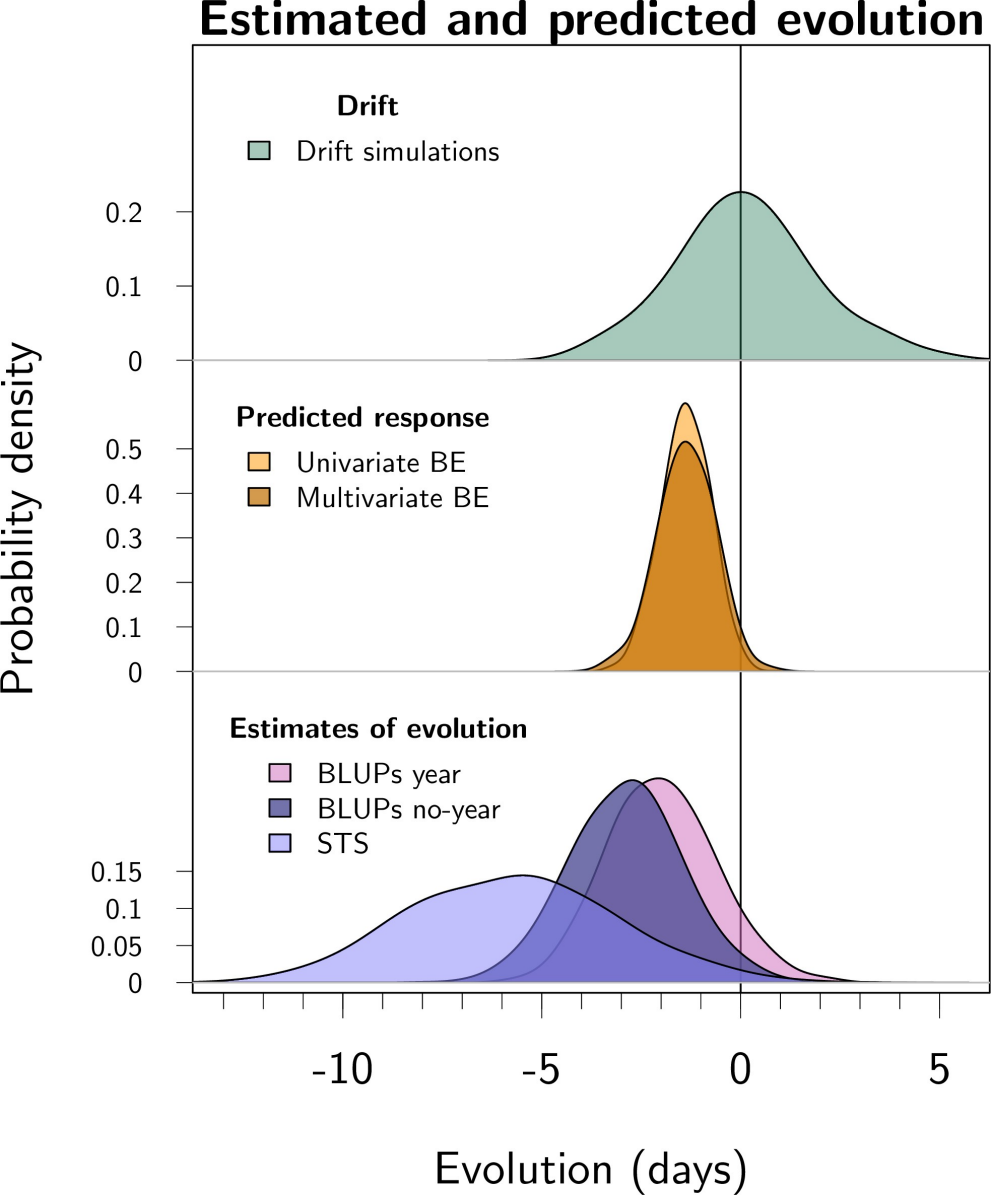
Posterior distributions for predicted and estimated evolution over the 45-year study period, from top to bottom. (1) Evolutionary change possible due to genetic drift. This distribution was generated by simulating random changes conditional on the estimated additive genetic variance and on the pedigree. (2) Predicted evolutionary response to selection from the univariate and bivariate breeder’s equations, respectively. The response to selection was estimated using univariate and bivariate breeder’s equations, where phenotypic multivariate models gave selection gradients and animal models gave additive genetic variance-covariances of parturition date and birth weight. (3) Estimated contribution of evolution estimated in 3 ways: "BLUPs year" is the conservative BLUP regression with offspring birth year fitted as a fixed effect, "BLUPs no-year" is the BLUP regression without offspring birth year fitted as a fixed effect, “STS” is the secondary theorem of natural selection using the additive genetic covariance between parturition date and fitness. Parturition date was modeled using a log-transformation, and all estimates were subsequently converted to change in days over the study period (see S1 Text). Parameter estimates are summarized in S1 Table. The distributions all have the same area, but the y-axes scales vary to help visualization. Code for this figure is on page 55 of S1 Code. BE, breeder’s equation; BLUP, best linear unbiased predictor; STS, secondary theorem of natural selection.

Using the most conservative method (namely, the model including calf birth year as a covariate), the slopes of the linear regressions of best linear unbiased predictors (BLUPs) for parturition date breeding values on mean offspring birth year—integrated over the posterior distribution—suggests an advance in breeding values, with the slope estimated at −0.10 (95% CI −0.23 to 0.03) per year on the log-transformed scale. This result is uncertain: there is a probability of 7% that the change in BLUPs is null or positive. Time splines fitted on the posterior distribution of the BLUPs visually support a linear decrease in breeding values (Fig 2). The estimated rate of evolution corresponds to a total change over the study period of −2.1 days (95% CI −4.5 to 0.7) due to genetic change (Figs 2 and 3), equivalent to −0.045 days per year (95% CI −0.100 to 0.018), −0.36 days per generation (95% CI −0.79 to 0.14), or −0.028 Haldanes (95% CI −0.062 to 0.01).

The less conservative BLUP regression estimated that evolution contributed −2.4 days (95% CI −4.9 to −0.2 days) to the phenotypic change over the study period. The STS estimated a change of −4.9 days (95% CI −10.6 to −0.7) over the study period (the estimate is the additive genetic covariance between parturition date and relative LBS, after back-transformation to days) (Fig 3). See S3 Table for raw estimates on the scale of 100×log(parturition date).

Nine percent of the simulations of genetic drift generated an advance as large or larger than the change estimated from the conservative BLUP linear regression (using the posterior mode for the BLUPs trend as a point of comparison; see Fig 3). Inbreeding tended to delay parturition date (S1 Table), and given that the estimated pedigree inbreeding inevitably increased over time with increasing pedigree depth [54], there was marginal evidence of inbreeding postponing parturition date by 0.38 days (95% CI −0.04 to 1.01) over the study period, thus opposing the phenotypic trend. However, this prediction may be spurious, because the increase in inbreeding coefficient was an artifact of estimating inbreeding from a pedigree [54]. Re-running the model without inbreeding led to almost identical estimates for all other parameters. The effect of gene flow (the proportion of immigrant genotype) was uncertain (S1 Table), and its overall predicted effect over the study period was a change of 0.15 days (95% CI −0.34 to 0.72).

### Nongenetic contributions to phenotypic change

**Fig 4:**
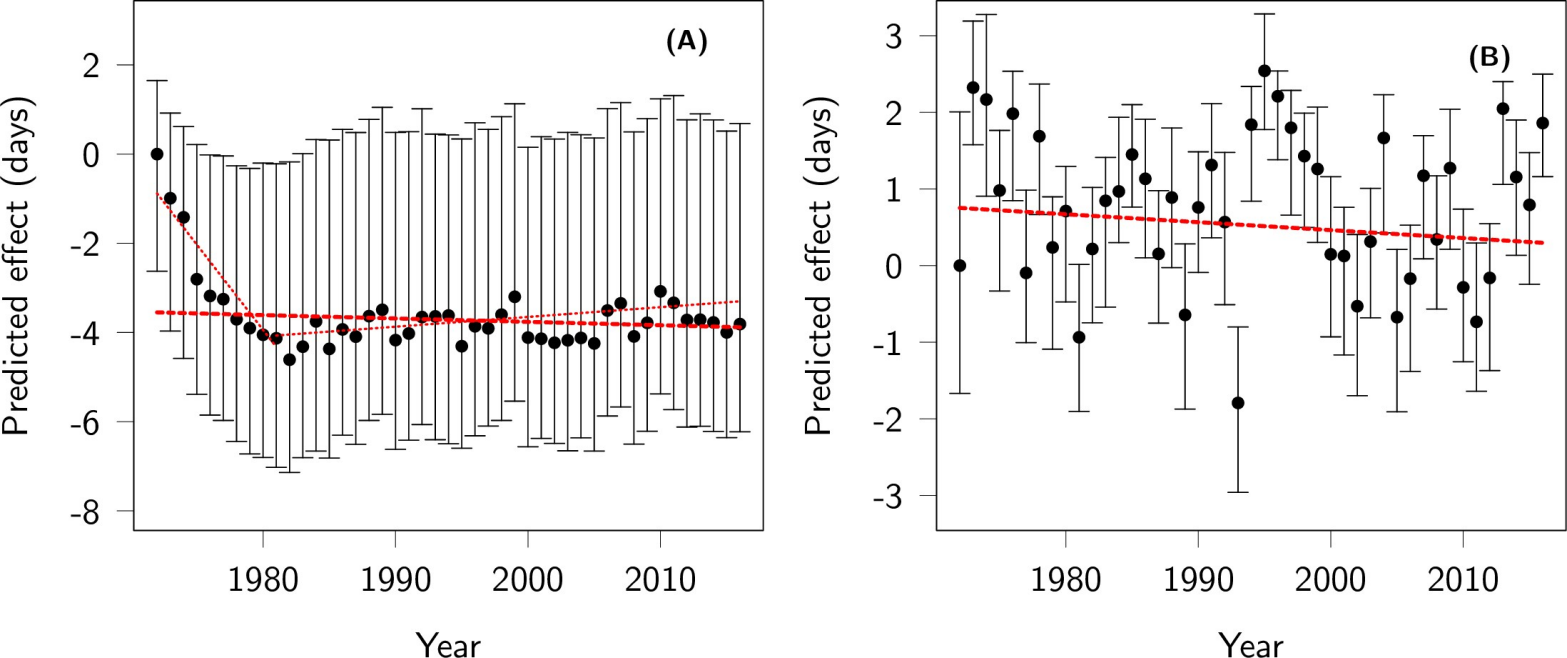
Effects of population structure on parturition date. Predicted effect of (A) age structure and (B) female reproductive status on parturition date across years. The origin of the y-axis is arbitrarily set to the predicted effect in the first year. The red thick dashed lines represent the net effect of changes in age structure and female reproductive status on parturition date reported in the text. The thin dotted lines in (A) represent the effect of changes in age structure before, and after, 1981, respectively. Code for this figure is on page 27 of S1 Code.

As in previous work [41], we found that mature females tended to give birth earlier than younger females, but very old females gave birth the latest (S2 Text). The effects of changes in the age structure on mean parturition dates tended to be in the opposite direction to the observed phenotypic change: during the first 10 years of the study, the mean age of females in the study increased steadily, pushing towards earlier mean parturition dates (−3.68 days [95% CI −5.63 to −1.92] from 1972 to 1981). For the rest of the study, the change in age structure tended to delay mean parturition date slightly (0.57 days [95% CI 0.39 to 0.71] from 1982 to 2016). Over the study period, the change in age structure had a predicted net effect of −0.58 days (95% CI −1.67 to 0.40) (Fig 4A). Changes in female reproductive status had a fluctuating effect on parturition date (Fig 4B), with an uncertain total effect over the study period of −0.32 days (95% CI −0.87 to 0.17). Offspring sex had no clear effect on parturition date, and since sex ratio at birth remained relatively stable over the study period (despite an early decline in the proportion of males [57]), this parameter is predicted to have had a small overall effect (−0.04 days [95% CI −0.18 to 0.04).

Warmer temperatures during the previous rut season tended to advance parturition date, with an overall effect of −1.40 days (95% CI −3.05 to 0.50) over the study period. The effect is less clear than the −2.4 days reported by Froy and colleagues [42] most likely because our model contains a covariate for year while Froy and colleagues [42] did not. When we remove the year covariate, we obtained an estimate of −2.56 days (95% CI −5.23 to −0.69).

**Fig 5:**
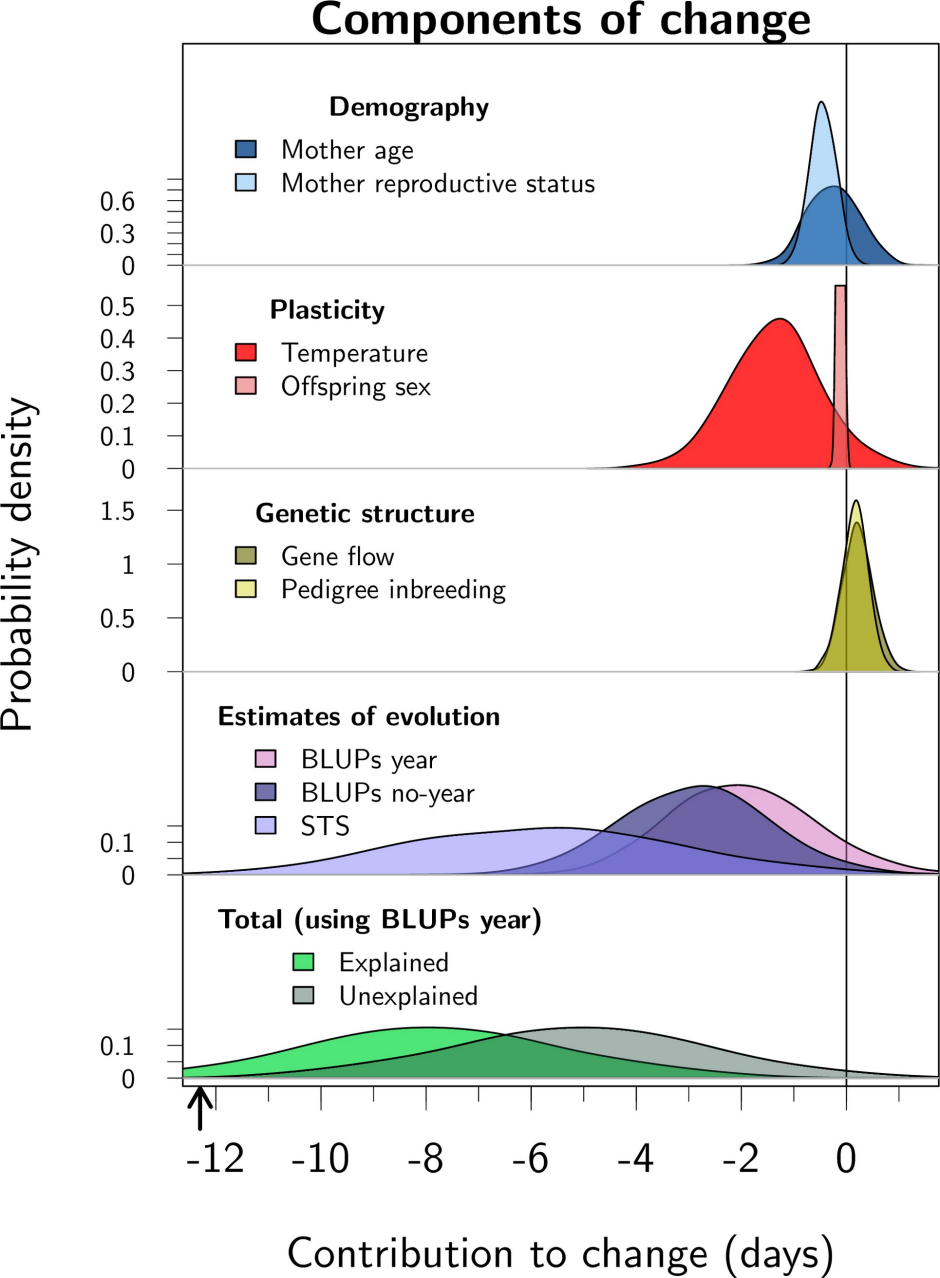
Posterior distributions of the components of change in parturition date over the study period. All estimates are derived from the univariate animal model described by Eq 1 (except for STS, derived form the bivariate animal model). Unlike other distributions, those in the row “Estimates of evolution” relate to a single component of change, estimated in 3 different ways: “BLUPs year” is the conservative BLUP regression with offspring birth year fitted as a fixed effect, “BLUPs no-year” is the BLUP regression without offspring birth year fitted as a fixed effect, and “STS” is the secondary theorem of natural selection using the additive genetic covariance between parturition date and fitness. To accommodate strong differences in the uncertainty around component estimates, the scale of the y-axis differs among rows, and the density of the component “Offspring sex” was truncated. The black arrow below the x-axis indicates the observed total change of −12.3 days. Code for this figure is on page 53 of S1 Code. BLUP, best linear unbiased predictor; STS, secondary theorem of natural selection.

Fig 5 summarises all the components of change described earlier. Altogether, these effects captured a predicted change of −7.98 days (95% CI −12.85 to −3.22) over the study period. This leaves an unexplained change of −4.99 days (95% CI −9.76 to −0.13). Given the model specification, the unexplained fraction must capture persistent changes in maternal effects, individual-level (referred to here as “permanent”) environment effects, and various other effects of phenotypic plasticity (other than that due to sex of the offspring and mean temperature during the rut period), which were not explicitly accounted for in the model.

## Discussion

In the Isle of Rum red deer study population, average parturition dates have advanced 12.3 days over the last 4 decades. Previous research has identified the contribution of plastic changes in response to warming temperatures to this change [42]. Here we have shown that adaptive evolution likely played a role as well (Fig 3), and we have quantified the relative importance of demographic, plastic, and evolutionary changes (Fig 5). Subsequently, we discuss the significance of the results for the red deer population, as well as the strengths and challenges associated with the quantitative genetic study of evolution in wild populations.

Moyes and colleagues [40] identified the trend towards earlier parturition dates in the Isle of Rum red deer population and related a substantial component of the trend to local climate warming. In addition, within-individual plasticity is sufficient to explain the relationship between temperature and parturition date, and plasticity in response to increasing temperature explains some of the phenotypic change [42]. There is little evidence of variation among females in their plastic responses to temperature [42]. Therefore, the plastic response to temperature is unlikely to have changed (by genetic evolution or other means) over the study period, and a change in the strength of individuals’ plastic responses (reaction norm slopes) probably did not contribute to the change in mean parturition dates.

The present work reveals a major new aspect of the complex picture of the dynamics of parturition date in this population, by identifying a role for evolution concurrent with the previously identified plastic responses. We estimated that evolution for parturition date accounted for a total change of −2.1 days (95% CI −4.5 to 0.7) over the study period. This estimate relies on the modern and conservative version of BLUP regression, which accounts for criticisms made by Hadfield and colleagues [23] and Postma [22], in particular by including offspring birth year as a covariate. Taking this approach yielded a conservative estimate of genetic change that accounted for 15% of the observed phenotypic change. As expected, the less conservative alternative of not including year as a covariate gave a more rapid estimate of evolution: −2.4 days (95% CI −4.9 to −0.2 days). As a third method, the STS predicted an evolutionary change of −4.9 days (95% CI −10.6 to −0.7), which is more evolution than estimated by the 2 BLUP regressions. The STS may be more powerful than BLUP regression because the addition of a second trait (LBS) in the model provides more genetic information compared to a univariate approach. On the other hand, the STS, as applied here, has the disadvantage of assuming a log-normal distribution for fitness (LBS here) [62, 63]. Log normality is violated because LBS is zero-inflated, and the variance-covariance components may therefore be inaccurate. Thus, all 3 methods have possible weaknesses, and none is likely to perfectly estimate the true amount of evolution. Nevertheless, the 3 methods agree qualitatively, and the posterior distributions for the role of evolution largely overlap (Fig 3).

Our results suggest modest roles for changes in demographic structure. Shifting proportions of females of different reproductive status and ages had a predicted combined effect of −0.9 days (about 7% of the phenotypic change). These effects were also identified by Stopher and colleagues [41]. Changes among individuals, other than change in breeding values, therefore probably explain only a small (but non-negligible) fraction of the observed phenotypic change. However, summing all the effects estimated here still leaves a change of −4.99 days (95% CI −9.76 to −0.13) unexplained. Plastic responses to other environmental variables likely account for some of the remaining change, since we did not consider the response to any variables other than mean temperature during a 5-month period, and the sex of the offspring (which responds to the biotic and abiotic environment in a complex way [57]). In particular, other climatic variables such as temperatures during other times of the year, temperature variability, rainfall, and wind speeds probably affect reproductive traits in red deer [41]. In addition, the evolution of indirect genetic effects [69] may play a role. For instance, maternal genetic effects [70], which in this study would be genetic effects for how a mother influences the reproductive timing of her daughters, could evolve. However, maternal genetic effects and their possible contribution to phenotypic change are likely small in this system, because total maternal effects (which include both genetic and nongenetic maternal effects) account for less than 1% of phenotypic variance. Other types of social interactions that influence parturition may have a genetic basis that evolves, but such effects are difficult to study without a priori knowledge of the relevant individual interaction mechanisms [69].

The indication of evolution towards earlier parturition dates is consistent with previous work, which found the trait to be heritable [43] and under selection for earlier dates [44] in this population. Under ideal conditions, the product of heritability and strength of selection predicts the evolutionary response to selection [46, 71]. However, this “breeder’s equation” frequently fails to give reliable predictions in wild populations [47, 71]. Simultaneous selection on genetically correlated traits is likely to be a major cause of this failure, because fitness is generally causally affected by many traits, and genetic correlations are common [48].

Here, however, we obtained a reasonable match between the estimated rate of evolution (i.e., the estimated change in the additive genetic component of parturition date) and the response to selection predicted by the breeder’s equation, both in its univariate and in its bivariate forms. We cannot discard the possibility that this match might be in part a coincidence; for instance, if the indirect response to selection on a trait not included in the analysis pulled evolution in one direction but genetic drift pulled it back to match the observed rate of evolution. Other factors may have biased the prediction of evolution and made the match coincidental. In particular, although LBS is widely used as a measure of fitness in evolutionary ecology, it is generally not exactly the quantity maximized by natural selection when generations overlap and the environment and the population structure vary [72, 73]. Moreover, we cannot measure parturition date in females that died before reproducing, which creates a missing fraction in the estimation of selection [53]. It is possible that those females who died early are not “missing at random” with respect to genetic merit for parturition time, meaning that the true response to selection may differ from that predicted. This second problem is in part solved by the calculation of the STS (using the additive genetic covariance between parturition date and LBS) because all individuals have a breeding value for parturition date even if they never expressed the trait. The STS is therefore estimated with a much smaller missing fraction, consisting only of local individuals that do not have a LBS record (e.g., aborted embryos). The STS clearly predicted negative evolution of −4.9 days (95% CI−10.6 to −0.7), reinforcing the idea that the true selection on parturition date favours earlier dates. However, it is possible that the STS is not only related to the direct selection on parturition date but is also influenced by selection on other traits genetically correlated to parturition date. In summary, there are several potential factors that may bias the estimate of selection in one way or another, and so we interpret the estimates with caution. However, our different analyses all point towards a role of selection in advancing parturition date.

We estimated evolution and selection averaged over the study period to obtain the total evolution and response to selection expected over the period. However, if an increase in temperature explains selection for earlier parturition, it is possible that selection has intensified in more recent years and that selection was strongest in warmer years (e.g., [74]). The multivariate models we used to estimate selection allowed the estimation of selection by correcting for fixed and random effects in both parturition date and fitness traits but are not well suited to estimating changes in selection. Future work could investigate the selective scenario by estimating the interaction between parturition date and temperature in a generalized linear model of fitness, but care should then be taken to correct for the effect of time or other selectively irrelevant aspects of variation in parturition date.

A changing climate is probably not the only selective agent relevant to the evolution of parturition date in this red deer population. Indeed, selection was stronger among females that died of natural causes (with a predicted response to selection of −2.0 days) than among the shot females only (+0.10 days). Culling may alter selection on parturition date, possibly by removing females from the population at random with respect to their potential parturition dates, thus diluting natural selection. Alternatively, culling may not be random with respect to parturition date but somehow exert a type of artificial selection for later parturition dates that thus effectively opposes natural selection. Either way, culling may be slowing down the adaptive response to natural selection in the population. If confirmed, this result would add to the list of evolutionary consequences of culling [15, 66].

The response to selection predicted by the breeder’s equation corresponds to the rate of evolution estimated from the trend in breeding values as well as from the STS. However, this genetic change is much less than the observed phenotypic change. The mismatch is not surprising given that several mechanisms of phenotypic change, with a genetic basis or not, have been identified on the Rum red deer population (in our analyses presented here as well as in [41, 42]). More generally, our results illustrate how phenotypic change can be simultaneously due to both plastic and genetic changes [6, 8, 47]. Plastic changes in response to climate change appear common in natural populations, but that does not preclude concurrent evolutionary change in response to climate change [14]. Evolutionary changes are substantially more difficult to infer than plastic changes, and to date, few appropriate tests of evolution have been performed [14, 18, 39, 47]. Moreover, here as in other systems, large contributions of evolution may represent only part of the overall phenotypic trend [66, 75]. Thus, evidence for plastic responses should not be taken as reason to dismiss a role for genetic change [76, e.g.], nor the other way around. As another side of the same coin, our results highlight the insights that a quantitative genetic perspective brings to the study of phenotypic trait dynamics. As outlined earlier, the breeder’s equation often fails to predict phenotypic change in the wild. One possible explanation for this failure is “cryptic evolution,” in which genetic change is hidden by plastic changes [47]. Our results illustrate that a simple application of the breeder’s equation can work, but that it should be tested by comparison with estimates of genetic changes, not of phenotypic changes.

## Methods

### Ethics statement

This work takes place under a UK Home Office Project Licence under the Animals (Scientific Procedures) Act 1986 as amended. Licence number 70/8818 is held by J. M. Pemberton. The field work takes place with the permission and assistance of Scottish Natural Heritage, which manages the Isle of Rum National Nature Reserve.

### Study population

We used data from a long-term study of the unmanaged population of red deer living in the North Block of the Isle of Rum, Scotland (57°01′ N, 6°17′ W), for the years 1972–2016. Within the ca. 12 km^2^2 of the study area, calves are marked with ear tags (and a collar for females) shortly after birth, in order to record detailed life histories of individuals throughout their lives [50]. DNA was obtained from ear punches, postmortem tissue, and cast antlers. The population pedigree was reconstructed from single nucleotide polymorphisms as in Huisman and colleagues [51], using the R package SEQUOIA [52].

We studied the selection and genetics of parturition date, the date on which a female gave birth to a calf in a given year. We therefore focus on females, because males do not express the trait of parturition date—though they may affect it, in both genetic and nongenetic ways. We estimated the selection on parturition date among males in S4 Text and found its direction to be unclear and its magnitude to be likely weak, so we did not consider it further in the calculations that follow. However, males were retained in the pedigree and contributed to the calculation of quantitative genetic parameters by informing the relatedness between individuals. We included females that were still alive, even though their lifetime fitness was still unknown, in order not to introduce a fraction of individuals missing “not at random” with respect to fitness and parturition date [53]. However, excluding living females (12% of individuals) from the analysis gave slightly stronger estimates of selection but did not affect results qualitatively (see S2 Fig).

Death is usually due to natural causes, as there has been no culling in the study area since 1973, but individuals are occasionally shot when they visit areas surrounding the study area. Mortality due to culling may exert a kind of artificial selection that studies of natural selection may want to exclude. However, our goal here was to understand the causes of phenotypic change, be they natural or artificial. We therefore retained culled females in our main analyses. These shot females represented a small but nontrivial portion (15%) of the data set (Table 1). Therefore, in a subanalysis, we also considered selection only among females that died of natural causes (i.e., excluding both shot females and females still living) and discuss the consequences of culling for selection and evolution in this system.

**Table 1:** Sample sizes for LBS and parturition date for years 1972–2016. All numbers refer to females. Parturition date is measured repeatedly on individuals, whereas LBS is measured over a lifetime, and there is only one measure per individual. All females in the study population have an LBS record, including those that died as calves and therefore did not breed and do not have records for parturition date.

Data type	Number of	Excluding shot	Shot	Total
Parturition date	Individual	582	158	740
	Records	2,921	463	3,384
LBS	Individuals	1,614	282	1,896

**Abbreviation:** LBS, lifetime breeding success

Parturition date was measured as the number of days after May 1, because virtually no parturition occurs before that date (except for 2 outliers from late March, which were discarded prior to analysis). Values were (natural-)log-transformed in order to obtain residuals with distributions close to Gaussian. The logged values were multiplied by 100 for reporting convenience (in particular, variance components would have been of the order 10^−5^ without this second step). The working phenotype in all models was therefore *z* = 100log(*B*), where *B* is the parturition date in number of days after May 1. Results were converted back to days (see S1 Text for details of the back-transformation process) to facilitate biological understanding. We report results using untransformed data in S5 Text. In brief, we obtained similar results from animal models fitted to the untransformed parturition date data, but these models performed relatively poorly (skewed residuals and poor Markov Chain Monte Carlo [MCMC] mixing), which may impair the reliability of estimates.

Data and code to reproduce all analyses are provided in S1 Code and S1 Data.

### Quantitative genetic analysis

#### Univariate animal model

We fitted a univariate animal model of female (log) parturition date in order to estimate heritability and change in breeding values [21, 45]. The fixed effects in the model were as follows: the sex of the offspring; the female’s “reproductive status,” which can take 5 values to represent different recent reproductive history (“naive,” “true yeld,” “summer yeld,” “winter yeld,” and “milk hind” [42]); the female’s age in years (first- and second-order polynomial, which provided a good fit to the data, see S2 Text); a continuous covariate of the expected proportion of immigrant versus resident genes in each female to model gene flow into the population (“genetic group,” see S3 Text); the offspring birth year as a continuous variable (see next section for details); the female’s pedigree-based inbreeding coefficient [54, 55] calculated using the R package MCMCglmm [56]; and, finally, air temperature around the rut period on the year preceding a parturition (mean daily maximum temperature between 17 July and 20 November, following [42]). The covariate temperature aims to capture the plastic response to temperature, in particular through its effect on the timing of mating, as shown in [42]. The factor sex of the offspring also captures a plastic response, albeit indirect and complex, because sex of the offspring is affected by variation in population density, winter rainfall, and presumably other factors related to nutritional stress in females [57]. Population density, estimated as the number of resident adult females in a given year, had a significant effect on (log) parturition date at the beginning of the study period (e.g., between 1974 and 1987, [50]), but we found no effect in the full data set (slope −0.38, standard error 0.63) and therefore excluded this variable from the analyses.

The random effects decomposed the variance not accounted for by fixed effects into 6 components, as follows: additive genetic variance, “permanent environment” variance (estimable from the repeated measures of the same females across multiple years, [58]), maternal effects variance (i.e., associated with the mother of the breeding female, and hence grandmother of the new calf), variance associated with the offspring birth year, variance associated with the breeding female’s (i.e., mother of the calf) cohort, and residual variance.

Thus, the model of (log) parturition date (*z*) of female *i* in year *j* can be written

$$z_{ij}=\mu +X^{T}b+a_{i}+p_{i}+m_{i}+c_{i}+y_{j}+r_{ij}$$ (1)

where *μ* is an intercept; ***X*** is a vector of fixed predictors (including the covariate offspring birth year); ***b*** is a vector of fixed effects; *a*, *p*, *m*, *y*, and *c* are random effects with which to estimate the variance associated with additive genetic values (i.e., breeding values), permanent environment, maternal identity (i.e., grandmother of calf), female’s cohort, and offspring birth year, respectively; and *r* is the residual. The breeding values (*a*) are normally distributed as $\left(a_{1},\ldots ,a_{n}{)}^{T}∼N(0,\sigma_{A}^{2}(z)A\right)$, where $\sigma_{A}^{2}(z)$ is the additive genetic variance for (log) parturition date, n is the number of females, and ***A*** is the relatedness matrix between individuals. The heritability of (log) parturition date was estimated as $\sigma_{A}^{2}(z)$ divided by the total phenotypic variance.

We used this animal model to estimate the individual-level repeatability (in addition to the heritability) of (log) parturition date. Repeatability was defined as the proportion of total phenotypic variance accounted for by all effects that are constant for an individual: inbreeding and genetic group (the variance explained by these fixed effects was calculated following [59]) as well as additive genetic variance, permanent environment variance, maternal variance, and female’s cohort variance (i.e., all random effects but offspring birth year and residual).

We ran all models in a Bayesian framework using the R package MCMCglmm [56] with Gaussian errors for (log) parturition date. We report point estimates as posterior modes and estimation uncertainty as 95% highest posterior density credible intervals. For this univariate model, we used the default inverse-gamma priors for variance components, with shape and rate parameters both equal to 0.001 (equivalent to a variance and degree of belief of 1 and 0.002, respectively). We ran models for 130,000 MCMC iterations, with a burn-in of 30,000 and thinning of 100, and checked mixing and convergence by visual inspection of trace plots for all parameters. All code for analyses is provided in S1 Code.

### Selection

We estimated selection acting on (log) parturition date by assessing the association between a female’s fitness and her propensity for (log) parturition date corrected for various effects. We measured fitness as LBS (*W* in equations), which is the number of offspring produced by an individual, calculated for all females in the database, whether or not they survived to breeding and therefore also had parturition records.

Selection was estimated using a model of the covariance between (log) parturition date and fitness. We used a bivariate generalized linear mixed model, with LBS modelled as an overdispersed Poisson trait (with log-link function) and (log) parturition date (*z*) modelled as a Gaussian trait. This model can be written as

$$[z,W]∼Xb+D_{1}m+D_{3}c+D_{2}y+D_{4}p+Ir$$ (2)

where ***Xb*** represents fixed effects (only an intercept and genetic group for fitness, and the same fixed effects for log-parturition date as above, except for temperature); ***m***, ***c***, ***y***, and ***p*** are random effects associated with maternal effects (the identity of the mother of the breeding female), the female’s cohort (i.e., her year of birth), the year of calving, and the individual female’s identity (or “permanent environment” effect, because of the repeated measures), respectively. ***D*** matrices link random effect levels to observations, and ***Ir*** represents the residuals.

Note that *W* is only measured once for each individual, unlike the repeated measures on parturition date (*z*). For *W*, variance components are therefore null for ***y*** (the year of calving) and ***p*** (the “permanent environment” component of a trait, derived from repeated measures on an individual). MCMCglmm accommodates this difference in replication between the 2 traits by allowing the individual-level random effect ***p*** for the replicated trait (parturition date) to covary with the residual variance ***r*** of the nonreplicated trait (fitness), thus providing a covariance between the repeatable part of an individual’s parturition date and her fitness (for a comparable example, see [60]).

The selection differential on (log) parturition date was calculated as the sum of this individual-level covariance, plus the maternal-effect covariance between (log) parturition date and fitness (i.e., covariance among effects of the breeding females’ mothers on their daughters’ log-parturition dates and fitness). Selection differentials characterize the within-generation change in phenotype due to selection. We therefore standardized this value by generation time (8 years, see [50]) to be expressed in rate of change per year, or in total change over the study period. Selection differentials were divided by 2 because the covariances were estimated from females only. Males do not express the trait but nevertheless carry genes relevant to parturition date in females. Selection on parturition acts on only half of the population, and the expected response to selection is half that predicted from the strength of selection in females. As outlined earlier, we considered the possibility that males are nevertheless selected for the trait in S4 Text, but because we did not find support for any selection through males, we did not consider it further. We also estimated a selection gradient [61], calculated as the selection differential divided by the corresponding variance (i.e., the sum of the individual-level and mother-level variance components for parturition date).

When expected fitness follows a log-normal distribution, selection parameters can be equivalently calculated on the scale of the data using relative fitness or on the log-scale using absolute fitness [62, 63]. Because our model uses a log-link function for absolute LBS, parameter estimates involving LBS are on the latent scale, but these are directly interpretable as selection differentials and selection gradients relating to relative fitness on the data scale.

For multivariate models, we used parameter-expanded priors for variance components with degree of belief equal to 0.002 (for random effects fitted for a single trait) or 2 (for random effects fitted to both traits), working mean equal to 0, and variance equal to 1,000. For residual variances, we used the same degrees of belief (0.002 or 2) but did not use parameter expansion since this is not possible in MCMCglmm. Estimates of selection were identical when using inverse-gamma priors instead of parameter-expanded priors (S1 Fig). We ran these models for 260,000 MCMC iterations, with a burn-in of 60,000 and thinning interval of 200.

#### Univariate and multivariate predictions of evolution

The response to selection (the per-generation change in the mean value of the trait) was predicted as the product of the heritability and the selection differential for (log) parturition date, following the univariate breeder’s equation [46]. The equation was applied to estimates from the model of (log) parturition date data, and the predicted response was subsequently back transformed to days (see S1 Text). Calculations were done on the MCMC posterior distributions of the heritability and the selection differential, in order to propagate the uncertainty in parameter estimates. The univariate breeder’s equation ignores the fact that the adaptive evolution of a focal trait depends not only on direct selection on that trait but also on selection on any other traits that are genetically correlated with the focal trait [49]. This assumption may explain in part the common mismatch between predicted and observed evolution in natural populations [48], but it can partly be relaxed by incorporating analyses of relevant associated traits and estimating multivariate selection and genetic covariances: the multivariate response to selection can then be predicted as the product of the additive genetic variance-covariance matrix ***G*** and the vector of multivariate selection gradients *β* (*Δ****Z*** = ***G****β*) [49, 61].

In the Rum red deer study population, a calf’s birth date is correlated with its birth weight [41, 50], a trait also under selection [44]. We therefore applied a bivariate breeder’s equation to parturition date and calf birth weight to estimate the effect of indirect selection on the predicted evolutionary response of parturition date to selection.

We extended the animal model of (log) parturition date (Eq 1) to a bivariate animal model of (log) parturition date and offspring birth weight, using the same fixed effects and random effects for both traits. Note that, in this model, the calf’s birth date and birth weight (*bw*) are both being treated as the phenotype of the mother; the treatment of offspring birth weight as a trait of the mother is justified by the observation that more than 90% of the additive genetic variance in birth weight is maternal genetic variance rather than direct genetic variance [58]. This model estimated an additive genetic covariance between the 2 traits, *σ*_*A*_(*z*,*bw*), which can be divided by the square root of the product of the 2 additive genetic variances, $\sigma_{A}^{2}(z)$ and $\sigma_{A}^{2}(bw)$, to obtain a genetic correlation.

Finally, we extended the bivariate selection model (Eq 2) to a trivariate model also including offspring birth weight (along with log-parturition date and LBS). For birth weight, we used the same fixed and random effects as described earlier for (log) parturition date. We summed the appropriate covariances of the phenotypes with LBS to obtain a vector of selection differentials ***s***. We summed the appropriate variances and covariances for the 2 phenotypes to obtain a phenoytpic variance-covariance matrix ***P***. We then applied ***P***^−1^***s*** to obtain *β*_*z*_, the direct selection gradient on (log) parturition date corrected for the indirect selection on birth weight, and *β*_*bw*_, the direct selection gradient on birth weight corrected for the indirect selection on (log) parturition date [64]. The response to selection in (log) parturition date could then be calculated as $\beta_{z}\sigma_{A}^{2}(z)+\beta_{bw}\sigma_{A}(z,bw)$ [61].

We also expressed predicted rates of evolution in Haldanes, i.e., in units of standard deviation per generation [65]. We did not express the results in Darwins (i.e., change in log mean phenotype per million years) because parturition dates have no natural zero point, and therefore mean standardisation is not meaningful here.

### Components of change

#### Genetic change

Using the univariate animal model of (log) parturition date containing year as a covariate (see next paragraph), for each posterior sample, we fitted a linear regression of BLUPs (i.e., model predictions for the values of a random effect’s levels) for individual females’ breeding values against the mean birth year of their offspring. This generates a posterior distribution for the slope of change in mean breeding value [23]. In addition, to visualize any potential nonlinearity in genetic change, we fitted a penalized thin plate regression spline (i.e., a smoothing function) of mean offspring birth year to the BLUPs for individual females’ breeding values for every posterior sample, thus generating the posterior distribution of the time dynamic of breeding values among birth years [66].

Changes in breeding values may indicate a response to directional selection, but they can also be produced by random fluctuations under nondirectional evolutionary models, such as genetic drift. To assess this possibility, we also compared the posterior distribution of the estimated change in breeding values to the change possible under genetic drift alone, using simulations conditional on the pedigree and on estimated additive genetic variance, as described in [23]. In general, breeding values predicted by animal model BLUPs are not equal to the true breeding values but are influenced by environmental random deviations [22]. As a consequence, a linear regression of BLUPs may confound genetic and nongenetic (e.g., plastic) change and may produce a biased estimate of evolution. This issue can be addressed by including year as a covariate in the animal model used to obtain BLUPs. Unfortunately, the solution returns a conservative estimate of the rate of change in breeding values, because the animal model ascribes some of the genetic change to the year effect [22]. We opted to report these primarily conservative estimates of evolution, based on an animal model that did contain offspring birth year as a covariate. However, we also refitted the animal model without a fixed effect for birth year and recalculated the change in BLUPs for breeding values estimated this way.

In an alternative approach, we estimated genetic change using the STS, from the additive genetic covariance between trait and relative fitness—also known as the Robertson-Price identity [60, 67, 68]. To this end, we expanded the bivariate model of parturition date and fitness described in Eq 2 by adding a random effect of additive genetic variance-covariance. Assuming log normality of LBS, the expected rate of evolution per generation in (log) parturition date can be estimated directly from the additive genetic covariance between (log) parturition date and LBS [63], divided by 2 to account for females making up only half of the population.

#### Other contributions to phenotypic change

Finally, we estimated the contributions of several other terms in Eq 1 to the trend in parturition date. We used Geber’s method [6, 8] to estimate the independent contribution of changes in the class structure of age and reproductive status, in plastic responses to temperatures and sex of the offspring, and the independent contribution of changes in levels of inbreeding (as assessed from the pedigree inbreeding coefficient) and gene flow (as assessed by the genetic groups effect) to the phenotypic change in parturition date. Briefly, this method estimates the contribution of change in a parameter mean ($\bar{k}$) to change in a trait mean ($\bar{z}$) as the product of the partial derivative of *z* on $k(\frac{\partial \bar{z}}{\partial \bar{k}})$ and the slope of *k* on time ($\frac{\Delta \bar{k}}{\Delta t}$). We applied the equation to each sample of the posterior distributions in order to propagate the uncertainty in the estimated trends. In addition to calculating the net effect through the study period, we calculated $\frac{\partial \bar{z}}{\partial \bar{k}}{\bar{k}}_{t}$ for each year *t* to visualize the dynamic of changes in effects through time graphically.

We did not use random effects to estimate nongenetic components of change because random effects other than additive genetic effects are linearly independent of years by construction. Any change in females’ maternal effects or permanent environment effects should be absorbed into the fixed effect of offspring birth year.

## Supporting information

S1 Text Back-transformations to estimate change in days. Method to convert parameters estimated from models using *z* = 100 *log*(parturitiondate) as a response variable to changes in unit of days.
(PDF)

S2 Text Fit of second-order polynomial of mother’s age. Justification for modeling the effect of mother’s age on parturition date as a second-order polynomial.
(PDF)

S3 Text Definition of genetic groups. Definition and calculation of genetic groups used to model gene flow from outside the study area.
(PDF)

S4 Text Selection among males. Estimation of selection for parturition date among males.
(PDF)

S5 Text Analyses of untransformed parturition date data. Heritability and genetic change estimated from animal models fitted to untransformed parturition date.
(PDF)

S1 Fig Sensitivity of the estimate of selection to priors. Posterior distribution of the estimate of selection for log-transformed parturition dates with 2 different priors. Selection was estimated using the model described in Eq 2 of the main text. The 2 priors gave indistinguishable modes, means, and credible intervals. MCMC mixing was a bit less good with the inverse-gamma prior, giving a less smooth posterior distribution. Code for this figure is on page 36 of S1 Code.
(PDF)

S2 Fig Selection among individuals culled or not culled. Posterior probability densities for selection differential estimated from the same model fitted to 3 data sets: (i) total population (solid blue line), (ii) total population excluding culled individuals (dashed green line), and (iii) culled individuals only (dotted red line). Vertical lines highlight posterior modes. Code for this figure is on page 51 of S1 Code.
(PDF)

S1 Table Univariate animal model estimates. Estimates for the fixed effects and random effects of the univariate animal model of 100 × log-transformed parturition date (Eq 1 in the main text).
(PDF)

S2 Table Phenotypic selection model estimates. Fixed effects and variance-covariance components estimated from the model of phenotypic selection described in Eq 2 of the main text. The permanent environment covariance (in bold) is the main parameter of interest as it is a selection differential for 100 × log-transformed parturition date. Note that the permanent environment variance for LBS is the residual variance for LBS since LBS is only expressed once per individual (see main text “Methods” for details).
(PDF)

S3 Table Bivariate animal model estimates. Fixed effects and variance-covariance components estimated from the bivariate animal model of LBS and 100 × log-transformed parturition date. The additive genetic covariance is the main parameter of interest as (from the STS) it is an estimate of expected evolution for 100 × log-transformed parturition date.
(PDF)

S1 Data Data necessary to reproduce all analysis. See README.txt for details.
(ZIP)

S1 Code R code to reproduce all results, tables, and figures in the main text and in Supporting Information. (PDF)

## Abbreviations

BLUP: best linear unbiased predictor
LBS: lifetime breeding success
MCMC: Markov Chain Monte Carlo
STS: secondary theorem of natural selection
