# Supplementary material for: The role of selection and evolution in changing parturition date in a red deer population
Source: PLoS Biol. 2019 Nov 5;17(11):e3000493. doi: 10.1371/journal.pbio.3000493 (PMC6830748; doi:10.1371/journal.pbio.3000493)
Supplement: S1 Text — Method to convert parameters estimated from models using z = 100 log(parturitiondate) as a response variable to changes in unit of days. (PDF) [file pbio.3000493.s001.pdf]

S1 Back-transformations to estimate change in days

Our analyses were all run on transformed values $z = 100\log(B)$, where B is the parturition date in number of days after May 1st. We used a linear regression of z on offspring birth year to predict the initial mean z ($\bar{z_0}$) in 1972 (assuming a linear change) and extracted the residual variance of that regression ($\sigma^2(\epsilon)$). Assuming that the residuals from this regression are normally distributed and their variance constant, B follows a log-normal distribution conditional on time. The mean of a log-normal distribution is $\exp(\mu + \frac{\sigma^2}{2})$ where μ and σ^2 are the mean and variance on the log-scale. Therefore we predicted the change in mean parturition dates across the whole study period as $\Delta\bar{B} = \exp\left(\frac{\bar{z_t}}{100} + \frac{\sigma^2(\epsilon)/10000}{2}\right) - \exp\left(\frac{\bar{z_0}}{100} + \frac{\sigma^2(\epsilon)/10000}{2}\right)$, where $\bar{z_t}$ is the model prediction of $\bar{z}$ (on the log-scale) at the end of the study period (year 2016). This back-transformation is imperfect because the relationship between z and years is not perfectly linear, but it approximately recovers the observed phenotypic change (non-transformed data: he estimate of change using raw means of non-transformed data was -12.32 days 95%CI [$-14.53; -10.10$] whereas that calculated from the back-transformed method described above was -12.11 days 95%CI [$-13.77; -10.35$]).
