# Supplementary material for: The role of selection and evolution in changing parturition date in a red deer population
Source: PLoS Biol. 2019 Nov 5;17(11):e3000493. doi: 10.1371/journal.pbio.3000493 (PMC6830748; doi:10.1371/journal.pbio.3000493)
Supplement: S2 Text — Justification for modeling the effect of mother’s age on parturition date as a second-order polynomial. (PDF) [file pbio.3000493.s002.pdf]

S2 Fit of second order polynomial of mother's age

During peer-review we were asked whether the second order polynomial of mother's age provided a good fit to the response variable ($z = 100 \log(\text{parturition date})$). Graphically, the second order polynomial provides a reasonable fit, capturing the initial decrease from age 2 to 5, the approximately stability from age 5 to 10, and the increase above age 10 (Fig. S2.1).

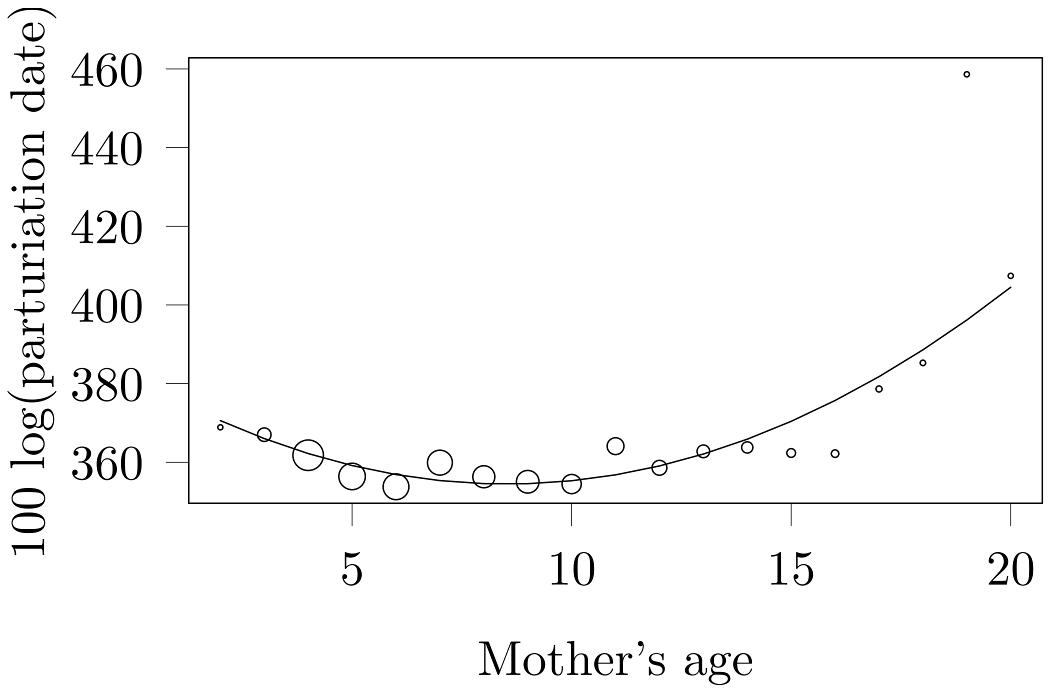

Figure S2.1: The diameter of points represents the square root of the sample size and are approximately proportional to the weight of each age in the regression.

In addition, we calculated AICc [1] values for different polynomial orders. AICc is a small sample size correction of Akaike Information Criterion, for which lower values indicate a better fit of the model, meaning a better ability to predict data from the population from which the sample used to fit model came from. As a rule of thumb, differences of less than 2 points in AICc are considered unimportant [1]. We obtained the following values:

- Order 1: 153.00
- Order 2: 141.59
- Order 3: 143.41

- Order 4: 143.24

which shows that the second order polynomial is a relatively good fit to the data (the best of the 4 models tested); although the fit of order 2, 3 and 4 polynomials is similar.
