# Supplementary material for: The role of selection and evolution in changing parturition date in a red deer population
Source: PLoS Biol. 2019 Nov 5;17(11):e3000493. doi: 10.1371/journal.pbio.3000493 (PMC6830748; doi:10.1371/journal.pbio.3000493)
Supplement: S3 Text — Definition and calculation of genetic groups used to model gene flow from outside the study area. (PDF) [file pbio.3000493.s003.pdf]

S3 Definition of genetic groups

Animal models measure genetic parameters relative to a base population, that is, the set of individuals that have no known parents [2]. In open natural populations, immigrants will have unknown parents and will be included in the base population by default, irrespective of their birth year. This conflation of cohorts within the base population can blur the estimation of genetic change across cohorts. In addition, changes in the number of immigrants might bias the estimation of evolution if immigrants differ genetically, for instance because they come from a population locally adapted to an environment different to that of the focal population [3, 4]. To account for these potential problems, we included genetic groups [5] in our animal models. We modeled genetic groups using the explicit fixed effect specification [4], and considered individuals with unknown parents to form two groups: local base population and immigrants. We defined the two genetic groups with the following rules: 1) The 'local base population' was defined as the 172 individuals with two unknown parents who were born before 1970 (when the intensive monitoring of the study population started), plus the fathers of calves born from a known mother and an unknown father (fathers contribute to genetic group values through the pedigree, although males have no phenotypes for parturition dates); 2) 'Immigrants' were defined as the 594 individuals born after 1970 from two unknown parents, and also unknown mothers of individuals born in any year with an unknown mother and a known father. We considered missing fathers as local because 2/3 of missing fathers are those of calves without a genetic sample, suggesting that the fathers are missing only because they could not possibly be inferred. Moreover, for the other third of calves with missing fathers, but with a genetic sample, the lack of paternity assignment must be due to the father lacking a genetic sample. We might therefore expect only $\frac{1}{3}\frac{1}{3} = \frac{1}{9}$ of calves without known fathers to be born from fathers who are truly immigrants. On the other hand, the lack of a maternal assignment (i.e. an unknown mother) almost certainly indicates an individual of immigrant origin.

For each individual, we therefore included a fixed effect of the estimated proportion of genotype descended from a 'base' versus 'immigrant' genetic group, estimated using the

R package nadiv [4]. This proportion was then included as a covariate fixed effect in the models of both parturition date and LBS.
