# Supplementary material for: The role of selection and evolution in changing parturition date in a red deer population
Source: PLoS Biol. 2019 Nov 5;17(11):e3000493. doi: 10.1371/journal.pbio.3000493 (PMC6830748; doi:10.1371/journal.pbio.3000493)
Supplement: S4 Text — Estimation of selection for parturition date among males. (PDF) [file pbio.3000493.s004.pdf]

S5 Selection among males

Although males do not express the trait parturition date, they may affect it, in both genetic and non-genetic ways, and there may be selection for these effects. We therefore explored the likely magnitude of selection among males in relation to parturition date.

We estimated selection among males as the individual-level covariance between LBS of males and their offspring birth date. We used LBS data for all known males in the population (2198 individuals) and all parturition dates with an identitied father (2134 parturition events from 302 different males). As for selection among females (equation (2) of the main text), we used a bivariate generalized linear mixed model, with LBS modelled as an over-dispersed Poisson trait (with log link function) and (log) parturition date (z) modelled as a Gaussian trait. This model can be written as

$$[\boldsymbol{z}, \boldsymbol{W}] \sim \boldsymbol{X}\boldsymbol{b} + \boldsymbol{D_1}\boldsymbol{f} + \boldsymbol{D_2}\boldsymbol{y} + \boldsymbol{D_3}\boldsymbol{c} + \boldsymbol{D_4}\boldsymbol{y} : \boldsymbol{p} + \boldsymbol{D_5}\boldsymbol{p} + \boldsymbol{I}\boldsymbol{r} \quad (1)$$

where $\boldsymbol{Xb}$ represents fixed effects (the same fixed effects for log-parturition date as above, and only an intercept and genetic group for fitness), $\boldsymbol{f}$, $\boldsymbol{y}$, $\boldsymbol{c}$, $\boldsymbol{y} : \boldsymbol{p}$, $\boldsymbol{p}$ are random effects associated with the female identity (the mate of the breeding male producing a given calf), the year of calving, the male's cohort, the interaction of year of calving by individual male's identity and finally the individual male's identity (or 'permanent environment' effect, because of the repeated measures), respectively. $\boldsymbol{D}$-matrices link random effect levels to observations, and $\boldsymbol{Ir}$ represents the residuals. The random effect $y : p$ is necessary in the male model (and was not in the females model of selection) because males can have multiple offspring on a given year. The residual unit in males is effectively the interaction of calf birth year, male identity, and female identity.

As above, note that W is only measured once for each individual, unlike the repeated measures on parturition date (z). For W, variance components are therefore null for $\boldsymbol{y}$ (the offspring birth year) and $\boldsymbol{p}$ (the permanent environment component of a trait, derived from repeated measures). `MCMCglmm` accommodates the difference in replication between the two traits by allowing the individual-level random effect $\boldsymbol{p}$ for the replicated trait (par-

turition date) to covary with the residual variance $\boldsymbol{r}$ of the non-replicated trait (fitness), thus providing a covariance between the repeatable part of an individual's phenotype and his fitness (for a comparable example, see [6]). In addition, to aid model convergence, we had to remove the covariance at the level of male's cohort and the variance in LBS among focal male's mother.

The variance-covariance components for the male selection model are given in Table S5.1. Birth date had a small repeatability among fathers (1.4%, 95%CI[0.9%; 2.0%]), leaving little room for selection. The selection differential among males (the individual-level covariance) was estimated at -0.36 days but with a high degree of uncertainty (95%CI[-0.93; 0.40]). Assuming that the heritability of offspring birth date is at its maximal possible value (that is, heritability = repeatability), the univariate breeder's equation predicts a change of -0.01 days (95%CI[-0.04; 0.02]) due to response to selection in males over the study period.

Table S5.1: Variance-covariance components for male LBS and offspring parturition date

Random effect	Parameter	Posterior mode	95%CI
Male's cohort	Variance Parturition	194.761	[14.94 ; 335.75]
	Variance LBS	1.890	[0.7 ; 3.38]
Focal male's mate	Variance Parturition	143.613	[107.06 ; 183.17]
Offspring birth year	Variance Parturition	319.5	[51.31; 573]
Male ID × Offspring birth year	Variance Parturition	685.803	[556.86 ; 823.95]
**Permanent environment**	Variance Parturition	0.388	[0.09 ; 0.93]
	**Covariance**	**-0.681**	**[-1.99 ; 0.85]**
	Variance LBS	15.037	[11.17 ; 19.59]
Residual	Variance Parturition	344.069	[310.7 ; 378.88]
