# Supplementary material for: The role of selection and evolution in changing parturition date in a red deer population
Source: PLoS Biol. 2019 Nov 5;17(11):e3000493. doi: 10.1371/journal.pbio.3000493 (PMC6830748; doi:10.1371/journal.pbio.3000493)
Supplement: S5 Text — Heritability and genetic change estimated from animal models fitted to untransformed parturition date. (PDF) [file pbio.3000493.s005.pdf]

S8 Analyses of untransformed parturition date data

We re-fitted the univariate animal model (equation 1) to the raw (i.e. untransformed) data of parturition date and re-estimated heritability and the rate of evolution based on the BLUP regression. Heritability was estimated at 0.10 (95%CI $[0.06; 0.13]$). The change in breeding values was estimated at -1.7 (95% CI$[-4.0; 0.7]$) using the most conservative method, and -2.3 days (95%CI $[-5.7; 0.4]$) using the less conservative method. Thus all estimates coincide with those from the analyses of the log-transformed data, but the models using untransformed data performed relatively poorly (skewed non-Gaussian residuals and poor MCMC mixing) which may impair the reliability of estimates.
