# Supplementary figures and images for: The role of selection and evolution in changing parturition date in a red deer population
Source: PLoS Biol. 2019 Nov 5;17(11):e3000493. doi: 10.1371/journal.pbio.3000493 (PMC6830748; doi:10.1371/journal.pbio.3000493)

# Posterior distribution for selection

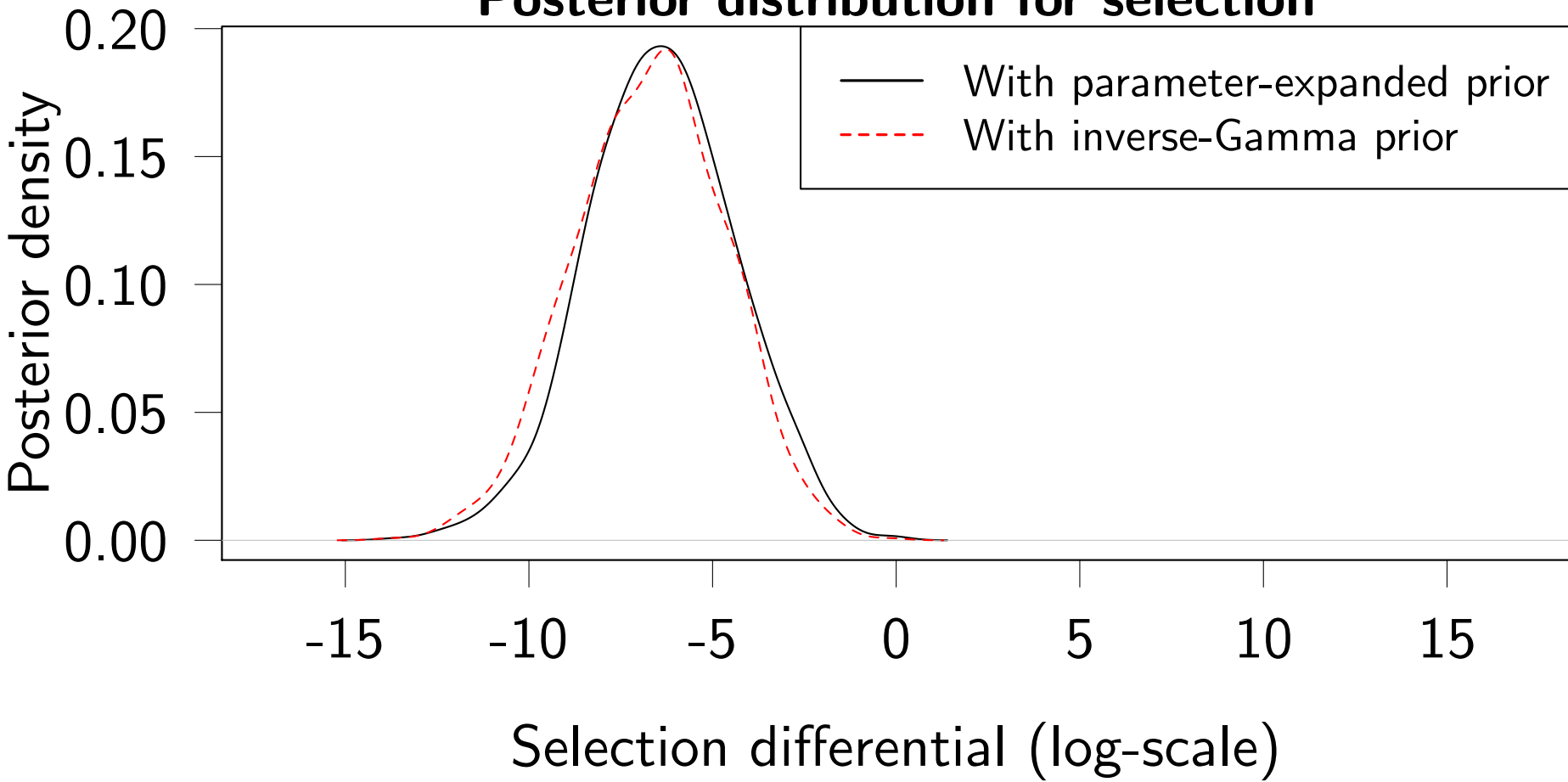

Supplement: S1 Fig — Posterior distribution of the estimate of selection for log-transformed parturition dates with 2 different priors. Selection was estimated using the model described in Eq 2 of the main text. The 2 priors gave indistinguishable modes, means, and credible intervals. MCMC mixing was a bit less good with the inverse-gamma prior, giving a less smooth posterior distribution. Code for this figure is on page 36 of S1 Code. (PDF) [file pbio.3000493.s006.pdf]

Posterior probability density

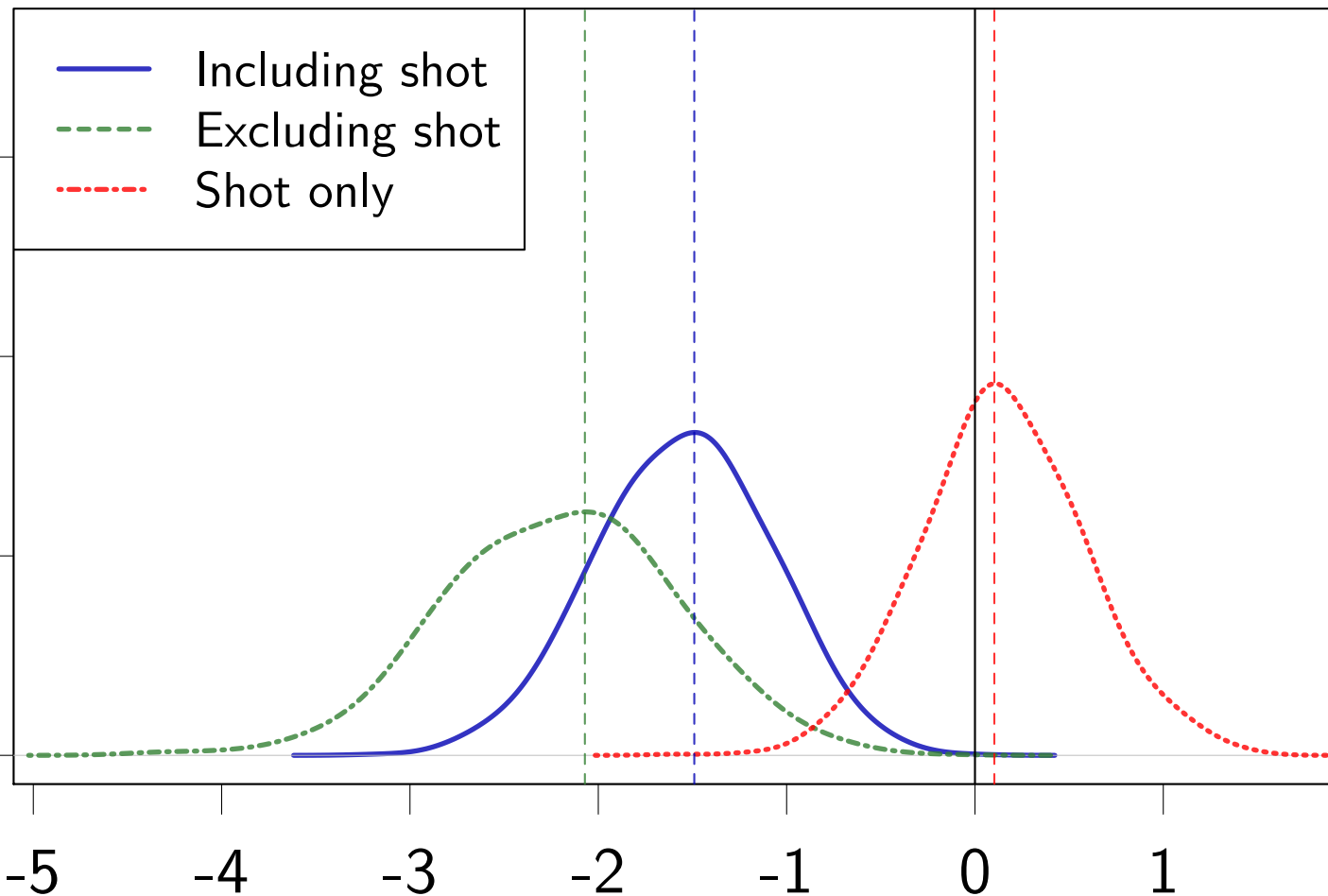

Selection differential (days/generation)

Supplement: S2 Fig — Posterior probability densities for selection differential estimated from the same model fitted to 3 data sets: (i) total population (solid blue line), (ii) total population excluding culled individuals (dashed green line), and (iii) culled individuals only (dotted red line). Vertical lines highlight posterior modes. Code for this figure is on page 51 of S1 Code. (PDF) [file pbio.3000493.s007.pdf]
