# Supplementary material for: The role of selection and evolution in changing parturition date in a red deer population
Source: PLoS Biol. 2019 Nov 5;17(11):e3000493. doi: 10.1371/journal.pbio.3000493 (PMC6830748; doi:10.1371/journal.pbio.3000493)
Supplement: S1 Table — Estimates for the fixed effects and random effects of the univariate animal model of 100 × log-transformed parturition date (Eq 1 in the main text). (PDF) [file pbio.3000493.s008.pdf]

S1 Table Univariate animal model

		Estimate	95% CI
Intercept*		406.82	[390.79 ; 420.52]
Female's inbreeding coefficient		89.39	[-7.22 ; 196.58]
Offspring Sex: Male		1.09	[-1.05 ; 3.20]
Female's Reproductive Status:	Naive	-13.53	[-19.26 ; -7.65]
	Summer Yeld	-27.01	[-31.46 ; -22.00]
	True Yeld	-21.56	[-25.43 ; -17.82]
	Winter Yeld	-0.60	[-6.19 ; 4.19]
Female's age		-6.64	[-9.20 ; -3.76]
Female's age squared		0.38	[0.24 ; 0.51]
Genetic group: Proportion immigrant		4.38	[-6.97 ; 16.48]
Temperature		-3.72	[-8.13 ; 1.50]
Calf birth year (covariate)		-0.45	[-0.74 ; -0.13]

Notes: *The intercept is defined for the year 1972.

	Estimate	95% CI
Additive genetic	208.3	[140.8 ; 282.9]
Permanent environment	5.9	[0 ; 40.6]
Focal female's mother	1.9	[0 ; 10.5]
Offspring birth year	112.3	[59.0 ; 189.1]
Female cohort	1.9	[0.0 ; 9.7]
Residuals	963.9	[912.7 ; 1011.0]
