# Supplementary material for: The role of selection and evolution in changing parturition date in a red deer population
Source: PLoS Biol. 2019 Nov 5;17(11):e3000493. doi: 10.1371/journal.pbio.3000493 (PMC6830748; doi:10.1371/journal.pbio.3000493)
Supplement: S2 Table — Fixed effects and variance-covariance components estimated from the model of phenotypic selection described in Eq 2 of the main text. The permanent environment covariance (in bold) is the main parameter of interest as it is a selection differential for 100 × log-transformed parturition date. Note that the permanent environment variance for LBS is the residual variance for LBS since LBS is only expressed once per individual (see main text “Methods” for details). (PDF) [file pbio.3000493.s009.pdf]

S2 Table Phenotypic selection model

Random effect	Parameter	estimate	95%CI
Female cohort	Variance Parturition	12.395	[0 ; 42.39]
	Covariance	1.133	[-15.47 ; 17.2]
	Variance LBS	9.080	[5.26 ; 12.93]
Focal female's mother	Variance Parturition	29.985	[0 ; 69.99]
	Covariance	-0.031	[-0.55 ; 0.34]
	Variance LBS	0.006	[0 ; 0.02]
Offspring birth year	Variance Parturition	157.481	[89.12 ; 250.5]
**Permanent environment**	Variance Parturition	219.364	[150.26 ; 290.62]
	**Covariance**	**-6.397**	**[-10.09 ; -2.44]**
	Variance LBS	0.987	[0.84 ; 1.13]
Residual	Variance Parturition	957.034	[909.11 ; 1006.62]

Trait	Parameter	estimate	95%CI
LBS (Poisson)	Intercept	-1.560	[-1.71 ; -1.4]
Log-transformed Parturition date (Gaussian)	Intercept	397.555	[384.91 ; 409.66]
	Offspring sex	1.824	[0.17 ; 3.75]
	Female's Reproductive Status: Naive	-16.811	[-21.11 ; -11.33]
	Summer Yeld	-27.313	[-30.91 ; -23.37]
	True Yeld	-23.434	[-26.79 ; -20.5]
	Winter Yeld	-0.803	[-5.18 ; 3.86]
	Female's age	-7.389	[-9.69 ; -5.43]
	Female's age squared	0.450	[0.35 ; 0.56]
