# Supplementary material for: The role of selection and evolution in changing parturition date in a red deer population
Source: PLoS Biol. 2019 Nov 5;17(11):e3000493. doi: 10.1371/journal.pbio.3000493 (PMC6830748; doi:10.1371/journal.pbio.3000493)
Supplement: S3 Table — Fixed effects and variance-covariance components estimated from the bivariate animal model of LBS and 100 × log-transformed parturition date. The additive genetic covariance is the main parameter of interest as (from the STS) it is an estimate of expected evolution for 100 × log-transformed parturition date. (PDF) [file pbio.3000493.s010.pdf]

S3 Table Bivariate animal model

Random effect	Parameter	estimate	95%CI
**Additive genetic**	Variance Parturition	211.257	[136.97 ; 284.08]
	**Covariance**	**-4.937**	**[-9.56 ; -0.58]**
	Variance LBS	1.077	[0.75 ; 1.4]
Female cohort	Variance Parturition	6.951	[0 ; 26.62]
	Covariance	-1.548	[-12.08 ; 6.65]
	Variance LBS	6.312	[3.54 ; 9.15]
Focal female's mother	Variance Parturition	26.383	[0 ; 71.51]
	Covariance	-0.016	[-0.71 ; 0.65]
	Variance LBS	0.020	[0 ; 0.08]
Offspring birth year	Variance Parturition	97.916	[43.81 ; 150.51]
Permanent environment	Variance Parturition	11.895	[0.17 ; 50.19]
	Covariance	-3.935	[-11.64 ; 1.56]
	Variance LBS	3.587	[3.16 ; 4.03]
Residual	Variance Parturition	962.298	[915.22 ; 1016.81]

Trait	Parameter	estimate	95%CI
LBS (Poisson)	Intercept	-2.204	[-2.92 ; -1.46]
	Genetic group	2.015	[1.56 ; 2.47]
	Inbreeding	-17.693	[-23.33 ; -11.88]
Log-transformed Parturition date (Gaussian)	Intercept	1149.012	[434.91 ; 1877.48]
	Genetic group	-2.046	[-11.09 ; 8.87]
	Inbreeding	59.318	[-34.12 ; 154.82]
	Female's Reproductive Status: Naive	-15.016	[-20.37 ; -9.43]
	Summer Yeld	-26.373	[-31.75 ; -21.46]
	True Yeld	-21.861	[-26.42 ; -16.97]
	Milk	0.563	[-4.03 ; 5.19]
	Female's age	-7.223	[-9.23 ; -4.91]
	Female's age squared	0.431	[0.32 ; 0.53]
	Offspring birth year	-0.372	[-0.74 ; -0.01]
