# Supplementary material for: The role of selection and evolution in changing parturition date in a red deer population
Source: PLoS Biol. 2019 Nov 5;17(11):e3000493. doi: 10.1371/journal.pbio.3000493 (PMC6830748; doi:10.1371/journal.pbio.3000493)
Supplement: S1 Code — (PDF) [file pbio.3000493.s012.pdf]

S1 Code for The role of selection and evolution in changing parturition date in a red deer population

Timothe Bonnet, Michael B. Morrissey, Tim H. Clutton-Brock, Josephine M. Pemberton & Loeske E. B. Kruuk

September 11, 2019

Contents

1 Data loading and preparation **2**

2 Sample size **3**

3 Phenotypic trend **4**

4 How to model parturition date? **8**

5 Animal models **10**
- 5.0.1 Model with temperature . . . 11
- 5.1 Heritability . . . 11
- 5.2 Trends in breeding values . . . 15
- 5.3 Conversion back to days . . . 16
- 5.4 Less conservative estimate of evolution . . . 19
- 5.5 Drift . . . 19
- 5.6 Changes in non-genetic components of parturition date . . . 21
  - 5.6.1 Age . . . 21
  - 5.6.2 Reproductive status . . . 24
  - 5.6.3 Offspring sex . . . 27
  - 5.6.4 Inbreeding . . . 28
  - 5.6.5 Immigrant origin . . . 29
- 5.7 Temperature . . . 30
  - 5.7.1 Residual change . . . 31
- 5.8 Model with untransformed data . . . 31

6 Selection analysis **35**
- 6.1 Fitness distribution . . . 35
- 6.2 Bivariate phenotypic model with LBS . . . 35
- 6.3 Considering parturition date as a male trait . . . 38
- 6.4 Bivariate animal model . . . 41
- 6.5 Multivariate breeder's equation . . . 43
  - 6.5.1 Animal model of calf weight and parturition date . . . 43
  - 6.5.2 Phenotypic multivariate selection . . . 44
  - 6.5.3 Calculation of selection differentials and gradients . . . 45
- 6.6 Difference between models including or excluding shot females . . . 48

7 Synthesis graphics **52**

1 Data loading and preparation

Just some graphical options:

```
szgr <- 2
szax <- 1.3
marr <- c(4, 4, 1, 1) + 0.1
setPar<-function(){
par(las=1,mar=marr, cex=szgr, cex.lab=szax , cex.axis=szax, lwd=2 ,pch=1, las=1)
}
```

Loading packages

```
library(MCMCglmm)#for everything good in life
library(lme4)#for quick check of model structures
library(MasterBayes)#for pedigree cleaning
library(pedantics)#for pedigree cleaning
library(mgcv)#for spline fitting
library(QGglmm)#for rescalling glmm parameters
library(nadiv)#for genetic groups
library(mixtools)#for ellipses
library(reshape2)#to melt birth and fitness datasets into one
```

Operating system and package versions.

```
sessionInfo()

## R version 3.6.1 (2019-07-05)
## Platform: x86_64-pc-linux-gnu (64-bit)
## Running under: Ubuntu 18.04.3 LTS
##
## Matrix products: default
## BLAS:   /usr/lib/x86_64-linux-gnu/blas/libblas.so.3.7.1
## LAPACK: /usr/lib/x86_64-linux-gnu/lapack/liblapack.so.3.7.1
##
## locale:
##  [1] LC_CTYPE=en_AU.UTF-8       LC_NUMERIC=C
##  [3] LC_TIME=en_AU.UTF-8        LC_COLLATE=en_AU.UTF-8
##  [5] LC_MONETARY=en_AU.UTF-8    LC_MESSAGES=en_AU.UTF-8
##  [7] LC_PAPER=en_AU.UTF-8       LC_NAME=C
##  [9] LC_ADDRESS=C               LC_TELEPHONE=C
## [11] LC_MEASUREMENT=en_AU.UTF-8 LC_IDENTIFICATION=C
##
## attached base packages:
## [1] grid      stats     graphics  grDevices utils     datasets  methods
## [8] base
##
## other attached packages:
##  [1] reshape2_1.4.3     mixtools_1.1.0     nadiv_2.16.0.0
##  [4] QGglmm_0.7.2       mgcv_1.8-28        nlme_3.1-141
##  [7] pedantics_1.7      MasterBayes_2.55   kinship2_1.6.4
## [10] quadprog_1.5-6     genetics_1.3.8.1.2 mvtnorm_1.0-10
## [13] MASS_7.3-51.4      gtools_3.8.1       gdata_2.18.0
## [16] combinat_0.0-8     lme4_1.1-21        MCMCglmm_2.29
## [19] ape_5.3            coda_0.19-2        Matrix_1.2-17
## [22] knitr_1.24
##
## loaded via a namespace (and not attached):
##  [1] Rcpp_1.0.1        plyr_1.8.4        compiler_3.6.1
```

```
##  [4] nloptr_1.2.1      highr_0.8         tools_3.6.1
##  [7] boot_1.3-23       evaluate_0.13     lattice_0.20-38
## [10] parallel_3.6.1    xfun_0.6          stringr_1.4.0
## [13] segmented_0.5-3.0 survival_2.44-1.1 tensorA_0.36.1
## [16] minqa_1.2.4       corpcor_1.6.9     magrittr_1.5
## [19] splines_3.6.1     cubature_2.0.3    stringi_1.4.3
```

Load basic data:

```
#data set with only females with parturition records
birthdata <- read.table(file="DataArchive/birth_fit_data.txt", header=TRUE,
                        stringsAsFactors = FALSE)

#load invAmatrix produced from pedigree by MCMCglmm::inverseA()
load("DataArchive/invAmatrix")

#fitness for all females, not only those with parturition records
birth_fit_data_AllLBS <- read.table(file="DataArchive/birth_fit_data_AllLBS.txt",
                                    header = TRUE, stringsAsFactors = FALSE)
```

2 Sample size

Parturtion data:

```
#records:
table(birthdata$MumShotOrNot)

##
## DeadNotShot      Living        Shot
##        2491         430         463

#individuals:
table(birthdata$MumShotOrNot[!duplicated(birthdata$MumId)])

##
## DeadNotShot      Living        Shot
##         468         114         158
```

LBS data all females

```
#records:
table(birth_fit_data_AllLBS$ShotOrNot)

##
## DeadNotShot      Living        Shot
##        3416         540         587

#individuals:
table(birth_fit_data_AllLBS$ShotOrNot[!duplicated(birth_fit_data_AllLBS$animal)])

##
## DeadNotShot      Living        Shot
##        1390         224         282
```

3 Phenotypic trend

```
Yrs<-min(birthdata$OffspBirthYear):max(birthdata$OffspBirthYear)
setPar()
trait.means<-tapply(birthdata$BirthDatePos,birthdata$OffspBirthYear,
                    mean,na.rm=T)
plot(Yrs,trait.means,xlab='Year',ylab='Parturition date (days)',pch=21,bg=1,
     ylim=c(10,70), cex=1.2)
mbd <- lm(BirthDatePos ~ 1 + OffspBirthYear,
                 data = birthdata, na.action = "na.omit")

smbd <- summary(mbd)
m0<-lm(trait.means~Yrs, na.action = "na.omit")
y<-predict(mbd, data.frame(OffspBirthYear=Yrs),interval="confidence")
lines(  Yrs,  y[,1] , col="red", lwd=5)
lines(  Yrs,  y[,2],lty=2 , col="red", lwd=4)
lines(  Yrs,  y[,3],lty=2 , col="red", lwd=4)
points(x = birthdata$OffspBirthYear, y = birthdata$BirthDatePos,
     col=rgb(0,0,0,0.1), pch=16, cex=0.8)
```

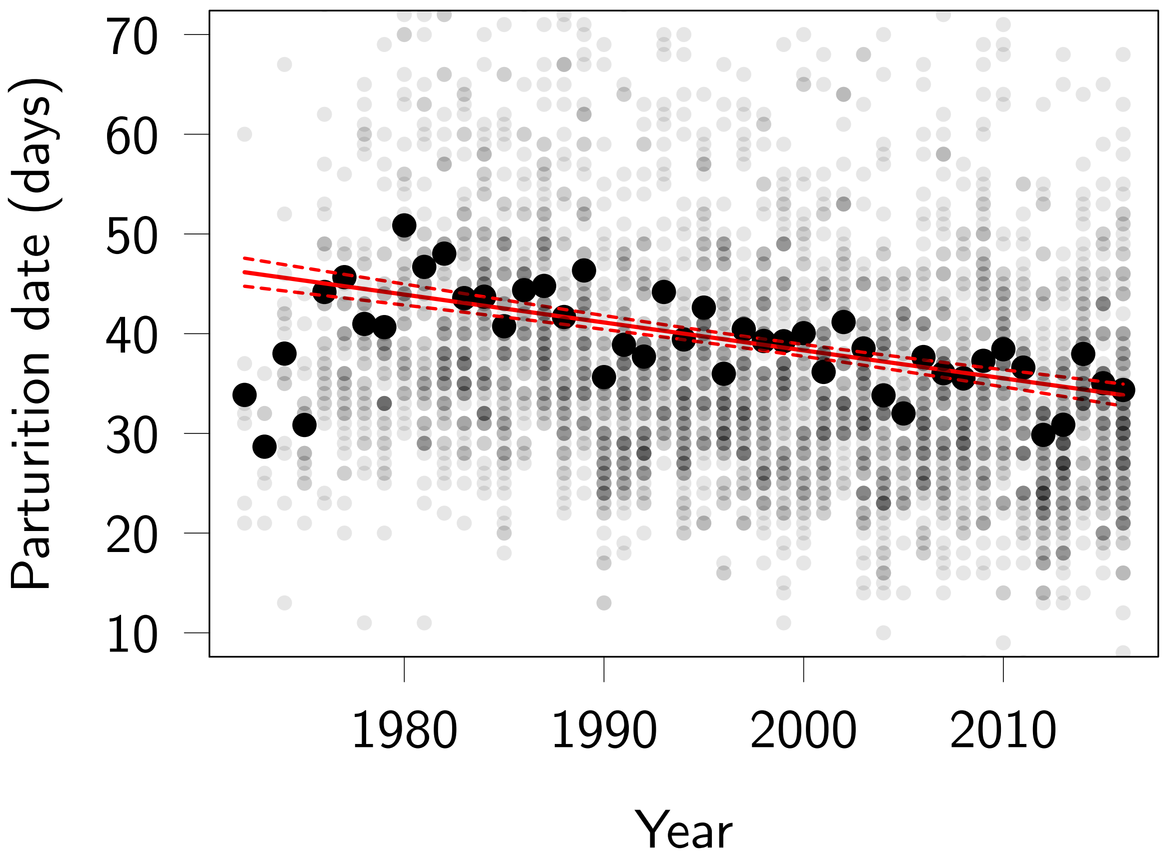

Figure 1: Phenotypic trends for parturition date. Large dots represent annual means, small dots individual data, and the red lines a linear regression of date on year with confidence interval. The proportion of data points included on the graph is 0.956

Prediction of the overall change:

```
#Estimate:
(max(Yrs)-min(Yrs))*coef(mbd)[2]

## OffspBirthYear
##      -12.31803

#Confidence interval:
CImbd <- confint(mbd)[2,]
(max(Yrs)-min(Yrs))*
  (CImbd[1])

##     2.5 %
## -14.53247

(max(Yrs)-min(Yrs))*
  (CImbd[2])

##   97.5 %
## -10.1036
```

We can also calculate the trend for the mean phenotype of each female on their mean year of presence.

```
datmeanfemale <- data.frame(meanbirthdate = tapply(birthdata$BirthDatePos,
                                                   birthdata$MumId, mean),
          meanyear = tapply(birthdata$OffspBirthYear,
                            birthdata$MumId, mean))
aggregatedreg <- lm(meanbirthdate~ meanyear, data = datmeanfemale)

(max(Yrs)-min(Yrs))*
  coef(aggregatedreg)[2]

##  meanyear
## -12.36486
```

A simple linear regression predicts a change over the study period of:

```
#point estimate:
(phenochange <- smbd$coefficients[2,1]*(Yrs[length(Yrs)] - Yrs[1]))

## [1] -12.31803

#95% CI:
(CIphenochange <- c(
    (smbd$coefficients[2,1]-2*smbd$coefficients[2,2])*(Yrs[length(Yrs)] - Yrs[1]),
(smbd$coefficients[2,1]+2*smbd$coefficients[2,2])*(Yrs[length(Yrs)] - Yrs[1])))

## [1] -14.57689 -10.05918
```

4 How to model parturition date?

Based on previous analyses, a reasonable model is:

```
mm0 <- lmer(BirthDatePos~1+OffspSex+(MumAge+MumAge2+MotherStatus)+
              OffspBirthYear+
              (1|OffspBirthYear) + (1|MumId),
            data=birthdata, na.action = "na.omit")
shapiro.test(summary(mm0)$residuals)

##
##  Shapiro-Wilk normality test
##
## data:  summary(mm0)$residuals
## W = 0.70822, p-value < 2.2e-16
```

But this gives not very nice residuals:

```
setPar()
hist(summary(mm0)$residuals, freq = FALSE, xlab="Residual value",
     main="Residual probability density")
```

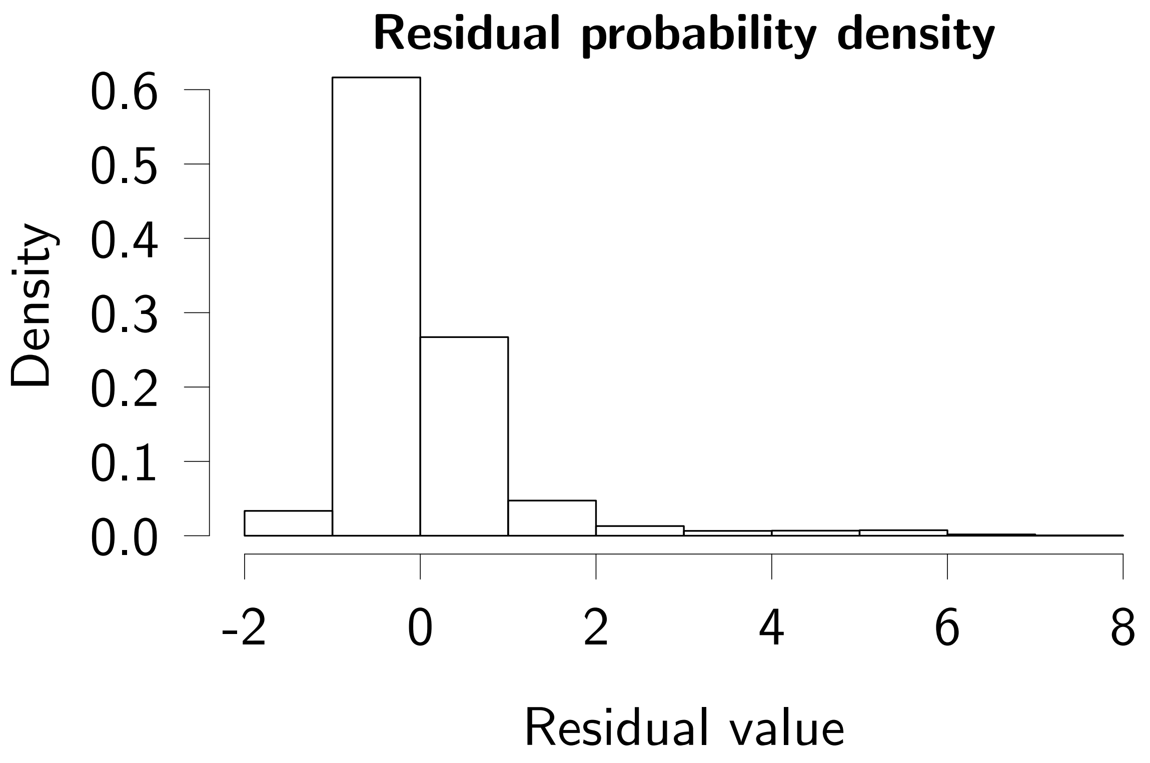

Figure 2: Residuals for the model on the natural scale

Taking the logarithm of the parturition date (previously filtered to be strictly positive) improves the fit quite a lot:

```
mm0log <- lmer(log(BirthDatePos)~1+OffspSex+(MumAge+MumAge2+MotherStatus)+
                 OffspBirthYear+
              (1|OffspBirthYear) + (1|MumId),
            data=birthdata, na.action = "na.omit")
shapiro.test(summary(mm0log)$residuals)

##
```

```
##  Shapiro-Wilk normality test
##
## data:  summary(mm0log)$residuals
## W = 0.9056, p-value < 2.2e-16
```

```
setPar()
hist(summary(mm0log)$residuals, freq = FALSE, xlab="Residual value",
     main="Residual probability density")
```

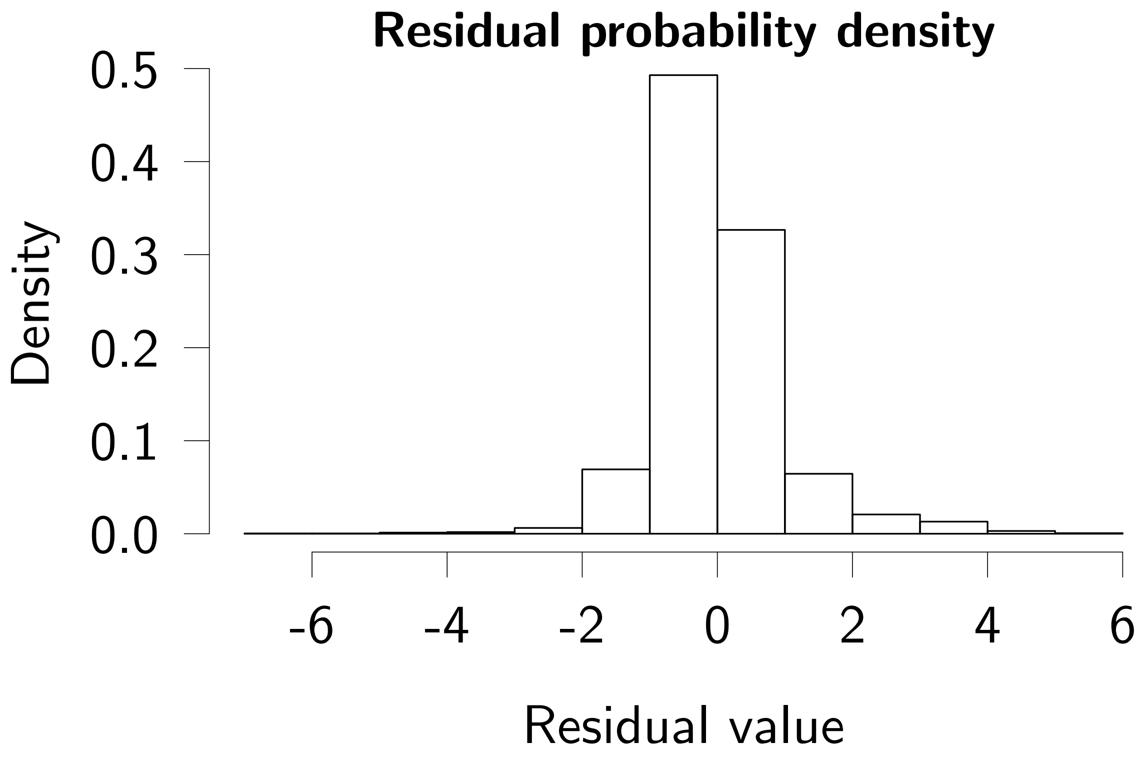

Figure 3: Residuals for the model with a log transform

5 Animal models

We fit a univariate animal model of transformed parturition date in order to estimate change in breeding values. The model includes as fixed effects:

- Inbreeding coefficient (pedigree-based)
- Sex of the offspring
- Mother status
  - Naive
  - True Yeld
  - Summer Yeld
  - Winter Yeld
  - Milk
- Mother age (first and second order)
- Genetic groups
- OPTIONALLY Temperature during the previous rut season
- OPTIONALLY calf birth year (continuous covariate)

and as random effects:

- Additive genetic variance
- Permanent environment variance
- Maternal variance (associated with the grand-mother of the new calf)
- Variance associated with the calf birth year
- Variance associated with the mother birth year
- (and of course residual variance)

Model with birth year as a fixed effect:

```
prior0_bdate <- list(G=list(G1=list(V=1, nu=0.002),
                            G2=list(V=1, nu=0.002),
                            G3=list(V=1, nu=0.002),
                            G4=list(V=1, nu=0.002),
                            G5=list(V=1, nu=0.002)),
                     R=list(V=1, nu=0.002))

mult <- 20
m0_bdate <- MCMCglmm(BirthDatePosLog100 ~ 1 + Inbreeding+ OffspSex + MotherStatus +
                       MumAge + MumAge2 + GGImm + OffspBirthYear,
              random=~ animal + MumPE + MotherMother + OffspBirthYear + MumYOB,
              rcov=~units,
              prior=prior0_bdate,
              data=birthdata,
              ginverse = list(animal=invAmatrix),
              nitt = 13000*mult, thin = 10*mult, burnin = 3000*mult,
              pr=TRUE)

#save(m0_bdate, file = "FilesToLoad/m0_bdateallg")
```

Note that we use the option pr=TRUE to save predicted breeding values for future analyses.
Model without birth year as a fixed effect:

```
prior0_bdate <- list(G=list(G1=list(V=1, nu=0.002),
                            G2=list(V=1, nu=0.002),
                            G3=list(V=1, nu=0.002),
                            G4=list(V=1, nu=0.002),
                            G5=list(V=1, nu=0.002)),
                     R=list(V=1, nu=0.002))

m0noC_bdate <- MCMCglmm(BirthDatePosLog100 ~ 1 + Inbreeding+ OffspSex + MotherStatus +
                       MumAge + MumAge2 + GGImm,
              random=~ animal + MumPE + MotherMother + OffspBirthYear + MumYOB,
              rcov=~units,
              prior=prior0_bdate,
              data=birthdata,
              ginverse = list(animal=invAmatrix),
              nitt = 130000, thin = 100, burnin = 30000,
              pr=TRUE, verbose = FALSE)
summary(m0noC_bdate)
save(m0noC_bdate, file = "FilesToLoad/m0noC_bdate")
```

5.0.1 Model with temperature

The addition of temperature in the animal model was suggested during peer-review, as a way to directly quantify phenotypic plasticity to temperature in our analyses instead of relying on estimates in different publications.

```
prior0_bdate <- list(G=list(G1=list(V=1, nu=0.002),
                            G2=list(V=1, nu=0.002),
                            G3=list(V=1, nu=0.002),
                            G4=list(V=1, nu=0.002),
                            G5=list(V=1, nu=0.002)),
                     R=list(V=1, nu=0.002))

m0_bdate_temp <- MCMCglmm(BirthDatePosLog100 ~ 1 + Inbreeding+ OffspSex + MotherStatus +
                       MumAge + MumAge2 + GGImm +temperature+ OffspBirthYear,
                     random=~ animal + MumPE + MotherMother + OffspBirthYear + MumYOB,
                     rcov=~units,
                     prior=prior0_bdate,
                     data=birthdata,
                     ginverse = list(animal=invAmatrix),
                     nitt = 26000, thin = 20, burnin = 6000,
                     pr=TRUE)
save(m0_bdate_temp, file = "FilesToLoad/m0_bdate_temp")
```

5.1 Heritability

```
#Uncorrected heritability
h2BDPL <- m0_bdate$VCV[,"animal"]/
              var(birthdata$BirthDatePosLog100[birthdata$MumGroup!=3], na.rm = TRUE)
HPDinterval(h2BDPL)

##          lower     upper
## var1 0.1072021 0.2063987
## attr(,"Probability")
## [1] 0.95

posterior.mode(h2BDPL)
```

```
##      var1
## 0.1661704

#Corrected heritability
corh2BDPL <- m0_bdate$VCV[,"animal"]/(m0_bdate$VCV[,"animal"]+
                                     m0_bdate$VCV[,"MumPE"]+
                                     m0_bdate$VCV[,"MotherMother"]+
                                     m0_bdate$VCV[,"MumYOB"] +
                                     m0_bdate$VCV[,"OffspBirthYear"]+
                                     m0_bdate$VCV[,"units"]+
      var(mean(m0_bdate$Sol[,"Inbreeding"])*birthdata$Inbreeding)+
      var(mean(m0_bdate$Sol[,"GGImm"])*birthdata$GGImm))

HPDinterval(corh2BDPL)

##         lower     upper
## var1 0.116232 0.2125237
## attr(,"Probability")
## [1] 0.95

posterior.mode(corh2BDPL)

##      var1
## 0.1671794

#repeatability
repBDPL <- (m0_bdate$VCV[,"animal"]+ m0_bdate$VCV[,"MumPE"]+
      m0_bdate$VCV[,"MotherMother"]+ m0_bdate$VCV[,"MumYOB"]+
      var(mean(m0_bdate$Sol[,"Inbreeding"])*birthdata$Inbreeding)+
var(mean(m0_bdate$Sol[,"GGImm"])*birthdata$GGImm)) /
                                    (m0_bdate$VCV[,"animal"]+
                                      m0_bdate$VCV[,"MumPE"]+
                                      m0_bdate$VCV[,"MotherMother"]+
                                      m0_bdate$VCV[,"OffspBirthYear"]+
                                      m0_bdate$VCV[,"MumYOB"] +
                                      m0_bdate$VCV[,"units"]+
     var(mean(m0_bdate$Sol[,"Inbreeding"])*birthdata$Inbreeding)+
var(mean(m0_bdate$Sol[,"GGImm"])*birthdata$GGImm))
posterior.mode(repBDPL)

##      var1
## 0.1787699

HPDinterval(repBDPL)

##          lower     upper
## var1 0.1423958 0.2200203
## attr(,"Probability")
## [1] 0.95
```

The mode of the posterior distribution for the heritability of parturition date is 0.157 (95% CI [0.107, 0.206]).

```
allranef <- list(m0_bdate$VCV[,"animal"],
      m0_bdate$VCV[,"MumPE"],
      m0_bdate$VCV[,"MotherMother"],
      m0_bdate$VCV[,"OffspBirthYear"],
      m0_bdate$VCV[,"MumYOB"],
      m0_bdate$VCV[,"units"])
```

```r
predm0_bdate <- predict(m0_bdate, marginal=m0_bdate$Random$formula,
                        posterior = "all")

tabranef <- data.frame(estimate=unlist(lapply(allranef, mean)),
            CI=unlist(lapply(allranef, function(x){
              paste0("[",round(HPDinterval(x)[1], 2), " ; ",
                     round(HPDinterval(x)[2], 2), "]")})),
            row.names = c("Additive genetic", "Permanent environment",
                          "Grand-mother identity", "Offspring birth year",
                          "Mother birth year", "Residuals"))
```

Table 1: Variance components

	estimate	CI
Additive genetic	219.614	[147.78 ; 284.53]
Permanent environment	9.747	[0 ; 54.31]
Grand-mother identity	1.630	[0 ; 10.04]
Offspring birth year	100.443	[46.61 ; 157.71]
Mother birth year	1.081	[0 ; 5.85]
Residuals	964.218	[916.42 ; 1017.1]

Visualize the prior:

```r
v <- 1
nu <- 0.002
n=100000
#same prior for all variance components
ra <- rIW(V = v*diag(1), nu = nu, n =  n)
re <- rIW(V = v*diag(1), nu = nu, n =  n)
rm <- rIW(V = v*diag(1), nu = nu, n =  n)
rpe <- rIW(V = v*diag(1), nu = nu, n =  n)
ry <- rIW(V = v*diag(1), nu = nu, n =  n)
rby <- rIW(V = v*diag(1), nu = nu, n =  n)

rh2 <- ra/(ra+re+rm+rpe+ry+rby)
rh2 <- rh2[!is.nan(rh2)] #sad but necessary trick?
```

```r
setPar()

plot(density(rh2, from =0, to=1, adjust=1), xlim=c(0,1),
     xlab="heritability", main="")
lines(density(h2BDPL), col="red")
legend(x = "topright", col=c("black", "red"),
       legend = c("Prior", "Posterior"), lty=1)
```

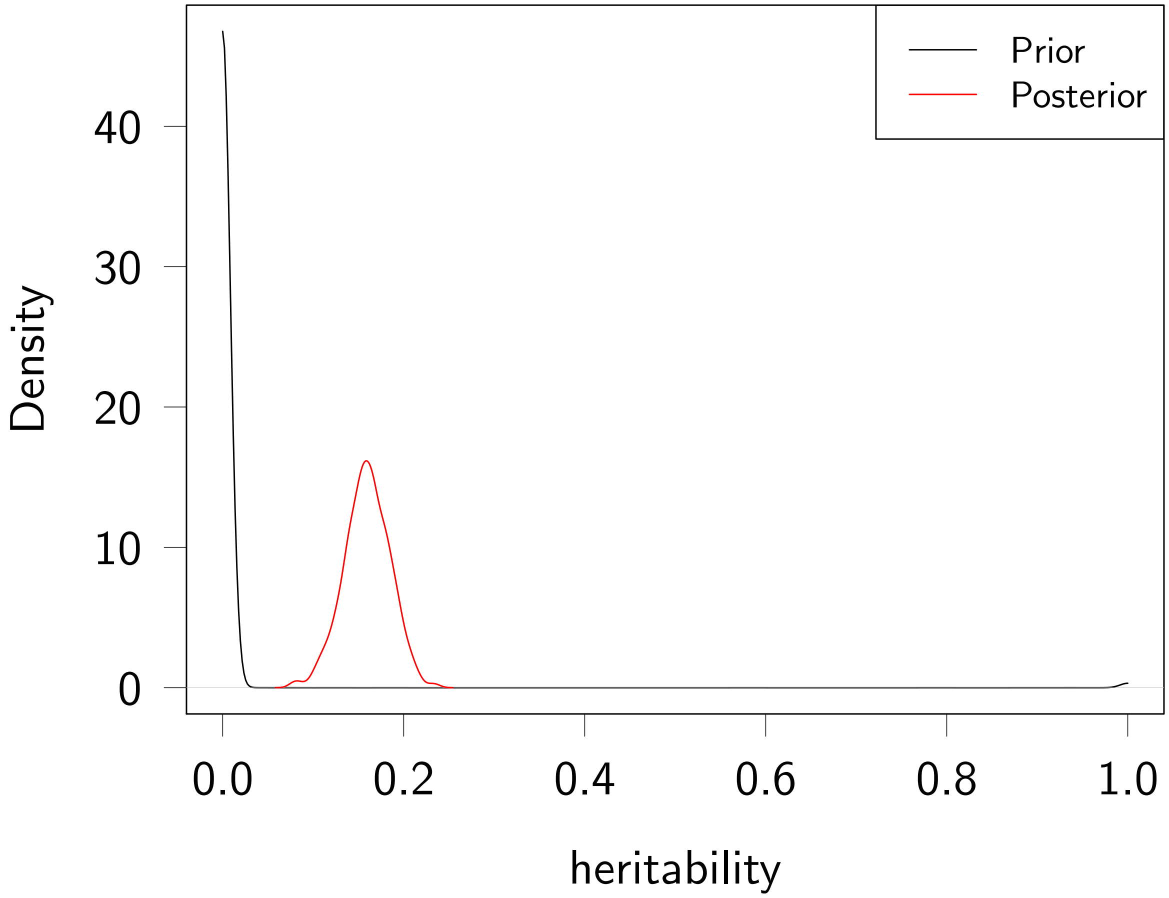

Figure 4: Prior and posterior distribution for heritability of parturition date

5.2 Trends in breeding values

Extracting BLUPs for breeding values:

```
BV<-m0_bdate$Sol[,grep(pattern = "animal*",x = colnames(m0_bdate$Sol))]
namesBV<- substr(dimnames(BV)[[2]],start = 8,
                 stop = nchar(dimnames(BV)[[2]]))
BV<-BV[,which(namesBV %in% birthdata$animal)]

animalID<-substr(x = colnames(BV),start = 8,
                 stop=nchar(colnames(BV)))
pmBV<-data.frame(animalID,posterior.mode(BV))
names(pmBV)<-c("animal","pBV")

meanY<-data.frame(tapply(birthdata$OffspBirthYear,birthdata$animal,mean),
          names(tapply(birthdata$OffspBirthYear,birthdata$animal,mean)))
names(meanY)<-c("year","animal")

mpmBV<-merge(x = pmBV,y = meanY,by = "animal",all.x=TRUE ,
             all.y = FALSE, sort=FALSE)
```

Now doing a linear regression on the full posterior distribution.

```
setPar()

lmBV<-as.mcmc(apply(BV,MARGIN = 1,
                    function(x){coef(lm(x~1+mpmBV$year))[2]}))
save(lmBV, file = "FilesToLoad/lmBV")
```

Same thing but with the total predicted change over the study period, that is, from 1972 to 2016.

```
posterior.mode(lmBV*(max(birthdata$OffspBirthYear)-min(birthdata$OffspBirthYear)))

##     var1
## -3.65628

HPDinterval(lmBV*(max(birthdata$OffspBirthYear)-min(birthdata$OffspBirthYear)))

##          lower    upper
## var1 -10.02551 1.461519
## attr(,"Probability")
## [1] 0.95
```

Creating a splined posterior distribution of the trend in breeding values (in order to visualize potential non-linear changes).

```
mmy<-cbind(1, min(birthdata$OffspBirthYear):max(birthdata$OffspBirthYear))
lmBVintsl<-apply(BV,MARGIN = 1,
                 function(x){coef(lm(x~1+mpmBV$year))})
pred <- mmy %*% lmBVintsl

predmode <- apply(X = pred, MARGIN = 1,
                  function(x){mean(as.mcmc(x))})

predlow <- apply(X = pred, MARGIN = 1,
                 function(x){quantile(as.mcmc(x),
                                      probs = 0.025)})
predhigh <- apply(X = pred, MARGIN = 1,
                  function(x){quantile(as.mcmc(x),
                                      probs = 0.975)})
```

5.3 Conversion back to days

We have modeled $z = 100\log(B)$, where B is the parturition date in number of days after May 1st. What was the effect of evolution (written d on the scale of z) on the scale of B?

$$\begin{aligned}\Delta B =& \bar{B_1} - \bar{B_0} \\ =& \exp(\frac{z_1}{100} + \sigma^2(y/100)/2) - \exp(\frac{z_0}{100} + \sigma^2(y/100)/2) \\ =& \exp(\frac{z_0 + d}{100} + \sigma^2(y/100)/2) - \exp(\frac{z_0}{100} + \sigma^2(y/100)/2)\end{aligned}$$

Let's try with phenotypic change only, to confirm the back-transformation gives a number of days consistent with the direct linear estimate.

```
funtrans <- function(x)
{
  exp((x+initialmean)/100+vardat/2) - exp((initialmean)/100+vardat/2)
}
```

Application

```
  mbdlog <- lm(BirthDatePosLog100~1+OffspBirthYear,
            data=birthdata, na.action = "na.omit")

nbyears <- 2016-1972
  smbdlog <- summary(mbdlog)

  changePheno <- coefficients(smbdlog)[2,1] *( nbyears)

  initialmean <- mean(predict(mbdlog)[birthdata$OffspBirthYear==1972])
  vardat <- sigma(mbdlog)^2 / 10000

#back transformed estimate
  funtrans(changePheno)

## [1] -13.42614

  funtrans(changePheno-1.96*smbdlog$coefficients[2,2]*( nbyears))

## [1] -14.95485

  funtrans(changePheno+1.96*smbdlog$coefficients[2,2]*( nbyears))

## [1] -11.82453

#linear estimate with confidence interval
     mbd <-lm(BirthDatePos~1+OffspBirthYear,
            data=birthdata, na.action = "na.omit")
  smbd <- summary(mbd)
  smbd$coefficients[2,1]*( nbyears)

## [1] -12.31803

  (smbd$coefficients[2,1]-1.96*smbd$coefficients[2,2])*(nbyears)

## [1] -14.53171

  (smbd$coefficients[2,1]+1.96*smbd$coefficients[2,2])*(nbyears)

## [1] -10.10436
```

Now with change in BLUPs for breeding values.

```
changeBV <- lmBV*(max(birthdata$OffspBirthYear)-min(birthdata$OffspBirthYear))
daychanges <- funtrans(changeBV)

posterior.mode(daychanges, adjust = 2)

##      var1
## -2.107718

HPDinterval(daychanges)

##          lower     upper
## var1 -4.484442 0.6921056
## attr(,"Probability")
## [1] 0.95

mean(daychanges>0)

## [1] 0.077
```

In different units:

```
#change per year
posterior.mode(daychanges)/44

##        var1
## -0.03820073

#change per generation
8*posterior.mode(daychanges)/44

##       var1
## -0.3056058

#haldanes
8*(posterior.mode(daychanges)/
     sd(birthdata$BirthDatePos[birthdata$OffspBirthYear<1973],
                                 na.rm=TRUE))/44

##        var1
## -0.02380512
```

We need the variance on the log-transformed trait to predict accurately the means on the data scale

```
#assuming constant variance and removing the factor 100

predmodeday <- funtrans(predmode)
predlowday <- funtrans(predlow)
predhighday <- funtrans(predhigh)

setPar()
load(file = "FilesToLoad/bvplotlist")
plot(x=0,xlim=c(min(birthdata$OffspBirthYear),max(birthdata$OffspBirthYear)),
     ylim=c(-5,3),type="n", xlab="Year",
     ylab="Mean breeding value (days)")
trashidontwantyou<-lapply(bvplotlist,
          function(x){lines(x[,1], funtrans(x[,2])-1.22,
                      col=rgb(0.1,0.1,0.1,alpha = 0.1))})
abline(h=0)



redtrans <- rgb(1,0,0,0.7)
lines(x=mmy[-1,2], predmodeday[-1]-predmodeday[1], type="l", col=redtrans, lwd=8)
lines(x=mmy[-1,2],predlowday[-1]-predmodeday[1], col=redtrans, lty=2, lwd=5)
lines(x=mmy[-1,2],predhighday[-1]-predmodeday[1], col=redtrans, lty=2, lwd=5)
```

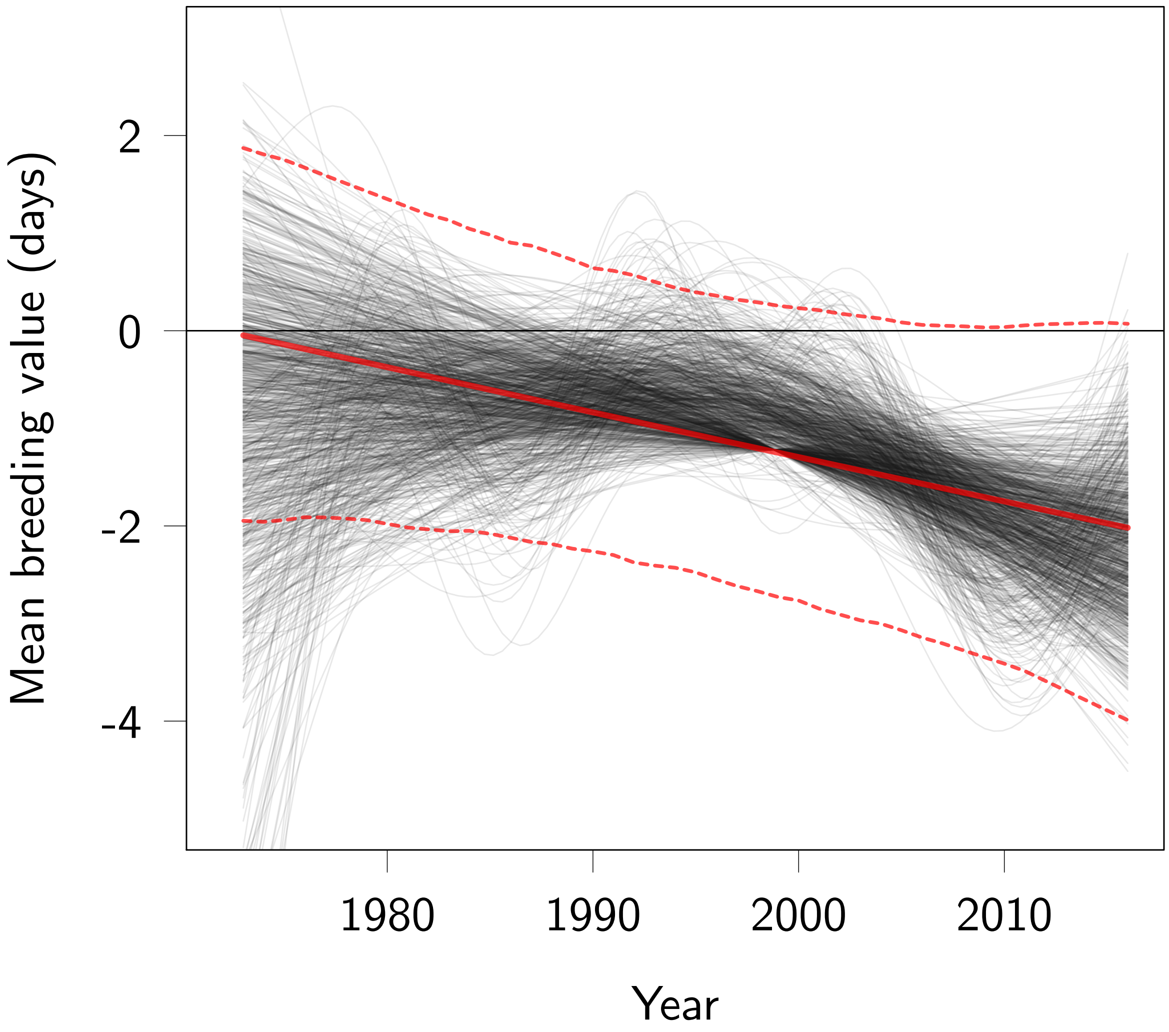

Figure 5: Posterior distribution of the change in breeding values for parturition dates. The black lines represent the posterior distribution of a smoother (additive model).

5.4 Less conservative estimate of evolution

We can rerun the previous analysis with an animal model that does not contain year as a fixed effect. In that case the estimated trend in breeding values is likely over-estimated (while it was under-estimated previously).

```
BVanti <- m0noC_bdate$Sol[,grep(pattern = "animal*",
                                x = colnames(m0noC_bdate$Sol))]
namesBVanti<- substr(dimnames(BVanti)[[2]],start = 8,
                 stop = nchar(dimnames(BVanti)[[2]]))
BVanti<-BVanti[,which(namesBVanti %in% birthdata$animal)]

pmBVanti<-data.frame(animalID,posterior.mode(BVanti))
names(pmBVanti)<-c("animal","pBV")

mpmBVanti<-merge(x = pmBVanti,y = meanY,by = "animal",all.x=TRUE ,
                 all.y = FALSE, sort=FALSE)

lmBVanti<-as.mcmc(apply(BVanti,MARGIN = 1,
                    function(x){coef(lm(x~1+mpmBVanti$year))[2]}))
HPDinterval(lmBVanti)

##             lower        upper
## var1 -0.2849693 -0.001247471
## attr(,"Probability")
## [1] 0.95

posterior.mode(lmBVanti)

##        var1
## -0.1384326

mean(lmBVanti>0)

## [1] 0.021

changeBVanti <- lmBVanti*(max(birthdata$OffspBirthYear)-
                            min(birthdata$OffspBirthYear))
daychangesanti <- funtrans(changeBVanti)

posterior.mode(daychangesanti)

##       var1
## -2.785572

HPDinterval(daychangesanti)

##            lower       upper
## var1 -5.539852 -0.02579611
## attr(,"Probability")
## [1] 0.95
```

5.5 Drift

What could have been the effect of genetic drift over this period? (note that we did not provide the pedigree to run the following)

```
BVdtemp <- t(sapply(X = as.numeric(m0_bdate$VCV[,"animal"]),
                    FUN = function(x) {
  rbv(pedigree = deerped,G = x)} ))
```

```r
colnames(BVdtemp) <- deerped[,1]

lmBVdrift<-as.mcmc(unlist(apply(X =BVdtemp, MARGIN = 1,
                                FUN = function(x){
  prbv0<-data.frame(animal=names(x),b=x)
  mpmBVdrift<-merge(x = prbv0,y = birthdata[,c("animal", "OffspBirthYear")],
                    by = "animal",all.x=FALSE,all.y = TRUE)
  return(
    coef(lm(mpmBVdrift[,"b"]~1+mpmBVdrift$OffspBirthYear))[2]
    )
  }
  )
  )
  )
#save(lmBVdrift, file = "FilesToLoad/lmBVdrift")
```

Distribution of the simulated changes due to drift

```r
load("FilesToLoad/lmBVdrift")
setPar()

dayslmBVdrift <- funtrans(lmBVdrift*(max(birthdata$OffspBirthYear)-
                            min(birthdata$OffspBirthYear)))
plot(dayslmBVdrift)
```

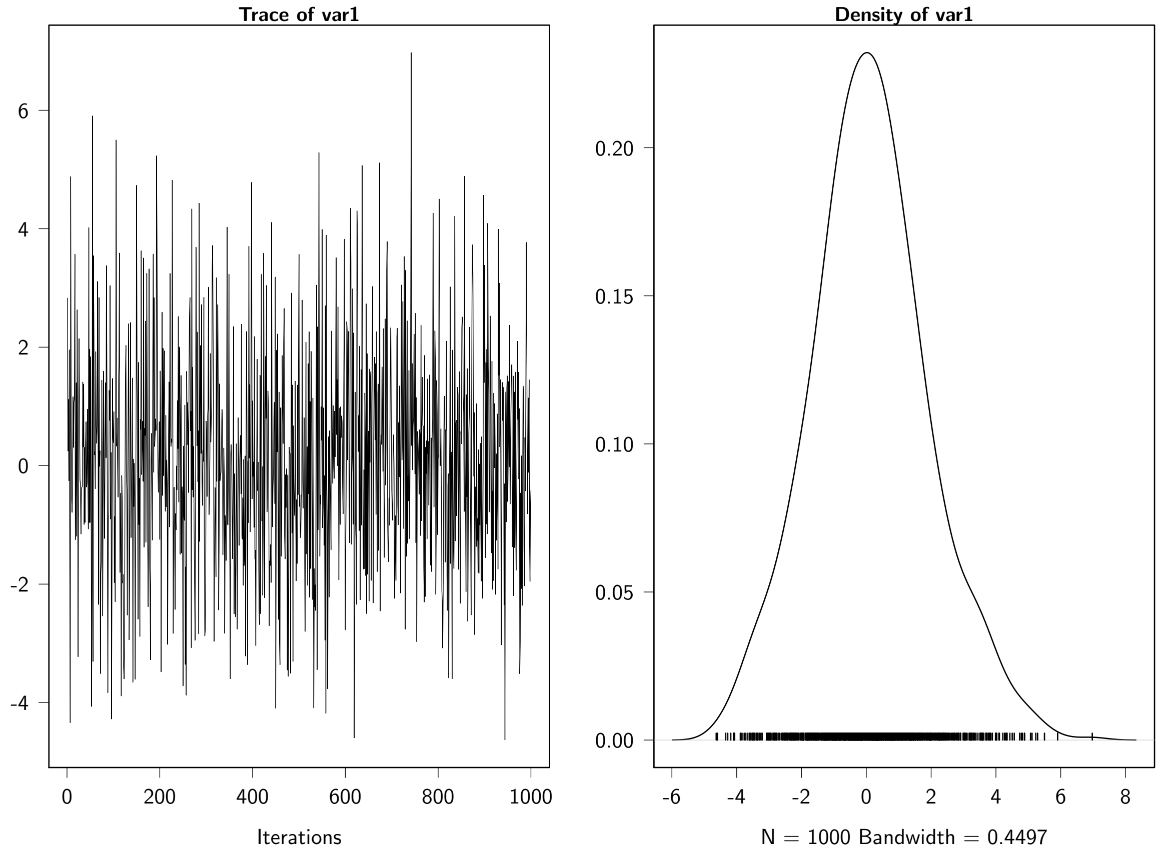

Frequency with which drift produces more extreme evolution than the best estimate (posterior mode) of evolution.

```r
mean(lmBVdrift<=posterior.mode(lmBV, adjust = 2))

## [1] 0.109
```

5.6 Changes in non-genetic components of parturition date

Other components of the models appear to explain a modest amount of change.

5.6.1 Age

Mother age changed quite a lot during the study period.

```
setPar()
plot(tapply(birthdata$MumAge, birthdata$OffspBirthYear, mean),
     main="", xlab="Year", ylab="Mean mother age")
```

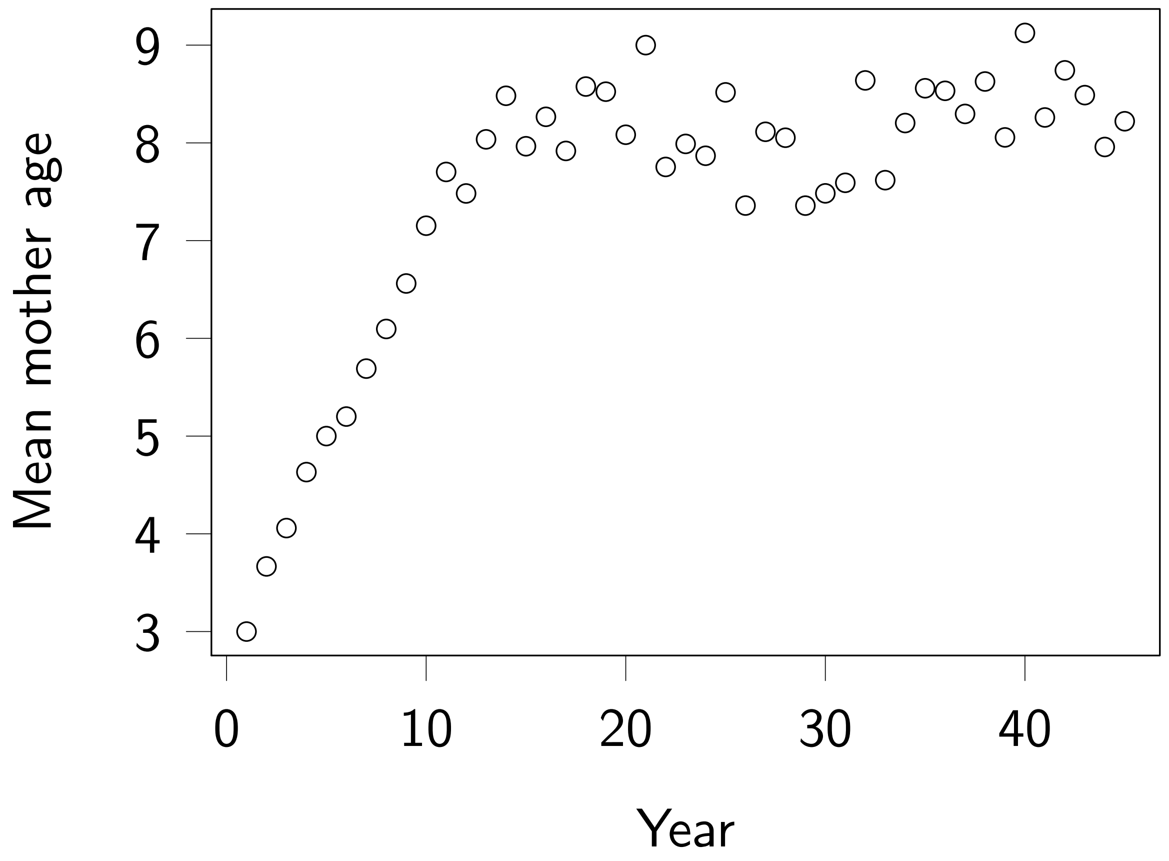

However, first and second order effects of age have opposite effects that mostly cancel each other out though.

```
dmage <- data.frame(int=1, age=tapply(birthdata$MumAge,
                                      birthdata$OffspBirthYear, mean),
                age2 = tapply(birthdata$MumAge2, birthdata$OffspBirthYear, mean))

predage<-as.matrix(dmage) %*% c(0, mean(m0_bdate$Sol[,"MumAge"]),
                                mean(m0_bdate$Sol[,"MumAge2"]))
predagemat <- matrix(data = 0, nrow = 1000, ncol = 45)
for (i in 1:1000)
{
  predagemat[i,] <- as.matrix(dmage) %*% c(0, (m0_bdate$Sol[i,"MumAge"]),
                          (m0_bdate$Sol[i,"MumAge2"]))
}
predagematday <- exp((predagemat+initialmean)/100+vardat/2) -
                    exp(initialmean/100+vardat/2)
```

```r
setPar()
plot(x=1971+1:45, y=1:45, type="n", ylim=c(-40,-8), ylab="Predicted effect",
     xlab = "Year", main="Age structure")
for (i in 1:45)
  {
  points(x = i+1971, y=posterior.mode(as.mcmc(predagemat[,i])), pch=16)
  arrows(x0 = i+1971,y0=HPDinterval(as.mcmc(predagemat[,i]))[1],
         y1=HPDinterval(as.mcmc(predagemat[,i]))[2], angle = 90,
         code = 3, length = 0.1)
}
```

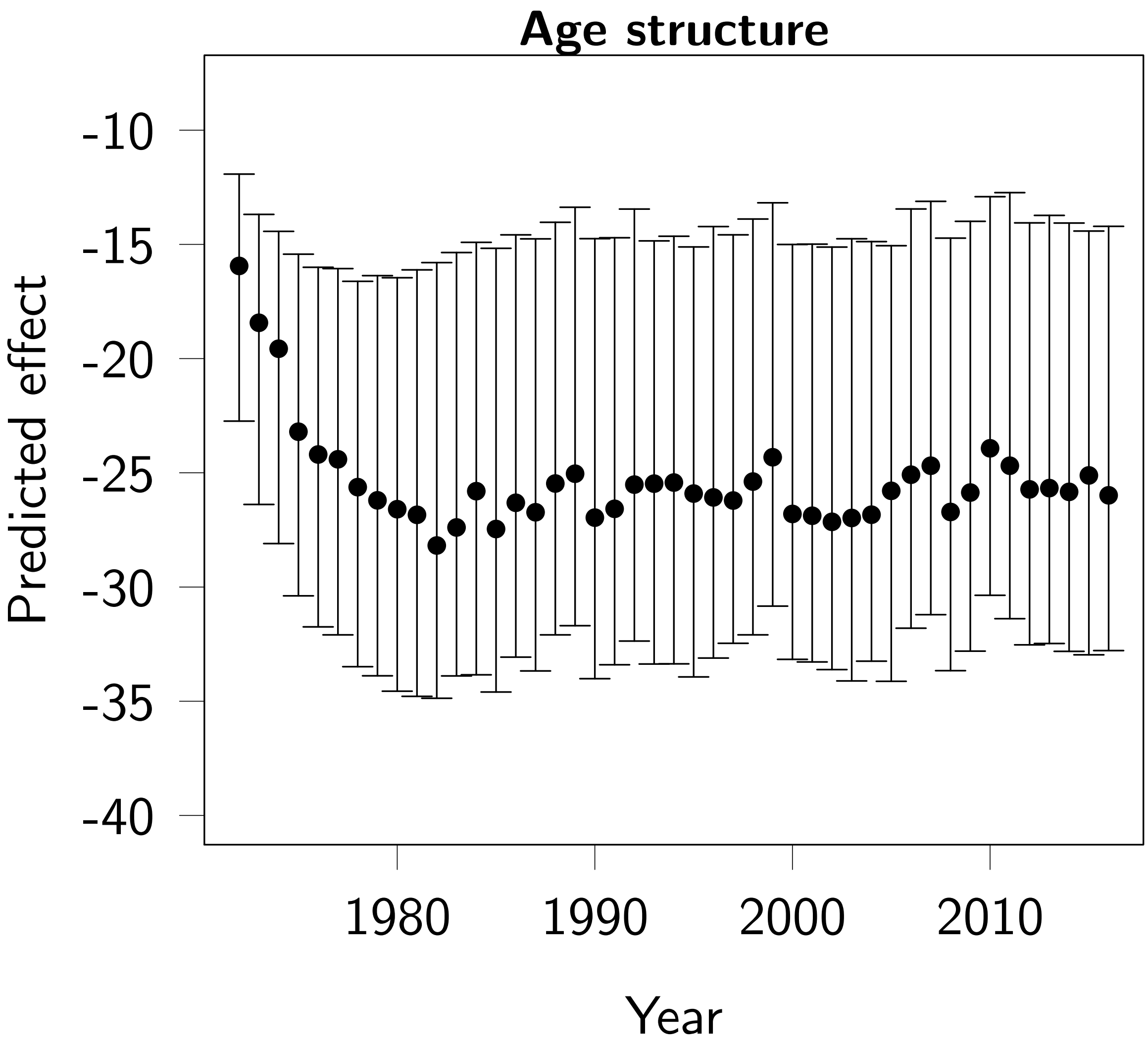


```r
ty1 <- 1:10
p1mstatlm <- apply(predagemat[,1:10], MARGIN = 1,
                   function(x){exp((initialmean+(coef(lm(x~ty1))[2]*10))/100 + vardat/2) -
    exp((initialmean)/100 + vardat/2)})
HPDinterval(as.mcmc(p1mstatlm))

##          lower     upper
## var1 -5.619088 -2.144162
## attr(,"Probability")
## [1] 0.95

posterior.mode(as.mcmc(p1mstatlm))

##      var1
```

```
## -3.824867

ty2 <- 1:36
p2mstatlm <- apply(predagemat[,10:45], MARGIN = 1,
                   function(x){exp((initialmean+(coef(lm(x~ty2))[2]*36))/100 + vardat/2) -
    exp((initialmean)/100 + vardat/2)})
HPDinterval(as.mcmc(p2mstatlm))

##          lower     upper
## var1 0.6267473 0.9285056
## attr(,"Probability")
## [1] 0.95

posterior.mode(as.mcmc(p2mstatlm))

##      var1
## 0.7883755

ty <- 1:45
magelmrate <- apply(predagemat, MARGIN = 1,
                 function(x){coef(lm(x~ty))[2]})
magelm <- funtrans(magelmrate*44)
posterior.mode(as.mcmc(magelm))

##       var1
## -0.3332399

HPDinterval(as.mcmc(magelm))

##          lower     upper
## var1 -1.118457 0.7281659
## attr(,"Probability")
## [1] 0.95
```

5.6.2 Reproductive status

The reproductive status of mother changed a lot at the beginning of the monitoring, and then stabilized:

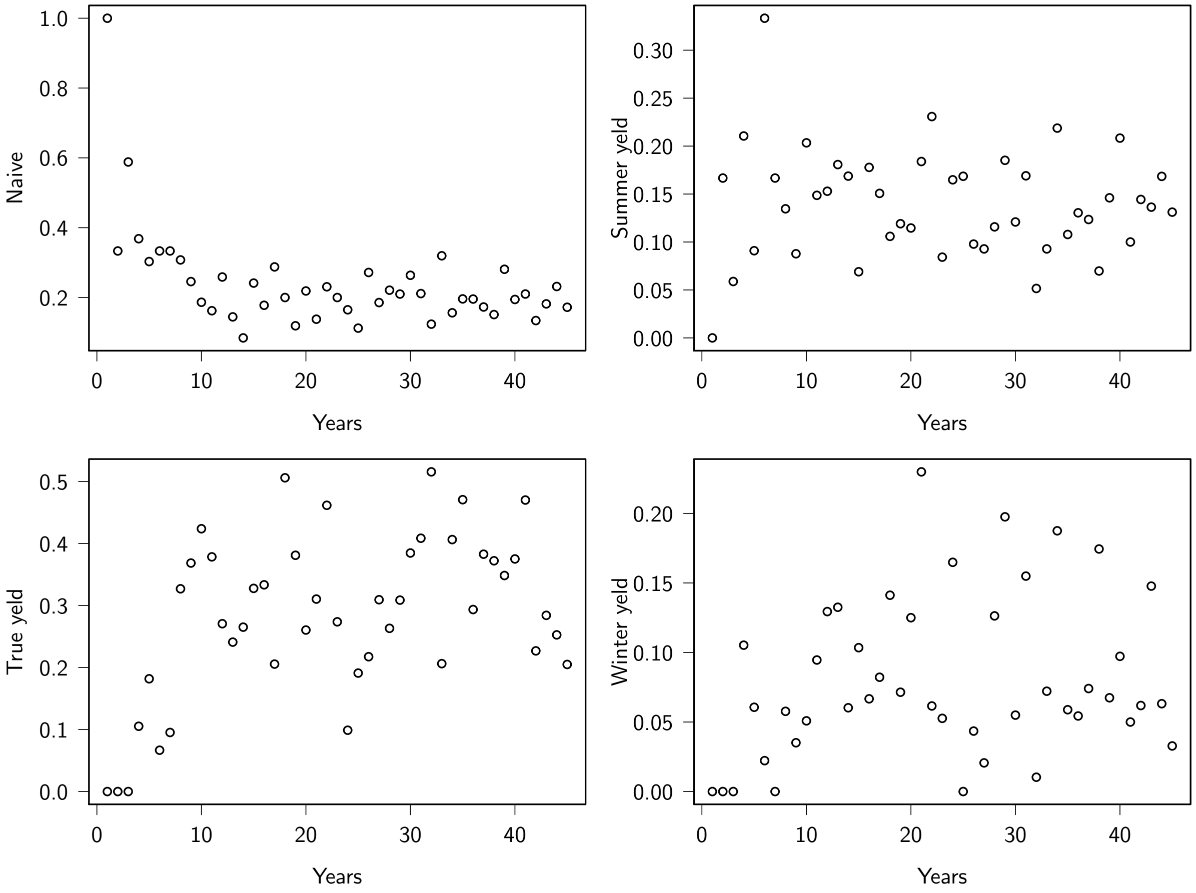

This does not seem to have had a significant effect on the mean timing of reproduction however:

```
dmmstat <- data.frame(int=1,
                      naive=tapply(birthdata$MotherStatus=="Naive",
                                   birthdata$OffspBirthYear, mean),
                      summery=tapply(birthdata$MotherStatus=="Summeryeld",
                                     birthdata$OffspBirthYear, mean),
                      truey=tapply(birthdata$MotherStatus=="Trueyeld",
                                   birthdata$OffspBirthYear, mean),
                      wintery=tapply(birthdata$MotherStatus=="Winteryeld",
                                     birthdata$OffspBirthYear, mean))



predmstatmat <- matrix(data = 0, nrow = 1000, ncol = 45)
for (i in 1:1000)
{
  predmstatmat[i,] <- as.matrix(dmmstat) %*% c(0, (m0_bdate$Sol[i,"MotherStatusNaive"]),
                         (m0_bdate$Sol[i,"MotherStatusSummeryeld"]),
                         (m0_bdate$Sol[i,"MotherStatusTrueyeld"]),
                         (m0_bdate$Sol[i,"MotherStatusWinteryeld"]))
}

predmstatmatday <- exp((predmstatmat+initialmean)/100+vardat/2) -
                    exp(initialmean/100+vardat/2)

ty <- 1:45
```

```
mmatlmrate <- apply(predmstatmat, MARGIN = 1,
                function(x){(coef(lm(x~ty))[2])})
mmatlm <- funtrans(mmatlmrate*44)
posterior.mode(as.mcmc(mmatlm))

##       var1
## -0.4553816

HPDinterval(as.mcmc(mmatlm))

##           lower      upper
## var1 -0.9448141 0.01214306
## attr(,"Probability")
## [1] 0.95
```

```
setPar()
plot(x=1971+1:45, y=1:45, type="n", ylim=c(-9,-2), ylab="Predicted effect",
     xlab = "Year", main="Female reproductive status")
for (i in 1:45)
  {
  points(x = i+1971, y=posterior.mode(as.mcmc(predmstatmatday[,i])), pch=16)
  arrows(x0 = i+1971,y0=HPDinterval(as.mcmc(predmstatmatday[,i]))[1],
         y1=HPDinterval(as.mcmc(predmstatmatday[,i]))[2], angle = 90,
         code = 3, length = 0.1)
}
```

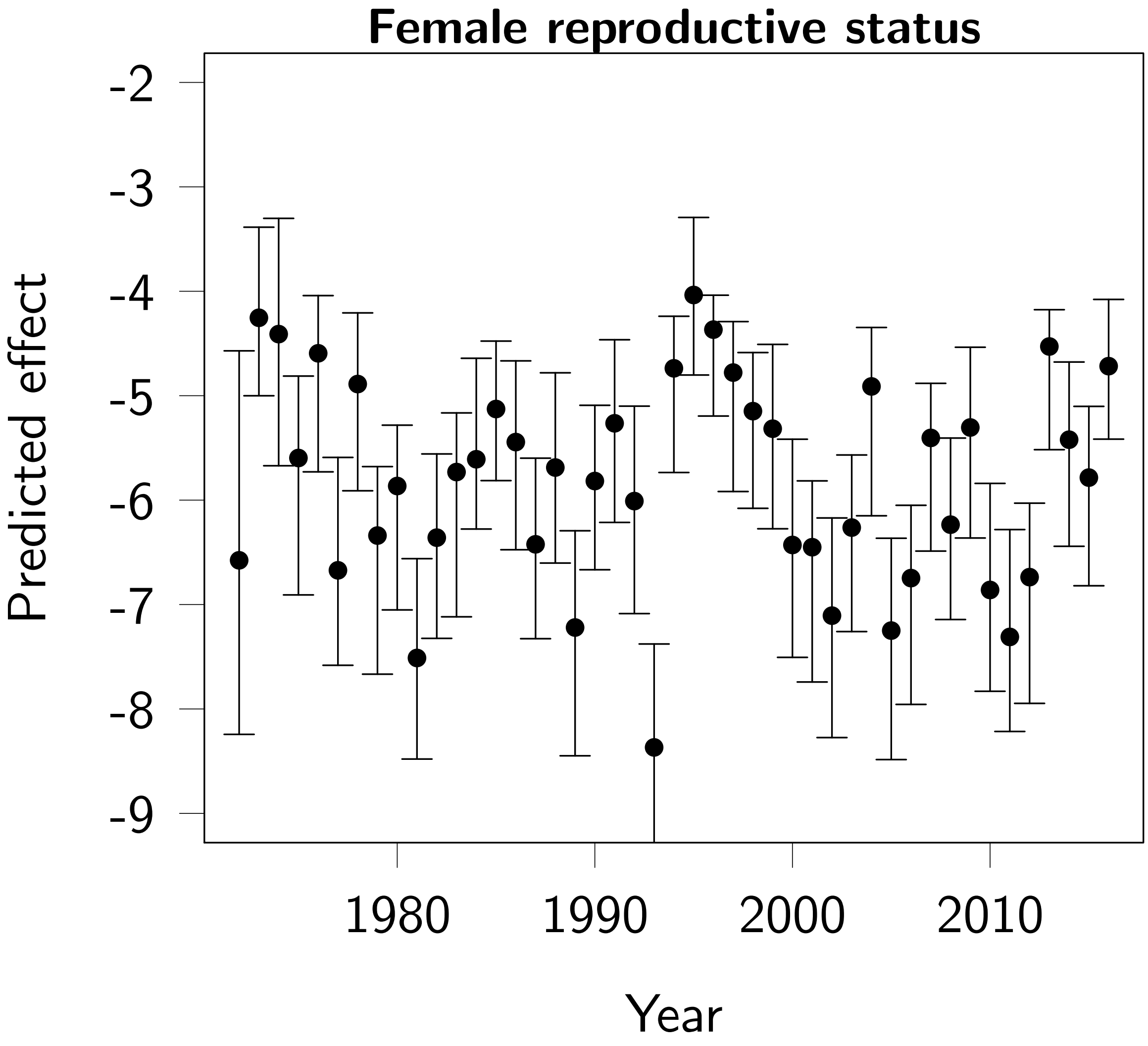

Age and reproductive status together:

```
par(mfrow=c(1,2))
setPar()
plot(x=1971+1:45, y=1:45, type="n", ylim=c(-8,3), ylab="Predicted effect (days)",
     xlab = "Year", main="")
for (i in 1:45)
  {
  points(x = i+1971, y=posterior.mode(as.mcmc(predagematday[,i]))-
           posterior.mode(as.mcmc(predagematday[,1])), pch=16)
  arrows(x0 = i+1971,y0=HPDinterval(as.mcmc(predagematday[,i]))[1]-
           posterior.mode(as.mcmc(predagematday[,1])),
         y1=HPDinterval(as.mcmc(predagematday[,i]))[2]-
           posterior.mode(as.mcmc(predagematday[,1])), angle = 90,
         code = 3, length = 0.1)
}
lines(x=1972:1981, y= (1972:1981-1971)*posterior.mode(as.mcmc(p1mstatlm/10))-0.5,
      col="red", lty=3, lwd=4)
lines(x=1981:2016, y= (1981:2016-1981)*posterior.mode(as.mcmc(p2mstatlm/36))+
        mean(posterior.mode(as.mcmc(predagematday[,11:45]))-
           posterior.mode(as.mcmc(predagematday[,1])))-0.2,
      col="red", lty=3, lwd=4)
lines(x=1972:2016, y= (1972:2016-1981)*posterior.mode(as.mcmc(magelm/44))+
        mean(posterior.mode(as.mcmc(predagematday[,1:45]))-
           posterior.mode(as.mcmc(predagematday[,1]))),
```

```
      col="red", lty=2, lwd=7)


text(x = 2012, y=2.5, labels = "\\textbf{(A)}")

setPar()
plot(x=1971+1:45, y=1:45, type="n", ylim=c(-3.2,3.2),
     ylab="Predicted effect (days)", xlab = "Year", main="")
for (i in 1:45)
  {
  points(x = i+1971, y=posterior.mode(as.mcmc(predmstatmatday[,i]))-
           posterior.mode(as.mcmc(predmstatmatday[,1])), pch=16)
  arrows(x0 = i+1971,y0=HPDinterval(as.mcmc(predmstatmatday[,i]))[1]-
           posterior.mode(as.mcmc(predmstatmatday[,1])),
         y1=HPDinterval(as.mcmc(predmstatmatday[,i]))[2]-
           posterior.mode(as.mcmc(predmstatmatday[,1])), angle = 90,
         code = 3, length = 0.1)
}

lines(x=1972:2016, y= (1972:2016-1971)*posterior.mode(as.mcmc(mmatlm/44)) +
        mean(posterior.mode(as.mcmc(predmstatmatday[,1:45]))-
           posterior.mode(as.mcmc(predmstatmatday[,1]))), col="red", lty=2, lwd=7)
text(x = 2012, y=2.7, labels = "\\textbf{(B)}")
```

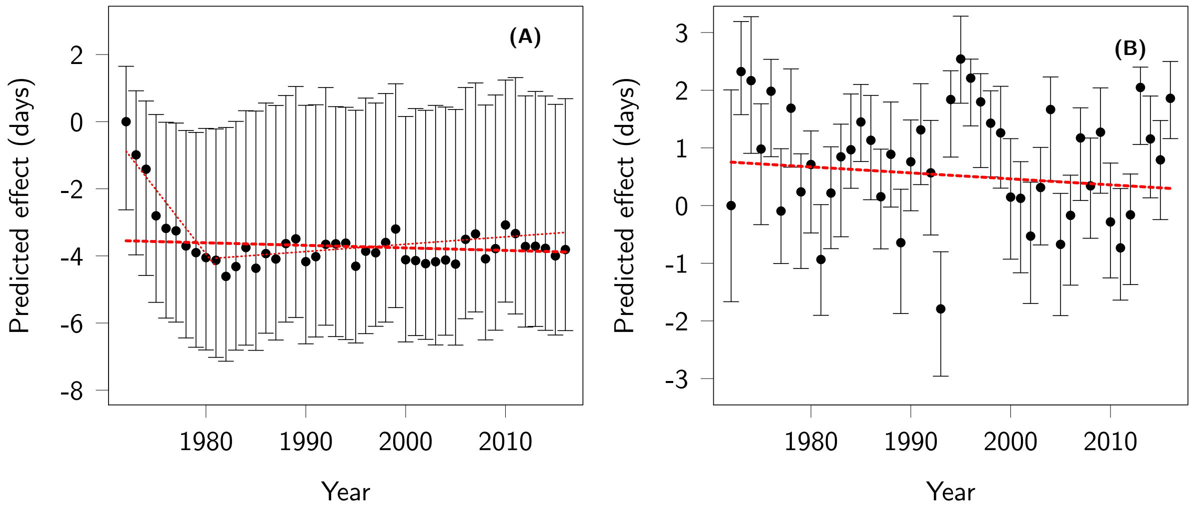


```
par(mfrow=c(1,1))
```

5.6.3 Offspring sex

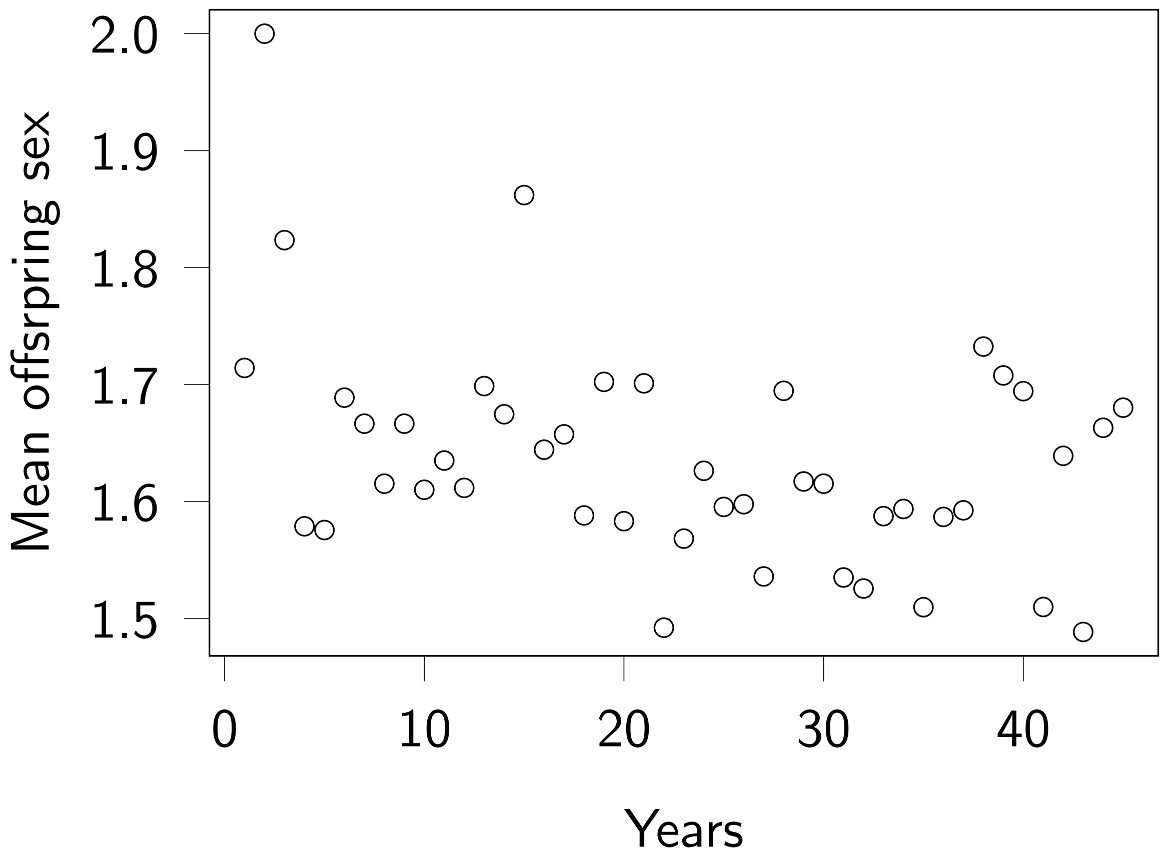


```
predsexoff <- outer(m0_bdate$Sol[,"OffspSex"],
      tapply(birthdata$OffspSex, birthdata$OffspBirthYear, mean))
sexlogchangerate <- apply(predsexoff, MARGIN = 1, function(x){
  coefficients(lm(x~I(1:45)))[2]})
sexlogchange <- sexlogchangerate*44
sexchange <- funtrans(sexlogchange)

posterior.mode(as.mcmc(sexchange))

##      var1
## -0.123735

HPDinterval(as.mcmc(sexchange))

##           lower       upper
## var1 -0.2223293 -0.01765799
## attr(,"Probability")
## [1] 0.95
```

5.6.4 Inbreeding

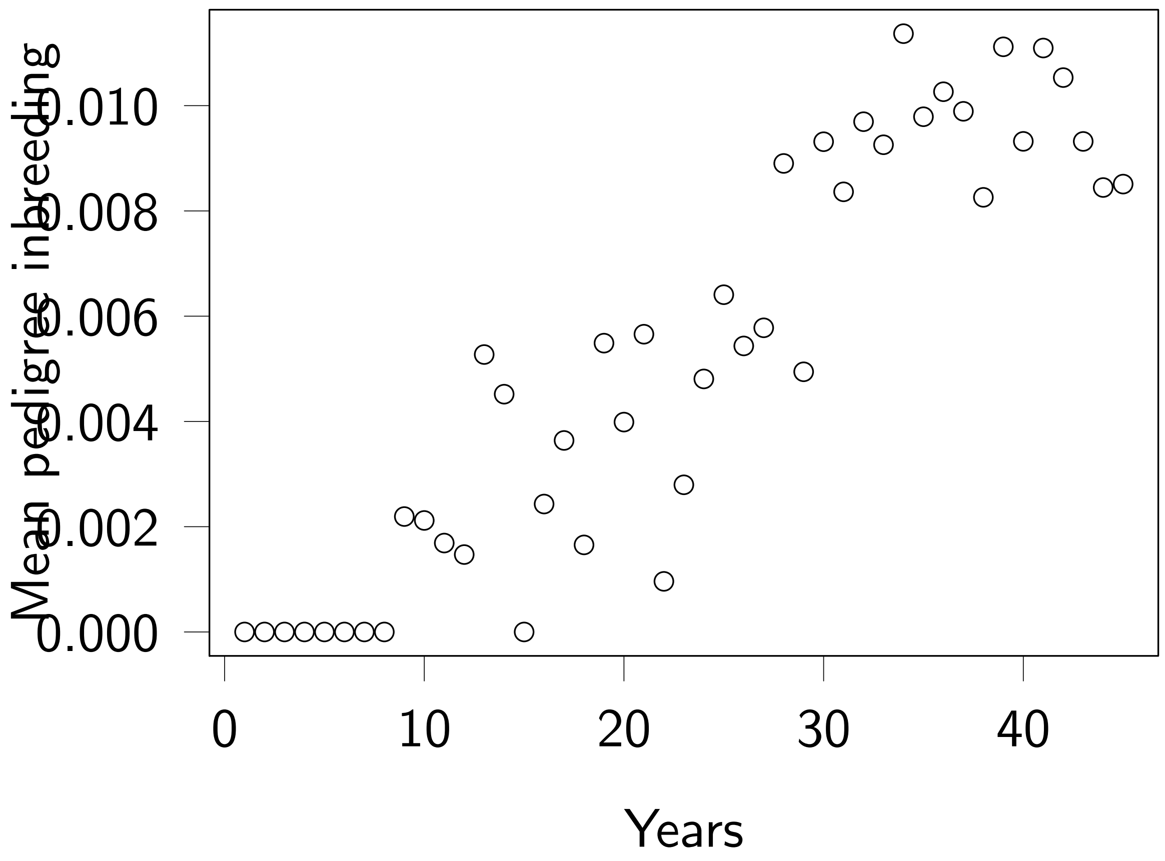


```
predinb <- outer(m0_bdate$Sol[,"Inbreeding"],
      tapply(birthdata$Inbreeding, birthdata$OffspBirthYear, mean))
inblogchangerate <- apply(predinb, MARGIN = 1, function(x){
  coefficients(lm(x~I(1:45)))[2]})
inbchange <- funtrans(inblogchangerate*44)

posterior.mode(as.mcmc(inbchange))

##      var1
## 0.2500602

HPDinterval(as.mcmc(inbchange))

##           lower     upper
## var1 -0.3196024 0.6593986
## attr(,"Probability")
## [1] 0.95
```

5.6.5 Immigrant origin

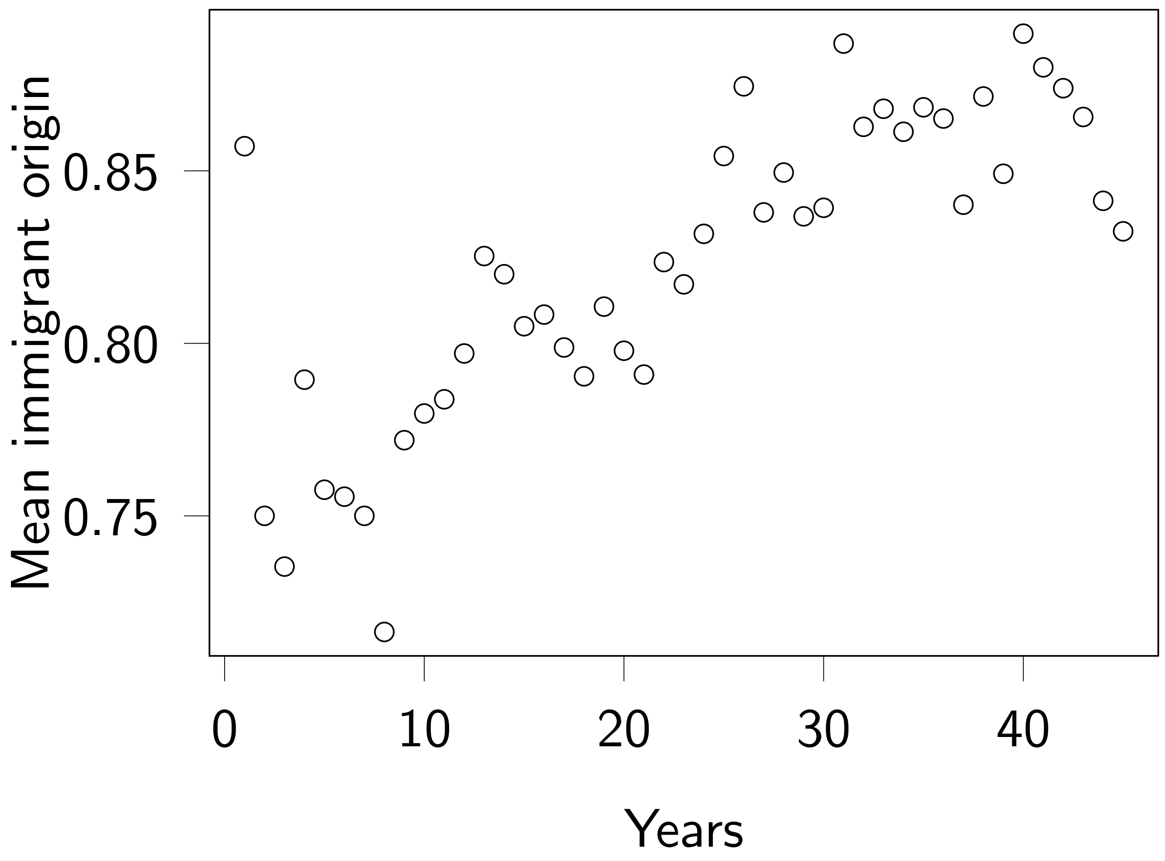


```
predgg <- outer(m0_bdate$Sol[,"GGImm"],
      tapply(birthdata$GGImm, birthdata$OffspBirthYear, mean))
gglogchangerate <- apply(predgg, MARGIN = 1, function(x){
  coefficients(lm(x~I(1:45)))[2]})
ggchange <- funtrans(gglogchangerate*44)

posterior.mode(as.mcmc(ggchange))

##      var1
## 0.2388147

HPDinterval(as.mcmc(ggchange))

##           lower    upper
## var1 -0.3357979 0.755653
## attr(,"Probability")
## [1] 0.95
```

5.7 Temperature

```
load("FilesToLoad/m0_bdate_temp")


dmtemp <- data.frame(int=1, age=tapply(birthdata$temperature,
                                       birthdata$OffspBirthYear, mean))

predtempmat <- matrix(data = 0, nrow = 1000, ncol = 45)
```

```
for (i in 1:1000)
{
  predtempmat[i,] <- as.matrix(dmtemp) %*% c(0, (m0_bdate_temp$Sol[i,"temperature"]))
}
predtempday <- funtrans(predtempmat)

ty <- 1:45
tempeffrate <- apply(predtempmat, MARGIN = 1,
                  function(x){coef(lm(x~ty))[2]})
tempeff <- funtrans(tempeffrate*44)
HPDinterval(as.mcmc(tempeff))

##          lower     upper
## var1 -2.987927 0.4928039
## attr(,"Probability")
## [1] 0.95

posterior.mode(as.mcmc(tempeff), adjust = 10)

##      var1
## -1.372706
```

5.7.1 Residual change

```
# explained rates on log-scale
explainedrate <- lmBV+
sexlogchange+
  mmatlmrate+
tempeffrate+
magelmrate+
inblogchangerate+
gglogchangerate

explainedchange <- funtrans(explainedrate*44)

lmm0 <- lmer(BirthDatePosLog100 ~ 1 + OffspBirthYear +
               (1|MumId)+ (1|MumYOB) + (1|MotherMother),
                   data = birthdata, na.action = "na.omit")

unexplainedchange <- funtrans(fixef(lmm0)[2]*44)-funtrans((explainedrate)*44)
```

5.8 Model with untransformed data

We fit an animal model on untransformed parturition dates to check results are robust to transformation of data and back-transformation of estimates.

```
m0_bdateUntrans <- MCMCglmm(BirthDatePos ~ 1 + OffspSex + MotherStatus +
                       MumAge + MumAge2 + GGImm + Inbreeding,
                     random=~ animal + MumPE + MotherMother + OffspBirthYear + MumYOB,
                     rcov=~units,
                     data=birthdata,
                     ginverse = list(animal=invAmatrix),
                     nitt = 130000, thin = 100, burnin = 30000,
                     pr=TRUE, verbose = FALSE)
save(m0_bdateUntrans, file = "ClusterComputations/AM1Untrans/m0_bdateUntrans")
```

```r
load("ClusterComputations/AM1Untrans/m0_bdateUntrans")
summary(m0_bdateUntrans)
```

```r
unth2 <- m0_bdateUntrans$VCV[,1]/(m0_bdateUntrans$VCV[,1]+
                                  m0_bdateUntrans$VCV[,2]+
                                  m0_bdateUntrans$VCV[,3]+
                                  m0_bdateUntrans$VCV[,5]+
                                  m0_bdateUntrans$VCV[,6])
plot(unth2)
```

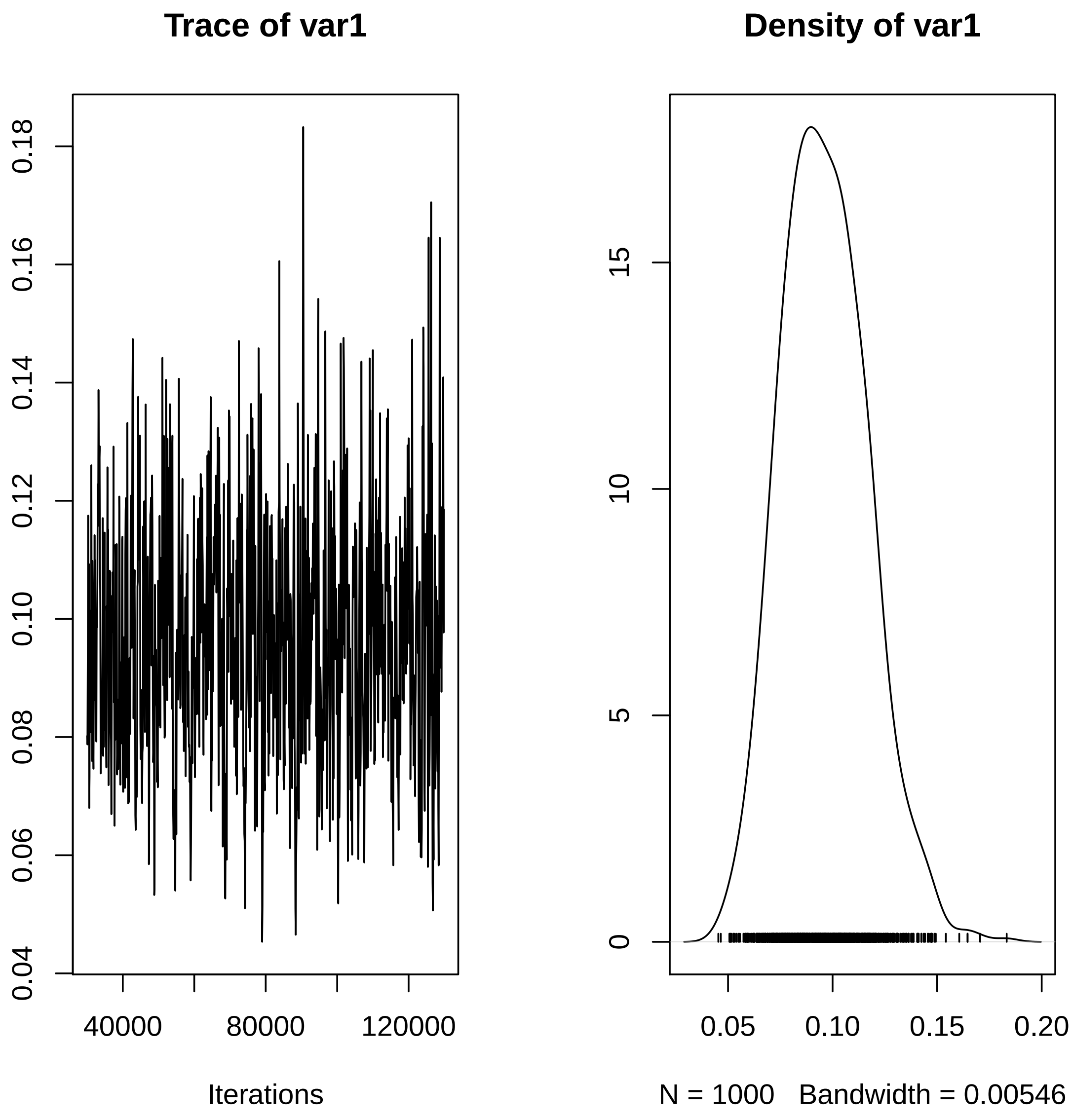


```r
posterior.mode(unth2)

##      var1
## 0.1034284

HPDinterval(unth2)

##           lower     upper
## var1 0.05828282 0.1364901
```

```
## attr(,"Probability")
## [1] 0.95
```

```
BVunt<-m0_bdateUntrans$Sol[,grep(pattern = "animal*",x =colnames(m0_bdateUntrans$Sol))]
namesBVunt<- substr(dimnames(BVunt)[[2]],start = 8,
                 stop = nchar(dimnames(BVunt)[[2]]))

BVunt<-BVunt[,which(namesBVunt %in% birthdata$animal[birthdata$MumYOB>1980])]

animalIDunt<-substr(x = colnames(BVunt),start = 8,
                 stop=nchar(colnames(BVunt)))
pmBVunt<-data.frame(animalIDunt,posterior.mode(BVunt))
names(pmBVunt)<-c("animal","pBV")

meanYunt<-data.frame(tapply(birthdata$OffspBirthYear,birthdata$animal,mean),
          names(tapply(birthdata$OffspBirthYear,birthdata$animal,mean)))
names(meanYunt)<-c("year","animal")

mpmBVunt<-merge(x = pmBVunt,y = meanYunt,by = "animal",all.x=TRUE ,
                all.y = FALSE, sort=FALSE)

lmBVunt<-as.mcmc(apply(BVunt,MARGIN = 1,
                    function(x){coef(lm(x~1+mpmBVunt$year))[2]}))
plot(lmBVunt)
```

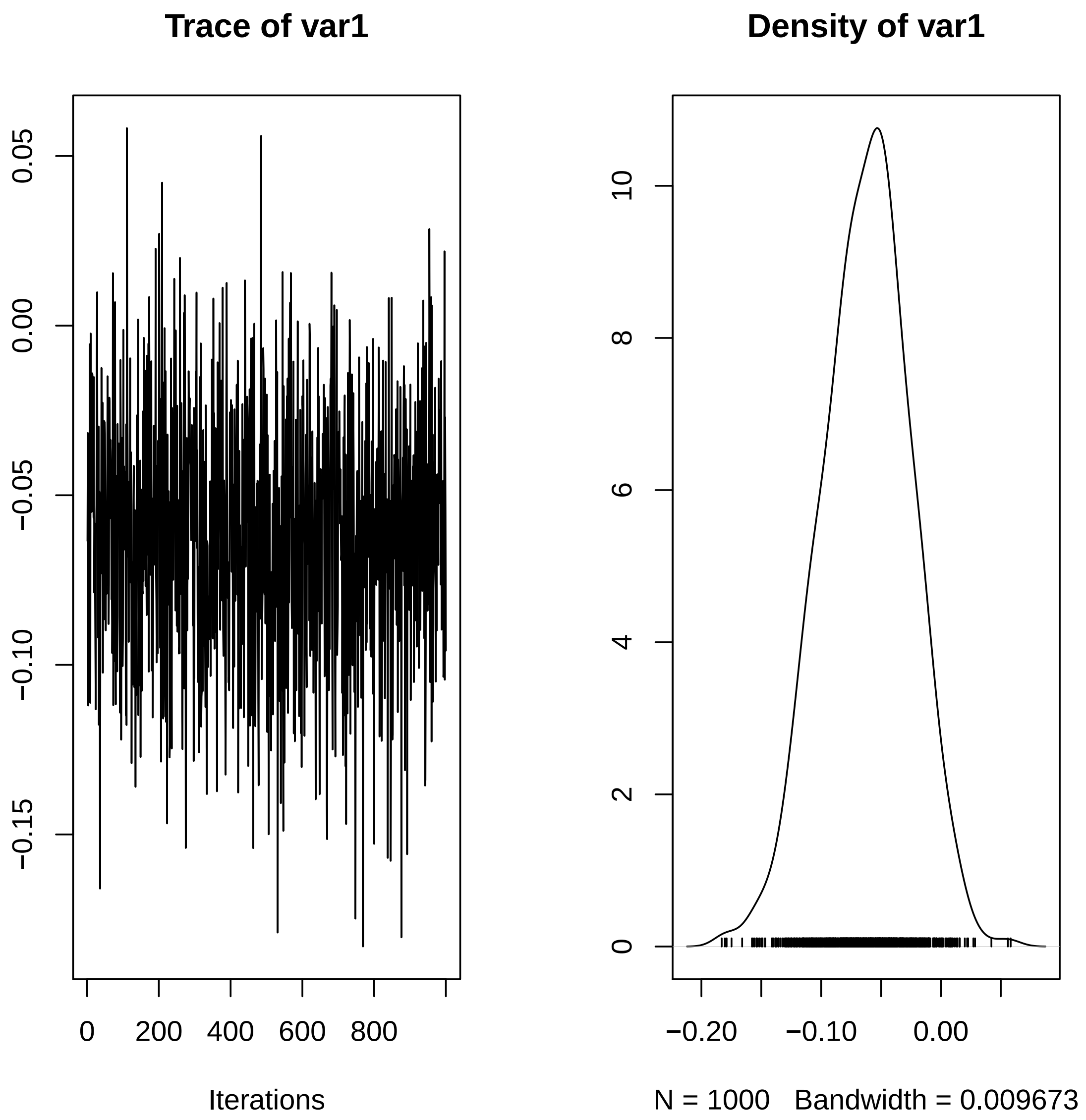


```
HPDinterval(lmBVunt*(max(birthdata$OffspBirthYear)-min(birthdata$OffspBirthYear)))

##          lower     upper
## var1 -5.713917 0.3724408
## attr(,"Probability")
## [1] 0.95

posterior.mode(lmBVunt*(max(birthdata$OffspBirthYear)-min(birthdata$OffspBirthYear)))

##      var1
## -2.247375

mean(lmBVunt>0)

## [1] 0.038
```

6 Selection analysis

6.1 Fitness distribution

We measure fitness as Lifetime Breeding Success (LBS). We will restrict the analysis to females with a fully known life-history (group 1 and 2), irrespective of whether they died of natural cause or were shot. LBS data are not normally distributed, but rather seem to follow a zero-inflated over-dispersed Poisson distribution (Fig. 6).

```
setPar()
hist(birth_fit_data_AllLBS$LBS[!duplicated(birth_fit_data_AllLBS$animal)],
     xlab="Lifetime breeding success", main="")
```

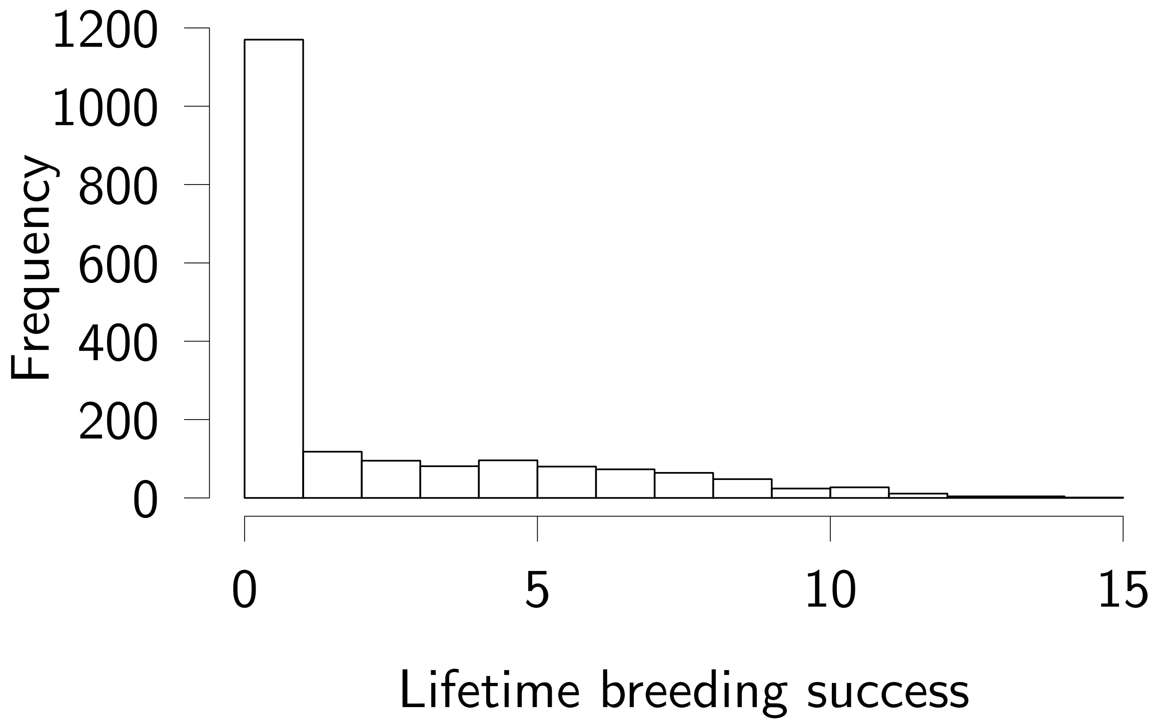

Figure 6: Distribution of lifetime breeding success among females included in the analyses

6.2 Bivariate phenotypic model with LBS

We load data in a long format to fit bivariate animal models with an explicit covariation between the random variance of a trait and the residual variance of another trait. This deals with difference in duplication between parturition dates (multiple observations per female) and lifetime fitness traits (a single observation per female).

```
bdmeltedshrinkedLBS <- read.table("DataArchive/bdmeltedshrinkedLBS.txt",
                                  header = TRUE)
bdmeltedshrinkedLBS$trait <- bdmeltedshrinkedLBS$variable
```

Poisson bivariate model with the covariation between random effect and residuals.

```
prior0<-list( G=list(
  G1=list(V=diag(2), nu=2),
  G2=list(V=diag(2), nu=2),
  G3=list(V=diag(1), nu=0.002)),
  R=list(R1=list(V=diag(2), nu=2, covu=TRUE),
```

```
        R2=list(V=diag(1), nu=0.002)))

mPcovu_Biv_BD_LBS_P<-MCMCglmm(value~variable-1 +
                  at.level(type,"r"):(Sex.y + MotherStatus + MumAge + MumAge2),
                  random=~us(variable):MumYOB + us(variable):MotherMother +
                  us(at.level(type,"r")):BirthYear.y + us(at.level(type,"r")):ID,
                  rcov=~us(at.level(type, "s")):ID+
                  us(at.level(type, "r")):ID:BirthYear.y,
            data=bdmeltedshrinkedLBS,
            prior = prior0,
            nitt =  65000, burnin = 15000, thin = 50, family = NULL)


#save(mPcovu_Biv_BD_LBS_P, file="FilesToLoad/mPcovu_Biv_BD_LBS_Pallg")


priorExp<-list( G=list(
  G1=list(V=diag(2), nu=2, alpha.mu=c(0,0), alpha.V=diag(2)*1000),
  G2=list(V=diag(2), nu=2, alpha.mu=c(0,0), alpha.V=diag(2)*1000),
  G3=list(V=diag(1), nu=0.002)),
  R=list(R1=list(V=diag(2), nu=2, covu=TRUE),
        R2=list(V=diag(1), nu=0.002)))

mPcovu_Biv_BD_LBS_Pexp<-MCMCglmm(value~variable-1 +
                  at.level(type,"r"):(Sex.y + MotherStatus + MumAge + MumAge2),
                  random=~us(variable):MumYOB + us(variable):MotherMother +
                  us(at.level(type,"r")):BirthYear.y + us(at.level(type,"r")):ID,
                  rcov=~us(at.level(type, "s")):ID+
                  us(at.level(type, "r")):ID:BirthYear.y,
            data=bdmeltedshrinkedLBS,
            prior = priorExp,
            nitt =  65000, burnin = 15000, thin = 50, family = NULL)
save(mPcovu_Biv_BD_LBS_Pexp, file="FilesToLoad/mPcovu_Biv_BD_LBS_Pexp")
```

Prior sensitivity for selection:

```
setPar()
plot(density(
  mPcovu_Biv_BD_LBS_Pexp$
    VCV[,'at.level(type, "s").ID:at.level(type, "r").ID'],
  bw=0.5),
  main="Posterior distribution for selection",
  xlab="Selection differential (log-scale)",
  xlim=c(-17,17), ylab="Posterior density")

lines(density(
  mPcovu_Biv_BD_LBS_P$
    VCV[,'at.level(type, "s").ID:at.level(type, "r").ID'],
  bw = 0.5),
  col="red", lty=2)

legend(x = "topright", col=c("black", "red"),
       lwd=3,
       legend = c("With parameter-expanded prior", "With inverse-Gamma prior"),
       lty=c(1,2))
```

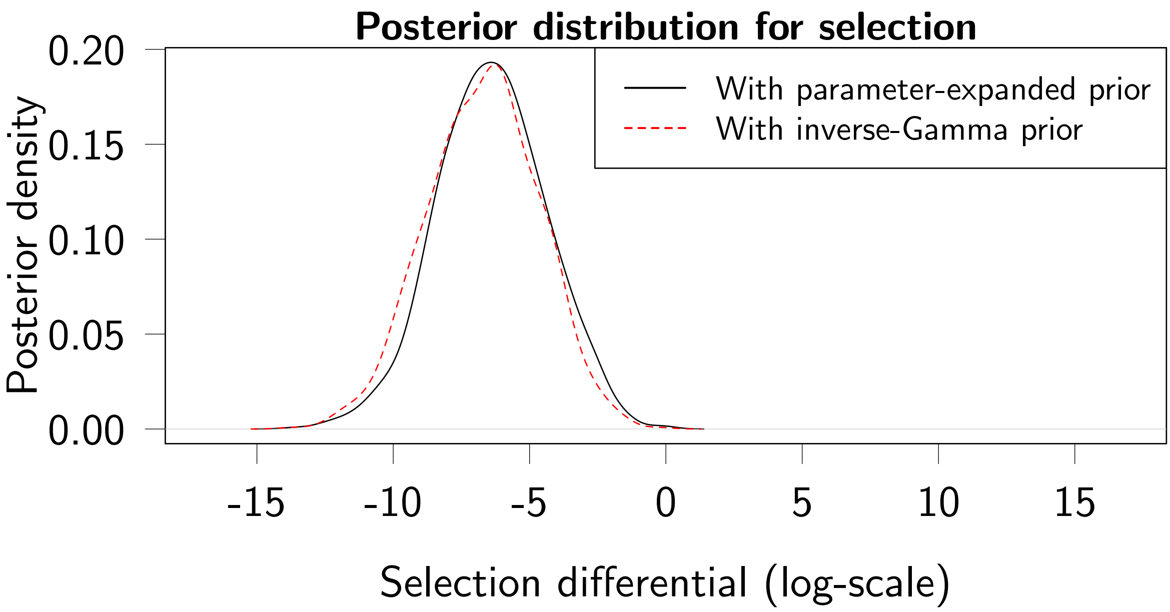


```
smPcovu_Biv_BD_LBS_Pexp <- summary(mPcovu_Biv_BD_LBS_Pexp)
```

```
allranef <- list(mPcovu_Biv_BD_LBS_Pexp$VCV[,"variableBirthDatePosLog100:variableBirthDatePosLog100.I
      mPcovu_Biv_BD_LBS_Pexp$VCV[,"variableLBS:variableBirthDatePosLog100.MumYOB"],
      mPcovu_Biv_BD_LBS_Pexp$VCV[,"variableLBS:variableLBS.MumYOB"],
      mPcovu_Biv_BD_LBS_Pexp$VCV[,"variableBirthDatePosLog100:variableBirthDatePosLog100.MotherMothe
      mPcovu_Biv_BD_LBS_Pexp$VCV[,"variableLBS:variableBirthDatePosLog100.MotherMother"],
      mPcovu_Biv_BD_LBS_Pexp$VCV[,"variableLBS:variableLBS.MotherMother"],
      mPcovu_Biv_BD_LBS_Pexp$VCV[,'at.level(type, "r"):at.level(type, "r").BirthYear.y'],
      mPcovu_Biv_BD_LBS_Pexp$VCV[,'at.level(type, "r").ID:at.level(type, "r").ID'],
      mPcovu_Biv_BD_LBS_Pexp$VCV[,'at.level(type, "s").ID:at.level(type, "r").ID'],
      mPcovu_Biv_BD_LBS_Pexp$VCV[,'at.level(type, "s").ID:at.level(type, "s").ID'],
      mPcovu_Biv_BD_LBS_Pexp$VCV[,'at.level(type, "r"):at.level(type, "r").ID:BirthYear.y'])


tabranef <- data.frame(param = c("Variance Parturition", "Covariance", "Variance LBS",
                          "Variance Parturition", "Covariance", "Variance LBS",
                          "Variance Parturition",
                          "Variance Parturition", "Covariance", "Variance LBS",
                          "Variance Parturition"),
                          estimate=unlist(lapply(allranef, mean)),
              CI=unlist(lapply(allranef, function(x){
                paste0("[",round(HPDinterval(x)[1], 2), " ; ",
                       round(HPDinterval(x)[2], 2), "]")})))
```

Fixed effects

```
allfixef <- list(mPcovu_Biv_BD_LBS_Pexp$Sol[,"variableLBS"],
       mPcovu_Biv_BD_LBS_Pexp$Sol[,"variableBirthDatePosLog100"],
       mPcovu_Biv_BD_LBS_Pexp$Sol[,'at.level(type, "r"):Sex.y'],
       mPcovu_Biv_BD_LBS_Pexp$Sol[,'at.level(type, "r"):MotherStatusNaive'],
       mPcovu_Biv_BD_LBS_Pexp$Sol[,'at.level(type, "r"):MotherStatusSummeryeld'],
       mPcovu_Biv_BD_LBS_Pexp$Sol[,'at.level(type, "r"):MotherStatusTrueyeld'],
       mPcovu_Biv_BD_LBS_Pexp$Sol[,'at.level(type, "r"):MotherStatusWinteryeld'],
       mPcovu_Biv_BD_LBS_Pexp$Sol[,'at.level(type, "r"):MumAge'],
       mPcovu_Biv_BD_LBS_Pexp$Sol[,'at.level(type, "r"):MumAge2']
     )
```

Table 2: Variance components

param	estimate	CI
Variance Parturition	12.395	[0 ; 42.39]
Covariance	1.133	[-15.47 ; 17.2]
Variance LBS	9.080	[5.26 ; 12.93]
Variance Parturition	29.985	[0 ; 69.99]
Covariance	-0.031	[-0.55 ; 0.34]
Variance LBS	0.006	[0 ; 0.02]
Variance Parturition	157.481	[89.12 ; 250.5]
Variance Parturition	219.364	[150.26 ; 290.62]
Covariance	-6.397	[-10.09 ; -2.44]
Variance LBS	0.987	[0.84 ; 1.13]
Variance Parturition	957.034	[909.11 ; 1006.62]

```
tabfixef <- data.frame(param = c("Intercept",
                          "Intercept",
                          "Offspring sex",
                          "Female's Reproductive Status: hfill Naive",
                          "hfill Summer Yeld",
                          "hfill True Yeld",
                          "hfill Winter Yeld",
                          "Female's age", "Female's age squared"),
                          estimate=unlist(lapply(allfixef, mean)),
              CI=unlist(lapply(allfixef, function(x){
                paste0("[",round(HPDinterval(x)[1], 2), " ; ",
                       round(HPDinterval(x)[2], 2), "]")})))
```

Table 3: Fixed effects

param	estimate	CI
Intercept	-1.560	[-1.71 ; -1.4]
Intercept	397.555	[384.91 ; 409.66]
Offspring sex	1.824	[0.17 ; 3.75]
Female's Reproductive Status: hfill Naive	-16.811	[-21.11 ; -11.33]
hfill Summer Yeld	-27.313	[-30.91 ; -23.37]
hfill True Yeld	-23.434	[-26.79 ; -20.5]
hfill Winter Yeld	-0.803	[-5.18 ; 3.86]
Female's age	-7.389	[-9.69 ; -5.43]
Female's age squared	0.450	[0.35 ; 0.56]

6.3 Considering parturition date as a male trait

Poisson bivariate model with the covariation between random effect and residuals.
With duplicated parturition date per year

```
meltfitbirthMales <- read.csv("CleanData/meltfitbirthMales.csv")
summary(meltfitbirthMales)

prior0<-list( G=list(
  G1=list(V=diag(2), nu=2),
  G2=list(V=diag(1), nu=0.002),
  G3=list(V=diag(1), nu=0.002),
  G4=list(V=diag(1), nu=0.002),
  G5=list(V=diag(1), nu=0.002)),
  R=list(R1=list(V=diag(2), nu=3, covu=TRUE),
         R2=list(V=diag(1), nu=0.002)))

mult <- 20
```

```
mPcovu_Biv_Males_Plong<-MCMCglmm(value~variable-1 +
                  at.level(type,"d"):(MotherStatus + MumAge + MumAge2),
                  random=~  idh(traits):BirthYear.x+
                    us(traits):MumCode + #focal mother repeat
                  us(at.level(type,"d")):BirthYear.y + +#year effect
                    us(at.level(type,"d")):(animal:BirthYear.y) +#male by year effect
                    us(at.level(type,"d")):animal,#male effect for birth date
                  rcov=~us(at.level(type, "s")):animal+# male fitness residual
                  us(at.level(type, "d")):(animal:BirthYear.y:MumCode), #
            data=meltfitbirthMales,
            prior = prior0,
            nitt =  13000*mult, burnin = 3000*mult, thin = 10*mult, family = NULL)

summary(mPcovu_Biv_Males_Plong)
```

Selection:

```
load("FilesToLoad/mPcovu_Biv_Males_Plong")

#Selection differential
Spm <- mPcovu_Biv_Males_Plong$VCV[,'at.level(type, "s").animal:at.level(type, "d").animal']
Spmdays <- exp(((Spm)+
                    initialmean)/100 + vardat/2)-
    exp((initialmean)/100+ vardat/2)

HPDinterval(Spmdays)

##           lower     upper
## var1 -0.9257289 0.4032322
## attr(,"Probability")
## [1] 0.95

posterior.mode(Spmdays)

##       var1
## -0.3662021
```

Repeatability:

```
BDmalesRepWY <-
  mPcovu_Biv_Males_Plong$VCV[,'at.level(type, "s").animal:at.level(type, "s").animal'] /
  (mPcovu_Biv_Males_Plong$VCV[,'at.level(type, "s").animal:at.level(type, "s").animal'] +
  mPcovu_Biv_Males_Plong$VCV[,'at.level(type, "d"):at.level(type, "d").animal:BirthYear.y'] +
  mPcovu_Biv_Males_Plong$VCV[,'at.level(type, "d"):at.level(type, "d").MumCode']+
  mPcovu_Biv_Males_Plong$VCV[,'at.level(type, "d"):at.level(type, "d").BirthYear.y'])

BDmalesRepBY <-
  mPcovu_Biv_Males_Plong$VCV[,'at.level(type, "s").animal:at.level(type, "s").animal'] /
  (mPcovu_Biv_Males_Plong$VCV[,'at.level(type, "s").animal:at.level(type, "s").animal'] +
         mPcovu_Biv_Males_Plong$VCV[,'at.level(type, "d"):at.level(type, "d").MumCode']+
     mPcovu_Biv_Males_Plong$VCV[,'at.level(type, "d"):at.level(type, "d").BirthYear.y'])

posterior.mode(BDmalesRepWY)

##       var1
## 0.01382339

HPDinterval(BDmalesRepWY)
```

```
##            lower      upper
## var1 0.00885064 0.02036934
## attr(,"Probability")
## [1] 0.95
```

Predicted response to selection assuming the heritability is at its maximum possible value (i.e., heritability = repeatability).

```
#Predicted response to selection
Rpmdays <- exp(((BDmalesRepWY*Spm/16) * (max(birthdata$MumYOB)-min(birthdata$MumYOB))+
                    initialmean)/100 + vardat/2)-
    exp((initialmean)/100+ vardat/2)

posterior.mode(Rpmdays)

##        var1
## -0.01195498

HPDinterval(Rpmdays)

##             lower     upper
## var1 -0.04206434 0.0153526
## attr(,"Probability")
## [1] 0.95
```

Extract estimates to make a table:

```
allranef <- list(mPcovu_Biv_Males_Plong$VCV[,"traitsBirthDatePosLog100.BirthYear.x"],
      mPcovu_Biv_Males_Plong$VCV[,"traitsMaleLBS.BirthYear.x"],
      mPcovu_Biv_Males_Plong$VCV[,'at.level(type, "d"):at.level(type, "d").MumCode'],
      mPcovu_Biv_Males_Plong$VCV[,'at.level(type, "d"):at.level(type, "d").animal:BirthYear.y'],
      mPcovu_Biv_Males_Plong$VCV[,'at.level(type, "d").animal:at.level(type, "d").animal'],
      mPcovu_Biv_Males_Plong$VCV[,'at.level(type, "s").animal:at.level(type, "d").animal'],
      mPcovu_Biv_Males_Plong$VCV[,'at.level(type, "s").animal:at.level(type, "s").animal'],
      mPcovu_Biv_Males_Plong$VCV[,'at.level(type, "d"):at.level(type, "d").animal:BirthYear.y:MumCod


tabranef <- data.frame(param = c("Variance Parturition","Variance LBS",
                          "Variance Parturition",
                          "Variance Parturition",
                          "Variance Parturition", "Covariance", "Variance LBS",
                          "Variance Parturition"),
                          estimate=unlist(lapply(allranef, mean)),
              CI=unlist(lapply(allranef, function(x){
                paste0("[",round(HPDinterval(x)[1], 2), " ; ",
                       round(HPDinterval(x)[2], 2), "]")})))
```

Table 4: Variance components

param	estimate	CI
Variance Parturition	194.761	[14.94 ; 335.75]
Variance LBS	1.890	[0.7 ; 3.38]
Variance Parturition	143.613	[107.06 ; 183.17]
Variance Parturition	685.803	[556.86 ; 823.95]
Variance Parturition	0.388	[0.09 ; 0.93]
Covariance	-0.681	[-1.99 ; 0.85]
Variance LBS	15.037	[11.17 ; 19.59]
Variance Parturition	344.069	[310.7 ; 378.88]

6.4 Bivariate animal model

Make sure we have all male and female fitness without duplication and with the parturition data.

```
meltfitbirth <- read.csv("CleanData/meltfitbirth.csv")

priorPEXP<-list( G=list(
  G1=list(V=diag(2), nu=2, alpha.mu=rep(0,2), alpha.V=diag(2)*1000),
  G2=list(V=diag(2), nu=2, alpha.mu=rep(0,2), alpha.V=diag(2)*1000),
  G3=list(V=diag(2), nu=2, alpha.mu=rep(0,2), alpha.V=diag(2)*1000),
  G4=list(V=diag(1), nu=0.002, alpha.mu=rep(0,1), alpha.V=diag(1)*1000)),
  R=list(R1=list(V=diag(2), nu=2, covu=TRUE),
         R2=list(V=diag(1), nu=0.002)))

mult <- 20

mAcovUBiv <- MCMCglmm(value~variable-1 + variable:(GGImm+Sex+Inbreeding)+
             at.level(traits,c("BirthDatePosLog100")):
               (MotherStatus + MumAge + MumAge2 + OffspBirthYear),
         random =~ us(traits):animal + us(traits):BirthYear + us(traits):MumId +
               us(at.level(traits,c("BirthDatePosLog100"))):OffspBirthYear+
               us(at.level(traits,c("BirthDatePosLog100"))):Code,
         rcov=~us(at.level(traits, "LBS")):Code+
                  us(at.level(traits,c("BirthDatePosLog100") )):Code:OffspBirthYear,
         data = meltfitbirth, prior=priorPEXP,
         ginverse = list(animal=invAmatrix),
         nitt = 13000*mult, burnin = 3000*mult, thin = 10*mult,
         family = NULL)
save(mAcovUBiv, file = "FilesToLoad/mAcovUBiv")
summary(mAcovUBiv)
```

```
load("FilesToLoad/mAcovUBiv")
 daychangescovA <- funtrans(mAcovUBiv$VCV[,2]*((2015-1972)/16))
#plot(daychangescovA)
posterior.mode(daychangescovA)

##      var1
## -4.896172

HPDinterval(daychangescovA)

##          lower     upper
## var1 -10.64984 -0.730384
## attr(,"Probability")
## [1] 0.95
```

Random effects bivariate animal model

```
allranef <- list(mAcovUBiv$VCV[,"traitsBirthDatePosLog100:traitsBirthDatePosLog100.animal"],
      mAcovUBiv$VCV[,"traitsLBS:traitsBirthDatePosLog100.animal"],
      mAcovUBiv$VCV[,"traitsLBS:traitsLBS.animal"],
      mAcovUBiv$VCV[,"traitsBirthDatePosLog100:traitsBirthDatePosLog100.BirthYear"],
      mAcovUBiv$VCV[,"traitsLBS:traitsBirthDatePosLog100.BirthYear"],
      mAcovUBiv$VCV[,"traitsLBS:traitsLBS.BirthYear"],
      mAcovUBiv$VCV[,'traitsBirthDatePosLog100:traitsBirthDatePosLog100.MumId'],
      mAcovUBiv$VCV[,'traitsLBS:traitsBirthDatePosLog100.MumId'],
      mAcovUBiv$VCV[,'traitsLBS:traitsLBS.MumId'],
      mAcovUBiv$VCV[,'at.level(traits, c("BirthDatePosLog100")):at.level(traits, c("BirthDatePosLog1
```

```
     mAcovUBiv$VCV[,'at.level(traits, c("BirthDatePosLog100")).Code:at.level(traits, c("BirthDatePo
     mAcovUBiv$VCV[,'at.level(traits, "LBS").Code:at.level(traits, c("BirthDatePosLog100")).Code'],
     mAcovUBiv$VCV[,'at.level(traits, "LBS").Code:at.level(traits, "LBS").Code'],
     mAcovUBiv$VCV[,'at.level(traits, c("BirthDatePosLog100")):at.level(traits, c("BirthDatePosLog1


tabranef <- data.frame(param = c("Variance Parturition", "Covariance", "Variance LBS",
                          "Variance Parturition", "Covariance", "Variance LBS",
                          "Variance Parturition", "Covariance", "Variance LBS",
                          "Variance Parturition",
                          "Variance Parturition", "Covariance", "Variance LBS",
                          "Variance Parturition"),
                          estimate=unlist(lapply(allranef, mean)),
            CI=unlist(lapply(allranef, function(x){
              paste0("[",round(HPDinterval(x)[1], 2), " ; ",
                     round(HPDinterval(x)[2], 2), "]")})))
```

Table 5: Variance components

param	estimate	CI
Variance Parturition	211.257	[136.97 ; 284.08]
Covariance	-4.937	[-9.56 ; -0.58]
Variance LBS	1.077	[0.75 ; 1.4]
Variance Parturition	6.951	[0 ; 26.62]
Covariance	-1.548	[-12.08 ; 6.65]
Variance LBS	6.312	[3.54 ; 9.15]
Variance Parturition	26.383	[0 ; 71.51]
Covariance	-0.016	[-0.71 ; 0.65]
Variance LBS	0.020	[0 ; 0.08]
Variance Parturition	97.916	[43.81 ; 150.51]
Variance Parturition	11.895	[0.17 ; 50.19]
Covariance	-3.935	[-11.64 ; 1.56]
Variance LBS	3.587	[3.16 ; 4.03]
Variance Parturition	962.298	[915.22 ; 1016.81]

Fixed effects

```
allfixef <- list(mAcovUBiv$Sol[,"variableLBS"],
                 mAcovUBiv$Sol[,"variableLBS:GGImm"],
                 mAcovUBiv$Sol[,"variableLBS:Inbreeding"],
      mAcovUBiv$Sol[,"variableBirthDatePosLog100"],
      mAcovUBiv$Sol[,"variableBirthDatePosLog100:GGImm"],
      mAcovUBiv$Sol[,"variableBirthDatePosLog100:Inbreeding"],
      mAcovUBiv$Sol[,'at.level(traits, c("BirthDatePosLog100")):MotherStatusNaive'],
      mAcovUBiv$Sol[,'at.level(traits, c("BirthDatePosLog100")):MotherStatusSummeryeld'],
      mAcovUBiv$Sol[,'at.level(traits, c("BirthDatePosLog100")):MotherStatusTrueyeld'],
      mAcovUBiv$Sol[,'at.level(traits, c("BirthDatePosLog100")):MotherStatusMilk'],
      mAcovUBiv$Sol[,'at.level(traits, c("BirthDatePosLog100")):MumAge'],
      mAcovUBiv$Sol[,'at.level(traits, c("BirthDatePosLog100")):MumAge2'],
      mAcovUBiv$Sol[,'at.level(traits, c("BirthDatePosLog100")):OffspBirthYear']
    )


tabfixef <- data.frame(param = c("Intercept",
                                 "Genetic group",
                                 "Inbreeding",
                          "Intercept",
                                 "Genetic group",
                                 "Inbreeding",
                          "Female's Reproductive Status: hfill Naive",
```

```
                        "hfill Summer Yeld",
                        "hfill True Yeld",
                        "hfill Milk",
                        "Female's age", "Female's age squared",
                        "Offspring birth year"),
                        estimate=unlist(lapply(allfixef, mean)),
          CI=unlist(lapply(allfixef, function(x){
            paste0("[",round(HPDinterval(x)[1], 2), " ; ",
                   round(HPDinterval(x)[2], 2), "]")})))
```

Table 6: Fixed effects

param	estimate	CI
Intercept	-2.204	[-2.92 ; -1.46]
Genetic group	2.015	[1.56 ; 2.47]
Inbreeding	-17.693	[-23.33 ; -11.88]
Intercept	1149.012	[434.91 ; 1877.48]
Genetic group	-2.046	[-11.09 ; 8.87]
Inbreeding	59.318	[-34.12 ; 154.82]
Female's Reproductive Status: hfill Naive	-15.016	[-20.37 ; -9.43]
hfill Summer Yeld	-26.373	[-31.75 ; -21.46]
hfill True Yeld	-21.861	[-26.42 ; -16.97]
hfill Milk	0.563	[-4.03 ; 5.19]
Female's age	-7.223	[-9.23 ; -4.91]
Female's age squared	0.431	[0.32 ; 0.53]
Offspring birth year	-0.372	[-0.74 ; -0.01]

6.5 Multivariate breeder's equation

Birth weight and birth dates are phenotypically correlated, both under selection, and likely genetically correlated. Selection on birth weight may therefore affect the response to selection in parturition date. We investigate this using a bivariate breeder's equation, for which we estimate an additive genetic G-matrix of parturition date and calf weight, and a multivariate selection gradient.

6.5.1 Animal model of calf weight and parturition date

```
prior0_Biv_BD_BW <- list(
  G=list(
    G1=list(V=diag(2), nu=2, alpha.mu=rep(0,2), alpha.V=diag(2)*1000),
    G2=list(V=diag(2), nu=2, alpha.mu=rep(0,2), alpha.V=diag(2)*1000),
    G3=list(V=diag(2), nu=2, alpha.mu=rep(0,2), alpha.V=diag(2)*1000),
    G3=list(V=diag(2), nu=2, alpha.mu=rep(0,2), alpha.V=diag(2)*1000)),
  R=list(
    V=diag(2), nu=2))

multip <- 50

m0_Biv_BD_BW <-
  MCMCglmm(cbind(BirthDatePosLog100, BirthWgt) ~ trait-1+
             trait:(GGImm + Sex.y + MotherStatus + MumAge + MumAge2) ,
           random=~ us(trait):animal +  us(trait):MotherMother +
             us(trait):BirthYear.x +us(trait):BirthYear.y,
           rcov=~us(trait):units,
           prior=prior0_Biv_BD_BW,
           data=birth_fit_data_AllLBS,
           ginverse = list(animal=invAmatrix),
           nitt = 13000*multip, thin = 10*multip, burnin = 3000*multip,
           pr=FALSE, verbose = TRUE, family = c("gaussian", "gaussian"))
```

```
summary(m0_Biv_BD_BW)

plot(m0_Biv_BD_BW$VCV[,2])
HPDinterval(m0_Biv_BD_BW$VCV[,2])
HPDinterval(m0_Biv_BD_BW$VCV[,2]/sqrt(m0_Biv_BD_BW$VCV[,1]*m0_Biv_BD_BW$VCV[,4]))
plot(m0_Biv_BD_BW$VCV[,2]/sqrt(m0_Biv_BD_BW$VCV[,1]*m0_Biv_BD_BW$VCV[,4]))
posterior.mode(m0_Biv_BD_BW$VCV[,2]/sqrt(m0_Biv_BD_BW$VCV[,1]*m0_Biv_BD_BW$VCV[,4]))
```

6.5.2 Phenotypic multivariate selection

Long format data for the two phenotypes of interest and lifetime fitness.

```
bdbwmeltedshrinkedLBS <- read.table(file = "DataArchive/bdbwmeltedshrinkedLBS.txt",
                                    header = TRUE)
```

Trivariate model for bivariate selection

```
priorPEXP<-list( G=list(
  G1=list(V=diag(3), nu=3, alpha.mu=rep(0,3), alpha.V=diag(3)*1000),
  G2=list(V=diag(3), nu=3, alpha.mu=rep(0,3), alpha.V=diag(3)*1000),
  G3=list(V=diag(2), nu=2, alpha.mu=rep(0,2), alpha.V=diag(2)*1000)),
  R=list(R1=list(V=diag(3), nu=3, covu=TRUE),
         R2=list(V=diag(2), nu=2)))


multipl <- 2
m0_covu_Tri_BD_BW_LBS_P<-MCMCglmm(value~variable-1 + variable:GGImm +
      at.level(traits,c("BirthWgt", "BirthDatePosLog100")):(
        Sex.y + MotherStatus + MumAge + MumAge2),
    random=~us(variable):MumYOB +
      us(variable):MotherMother +
      us(at.level(traits,c("BirthWgt", "BirthDatePosLog100"))):BirthYear.y +
      us(at.level(traits,c("BirthWgt", "BirthDatePosLog100"))):ID,
    rcov=~us(at.level(traits, "LBS")):ID+
      us(at.level(traits,c("BirthWgt", "BirthDatePosLog100") )):ID:BirthYear.y,
    data=bdbwmeltedshrinkedLBS[bdbwmeltedshrinkedLBS$Group!=3, ],
    prior = priorPEXP, family=NULL,
    nitt =  13000*multipl, burnin = 3000*multipl, thin = 10*multipl)
summary(m0_covu_Tri_BD_BW_LBS_P)
plot(m0_covu_Tri_BD_BW_LBS_P$VCV)

save(m0_covu_Tri_BD_BW_LBS_P, file="FilesToLoad/m0_covu_Tri_BD_BW_LBS_P")
```

Variance components are indexed as follow: 1-9 for MumYob
10-18 for MotherMother
19-22 for birthyear
23-31 for ID GR
32-35 for resid

Applying multi-variate breeder's equation:

```
load(file = "FilesToLoad/m0_covu_Tri_BD_BW_LBS_P")
load(file = "FilesToLoad/m0_Biv_BD_BW")

multivarBreederLBS <- list()
multivarBreederLBSnoCG <- list()
Gmat <- list()
```

```
Pmat <- list()
Slist <- list()
Gradlist <- list()
for (i in 1:length(m0_covu_Tri_BD_BW_LBS_P$VCV[,1]))
{
  G_bw_bd <- matrix(c(m0_Biv_BD_BW$VCV[i,4], #BW_BW
                      m0_Biv_BD_BW$VCV[i,2], #BW_BD
                      m0_Biv_BD_BW$VCV[i,3], # BD_BW
                      m0_Biv_BD_BW$VCV[i,1]), nrow = 2) # BD_BD
  S_bw_bd <- c(m0_covu_Tri_BD_BW_LBS_P$VCV[i,29:30] +
               m0_covu_Tri_BD_BW_LBS_P$VCV[i,17:16])
  P <- matrix(c(m0_covu_Tri_BD_BW_LBS_P$VCV[i,23]+
                m0_covu_Tri_BD_BW_LBS_P$VCV[i,32]+
                m0_covu_Tri_BD_BW_LBS_P$VCV[i,14], #BW_BW
       m0_covu_Tri_BD_BW_LBS_P$VCV[i,24]+
         m0_covu_Tri_BD_BW_LBS_P$VCV[i,33]+
         m0_covu_Tri_BD_BW_LBS_P$VCV[i,13],
       m0_covu_Tri_BD_BW_LBS_P$VCV[i,26]+
         m0_covu_Tri_BD_BW_LBS_P$VCV[i,34]+m0_covu_Tri_BD_BW_LBS_P$VCV[i,11],
       m0_covu_Tri_BD_BW_LBS_P$VCV[i,27]+
         m0_covu_Tri_BD_BW_LBS_P$VCV[i,35]+
         m0_covu_Tri_BD_BW_LBS_P$VCV[i,10]), nrow = 2)

   Grad_bw_bd <- S_bw_bd %*% solve(P)

   Gmat[[i]] <- G_bw_bd
   Pmat[[i]] <- P
   Slist[[i]] <- S_bw_bd
   Gradlist[[i]] <- Grad_bw_bd
  multivarBreederLBS[[i]] <- Grad_bw_bd %*% G_bw_bd
  multivarBreederLBSnoCG[[i]] <- Grad_bw_bd %*% (G_bw_bd * diag(2))
}
```

6.5.3 Calculation of selection differentials and gradients

Poisson estimates are already on a relative fitness scale. Selection must always be divided by two, because selection on the parturition date acts directly only on females, and divided by generation time to convert prediction of response to selection into years.

```
GenerationTime = 8

####
# selection differential
posterior.mode(funtrans((mPcovu_Biv_BD_LBS_P$VCV[,6] +
                           mPcovu_Biv_BD_LBS_P$VCV[,11])* 0.5))

##       var1
## -1.376971

# predicted response to selection
posterior.mode(funtrans((44/GenerationTime)*h2BDPL *
                          (mPcovu_Biv_BD_LBS_P$VCV[,6] +
                             mPcovu_Biv_BD_LBS_P$VCV[,11])* 0.5))

##      var1
## -1.30144

# untransformed
```

```r
S_p <- (mPcovu_Biv_BD_LBS_P$VCV[,6] + mPcovu_Biv_BD_LBS_P$VCV[,11])* 0.5/GenerationTime

R_p <- h2BDPL * S_p #breeder's equation
```

Multivariate breeder's equation:
P-matrix

```r
Pmode <- matrix(c(posterior.mode(as.mcmc(unlist(lapply(Pmat, function(x){x[1,1]})))),
         posterior.mode(as.mcmc(unlist(lapply(Pmat, function(x){x[1,2]})))),
         posterior.mode(as.mcmc(unlist(lapply(Pmat, function(x){x[2,1]})))),
         posterior.mode(as.mcmc(unlist(lapply(Pmat, function(x){x[2,2]}))))), nrow=2)

#phenotypic correlation
posterior.mode(as.mcmc(unlist(lapply(Pmat, function(x){x[1,2]/sqrt(x[1,1]*x[2,2])})) ) )

##      var1
## 0.1164996

HPDinterval(as.mcmc(unlist(lapply(Pmat, function(x){x[1,2]/sqrt(x[1,1]*x[2,2])})) ) )

##           lower     upper
## var1 0.04943641 0.1674889
## attr(,"Probability")
## [1] 0.95
```

G-matrix

```r
Gmode <- matrix(c(posterior.mode(as.mcmc(unlist(lapply(Gmat, function(x){x[1,1]})))),
         posterior.mode(as.mcmc(unlist(lapply(Gmat, function(x){x[1,2]})))),
         posterior.mode(as.mcmc(unlist(lapply(Gmat, function(x){x[2,1]})))),
         posterior.mode(as.mcmc(unlist(lapply(Gmat, function(x){x[2,2]}))))), nrow=2)
```

genetic covariance

```r
posterior.mode(as.mcmc(unlist(lapply(Gmat, function(x){x[1,2]})) ) )

##      var1
## -1.781026

HPDinterval(as.mcmc(unlist(lapply(Gmat, function(x){x[1,2]})) ) )

##          lower     upper
## var1 -4.381197 0.5569194
## attr(,"Probability")
## [1] 0.95
```

genetic correlation

```r
posterior.mode(as.mcmc(unlist(lapply(Gmat, function(x){x[1,2]/sqrt(x[1,1]*x[2,2])})) ) )

##      var1
## -0.155703

HPDinterval(as.mcmc(unlist(lapply(Gmat, function(x){x[1,2]/sqrt(x[1,1]*x[2,2])})) ) )

##           lower      upper
## var1 -0.3296443 0.05137007
## attr(,"Probability")
## [1] 0.95

mean(as.mcmc(unlist(lapply(Gmat, function(x){x[1,2]/sqrt(x[1,1]*x[2,2])})) ) >0)

## [1] 0.081
```

Additive genetic variance in birth weight

```
posterior.mode(as.mcmc(unlist(lapply(Gmat, function(x){x[1,1]})) ) )

##      var1
## 0.6780077

HPDinterval(as.mcmc(unlist(lapply(Gmat, function(x){x[1,1]})) ) )

##          lower     upper
## var1 0.5701585 0.9011971
## attr(,"Probability")
## [1] 0.95
```

Heritability of birth weight

```
posterior.mode(as.mcmc(unlist(lapply(Gmat, function(x){x[1,1]})) ) /
                 as.mcmc(unlist(lapply(Pmat, function(x){x[1,1]})) ))

##     var1
## 0.470881

HPDinterval(as.mcmc(unlist(lapply(Gmat, function(x){x[1,1]})) ) /
                 as.mcmc(unlist(lapply(Pmat, function(x){x[1,1]})) ))

##          lower     upper
## var1 0.3679855 0.6050444
## attr(,"Probability")
## [1] 0.95
```

Multivariate selection

```
#Selection differentials
S_MV_BW <- as.mcmc(as.numeric(unlist(lapply(Slist, function(x){x[1]} )))) /
  (2*GenerationTime)

S_MV_BD <- as.mcmc(as.numeric(unlist(lapply(Slist, function(x){x[2]} )))) /
  (2 * GenerationTime)

# selection gradients
grad_MV_BW <- as.mcmc(as.numeric(unlist(lapply(Gradlist, function(x){x[1]} )))) /
  (2 * GenerationTime)

grad_MV_BD <- as.mcmc(as.numeric(unlist(lapply(Gradlist, function(x){x[2]} )))) /
  (2 * GenerationTime)

posterior.mode(grad_MV_BD); HPDinterval(grad_MV_BD) ; mean(grad_MV_BD>0)

##          var1
## -0.0003113802
##               lower         upper
## var1 -0.000577959 -4.377817e-05
## attr(,"Probability")
## [1] 0.95
## [1] 0.013

posterior.mode(grad_MV_BW); HPDinterval(grad_MV_BW) ; mean(grad_MV_BW>0)

##          var1
## -0.0008664406
```

```
##             lower       upper
## var1 -0.01041533 0.009374859
## attr(,"Probability")
## [1] 0.95
## [1] 0.492
```

Multivariate breeder's equation prediction

```
#responses per year
RmvBW_lbs <- as.mcmc(unlist(lapply(multivarBreederLBS, FUN = function(x){x[1]}))) /
  (2*GenerationTime)

RmvBD_lbs <- as.mcmc(unlist(lapply(multivarBreederLBS, FUN = function(x){x[2]}))) /
  (2*GenerationTime)

posterior.mode(funtrans(44*RmvBD_lbs), adjust = 1)

##      var1
## -1.416777

HPDinterval(funtrans(44*RmvBD_lbs))

##           lower     upper
## var1 -2.706375 0.1144627
## attr(,"Probability")
## [1] 0.95

mean(funtrans(RmvBD_lbs)>0)

## [1] 0.025

univarMVr <- (as.mcmc(unlist(lapply(S_MV_BD, FUN = function(x){x[1]})))*
  as.mcmc(unlist(lapply(Gmat, function(x){x[2,2]})) ) /
               as.mcmc(unlist(lapply(Pmat, function(x){x[2,2]})) ) )

posterior.mode(funtrans(44*univarMVr)-funtrans(44*RmvBD_lbs) , adjust = 0.5)

##        var1
## 0.005339141

HPDinterval(funtrans(44*univarMVr)-funtrans(44*RmvBD_lbs))

##          lower     upper
## var1 -0.70576 0.5546917
## attr(,"Probability")
## [1] 0.95
```

6.6 Difference between models including or excluding shot females

```
bdmeltedshrinkedLBS$ShotNotShot <- NA
for (i in 1:nrow(bdmeltedshrinkedLBS))
{
  bdmeltedshrinkedLBS$ShotNotShot[i] <- birth_fit_data_AllLBS$ShotOrNot[
            birth_fit_data_AllLBS$animal==bdmeltedshrinkedLBS$ID[i]][1]
}
```

Model excluding shot females:

```
prior0<-list( G=list(
  G1=list(V=diag(2), nu=2),
  G2=list(V=diag(2), nu=2),
  G3=list(V=diag(1), nu=0.002)),
  R=list(R1=list(V=diag(2), nu=2, covu=TRUE),
         R2=list(V=diag(1), nu=0.002)))

mPcovu_Biv_BD_LBS_P_notshot<-MCMCglmm(value~variable-1 +
                  at.level(type,"r"):(Sex.y + MotherStatus + MumAge + MumAge2),
                  random=~us(variable):MumYOB + us(variable):MotherMother +
                  us(at.level(type,"r")):BirthYear.y + us(at.level(type,"r")):ID,
                  rcov=~us(at.level(type, "s")):ID+
                  us(at.level(type, "r")):ID:BirthYear.y,
                  data=bdmeltedshrinkedLBS[bdmeltedshrinkedLBS$ShotNotShot!="Shot",],
                  prior = prior0,
                  nitt =  65000, burnin = 15000, thin = 50, family = NULL)


save(mPcovu_Biv_BD_LBS_P_notshot, file="FilesToLoad/mPcovu_Biv_BD_LBS_P_notshot")
```

Model with only natural deaths

```
prior0<-list( G=list(
  G1=list(V=diag(2), nu=2),
  G2=list(V=diag(2), nu=2),
  G3=list(V=diag(1), nu=0.002)),
  R=list(R1=list(V=diag(2), nu=2, covu=TRUE),
         R2=list(V=diag(1), nu=0.002)))

mPcovu_Biv_BD_LBS_P_natdeath<-MCMCglmm(value~variable-1 +
            at.level(type,"r"):(Sex.y + MotherStatus + MumAge + MumAge2),
            random=~us(variable):MumYOB + us(variable):MotherMother +
            us(at.level(type,"r")):BirthYear.y + us(at.level(type,"r")):ID,
            rcov=~us(at.level(type, "s")):ID+
            us(at.level(type, "r")):ID:BirthYear.y,
            data=bdmeltedshrinkedLBS[bdmeltedshrinkedLBS$ShotNotShot=="DeadNotShot",],
            prior = prior0,
            nitt =  65000, burnin = 15000, thin = 50, family = NULL)


save(mPcovu_Biv_BD_LBS_P_natdeath, file="FilesToLoad/mPcovu_Biv_BD_LBS_P_natdeath")
```

Model with only culling deaths

```
prior0<-list( G=list(
  G1=list(V=diag(2), nu=2),
  G2=list(V=diag(2), nu=2),
  G3=list(V=diag(1), nu=0.002)),
  R=list(R1=list(V=diag(2), nu=2, covu=TRUE),
         R2=list(V=diag(1), nu=0.002)))

mPcovu_Biv_BD_LBS_P_culldeath<-MCMCglmm(value~variable-1 +
                  at.level(type,"r"):(Sex.y + MotherStatus + MumAge + MumAge2),
                  random=~us(variable):MumYOB + us(variable):MotherMother +
                  us(at.level(type,"r")):BirthYear.y + us(at.level(type,"r")):ID,
                  rcov=~us(at.level(type, "s")):ID+
                  us(at.level(type, "r")):ID:BirthYear.y,
                  data=bdmeltedshrinkedLBS[bdmeltedshrinkedLBS$ShotNotShot=="Shot",],
```

```
                   prior = prior0,
                   nitt =  65000, burnin = 15000, thin = 50, family = NULL)


save(mPcovu_Biv_BD_LBS_P_culldeath, file="FilesToLoad/mPcovu_Biv_BD_LBS_P_culldeath")
```

Comparison:

```
load("FilesToLoad/mPcovu_Biv_BD_LBS_P_notshot")
load("FilesToLoad/mPcovu_Biv_BD_LBS_P_culldeath")
load("FilesToLoad/mPcovu_Biv_BD_LBS_P_natdeath")

S_p_cull <- (mPcovu_Biv_BD_LBS_P_culldeath$VCV[,6] +
               mPcovu_Biv_BD_LBS_P_culldeath$VCV[,11])*
  0.5/GenerationTime

S_p_nat <- (mPcovu_Biv_BD_LBS_P_natdeath$VCV[,6] +
              mPcovu_Biv_BD_LBS_P_natdeath$VCV[,11])*
  0.5/GenerationTime

S_p <- (mPcovu_Biv_BD_LBS_P$VCV[,6] +
          mPcovu_Biv_BD_LBS_P$VCV[,11])* 0.5/GenerationTime

S_p_noshot <- (mPcovu_Biv_BD_LBS_P_notshot$VCV[,6] +
                 mPcovu_Biv_BD_LBS_P_notshot$VCV[,11])*
  0.5/GenerationTime

HPDinterval(S_p_cull)

##           lower     upper
## var1 -0.1674427 0.2827432
## attr(,"Probability")
## [1] 0.95

posterior.mode(S_p_cull)

##       var1
## 0.01630426

HPDinterval(S_p_noshot - S_p)

##           lower     upper
## var1 -0.5741256 0.2556661
## attr(,"Probability")
## [1] 0.95

mean(S_p_noshot < S_p)

## [1] 0.793

mean(S_p_noshot<S_p_cull)

## [1] 0.999
```

Predicted response from the natural death:

```
Rcull <- S_p_cull*h2BDPL
dayRcull <-exp((Rcull*(max(birthdata$MumYOB)-min(birthdata$MumYOB))+
                  initialmean)/100 + vardat/2)-
    exp((initialmean)/100+ vardat/2)
```

```r
posterior.mode(dayRcull)

##       var1
## 0.09830411

HPDinterval(dayRcull)

##           lower     upper
## var1 -0.6071612 0.8851429
## attr(,"Probability")
## [1] 0.95

Rnat <- S_p_nat*h2BDPL
dayRnat <-exp((Rnat*(max(birthdata$MumYOB)-min(birthdata$MumYOB))+
                 initialmean)/100 + vardat/2)-
    exp((initialmean)/100+ vardat/2)
posterior.mode(dayRnat)

##      var1
## -1.657965

HPDinterval(dayRnat)

##         lower      upper
## var1 -2.97484 -0.5076939
## attr(,"Probability")
## [1] 0.95

Rnoshot <- S_p_noshot*h2BDPL
dayRnoshot <-exp((Rnoshot*(max(birthdata$MumYOB)-min(birthdata$MumYOB))+
                    initialmean)/100 + vardat/2)-
    exp((initialmean)/100+ vardat/2)
HPDinterval(dayRnoshot)

##          lower      upper
## var1 -3.219685 -0.8942868
## attr(,"Probability")
## [1] 0.95

posterior.mode(dayRnoshot)

##      var1
## -1.743714
```

```r
setPar()
colsel <- rgb(c(0,0.2,1),c(0,0.5,0),c(0.7,0.2,0),alpha = 0.8)
sdiffp <- funtrans(S_p*GenerationTime)
sdiffp_noshot <- funtrans(S_p_noshot*GenerationTime)
sdiffp_cull <- funtrans(S_p_cull*GenerationTime)

plot(density(sdiffp, adjust = 1.5), col=colsel[1],
     ylab="Posterior probability density",
     xlab = "Selection differential (days/generation)",
     lwd=5, main="",
     xlim=c(min(c(sdiffp_noshot, sdiffp_cull))*1.1,
            max(c(sdiffp_noshot, sdiffp_cull))*1.1+0.05),
     ylim=c(0,1.8))
lines(density(sdiffp_noshot, adjust = 1.5), col=colsel[2], lwd=5, lty=4)
lines(density(sdiffp_cull, adjust = 1.5), col=colsel[3], lwd=5, lty=3)
```

```
abline(v=0)
abline(v=c(posterior.mode(sdiffp, adjust = 1.5),
           posterior.mode(sdiffp_noshot, adjust = 1.5),
           posterior.mode(sdiffp_cull, adjust = 1.5)),
       col=colsel, lty=2)

legend(x = "topleft", legend = c("Including shot",
                                 "Excluding shot",
                                 "Shot only"),
       col = colsel, lwd=5, bg = "white", lty=c(1,2,4,3))
```

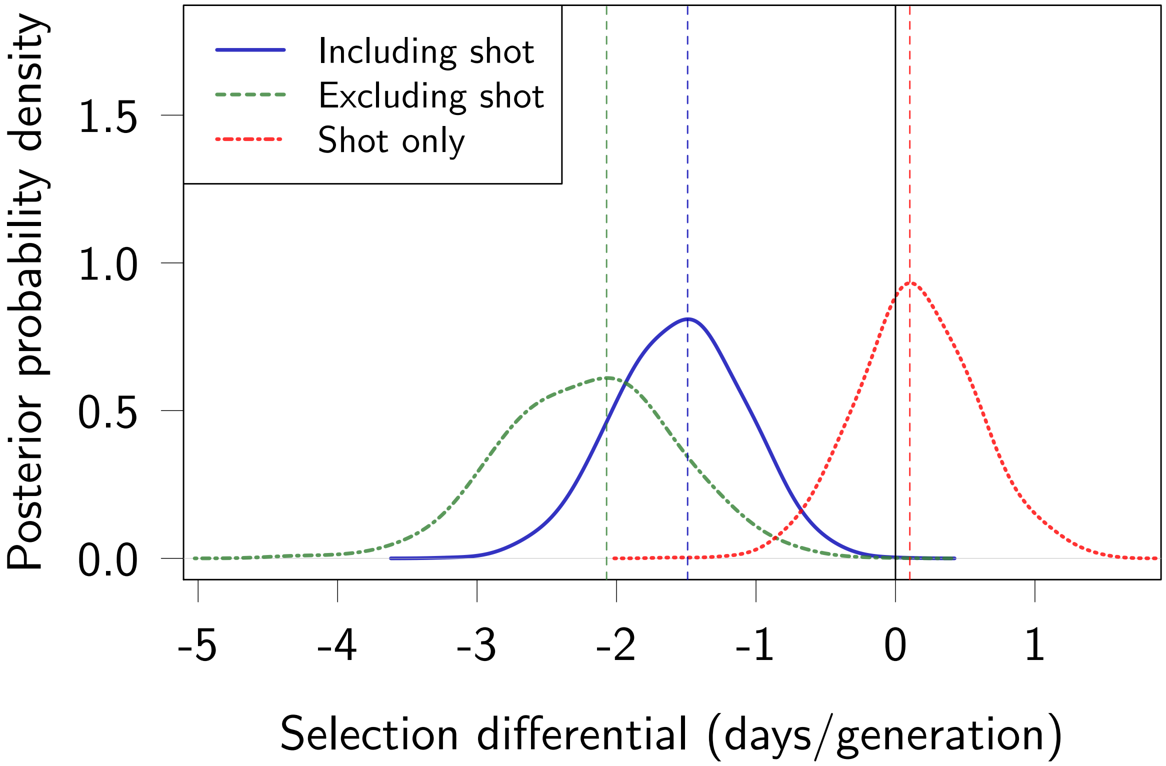

7 Synthesis graphics

Final components of change graph

```
setPar()
opts_chunk$set(dev='tikz', dev.args=list(bg="white"))

#install_github("timotheenivalis/ViolineHills/ViolineHills")
source("violinegraph.R") # frozen version of package

distributions <- list(Demography = list(magelm, mmatlm),
                      Plasticity = list(tempeff, sexchange),
                      Gstructure = list(ggchange, inbchange),
                      Evolution = list(daychanges, daychangesanti, daychangescovA),
                      Unexplained = list(explainedchange, unexplainedchange))
Dcolors <- list(
dem=c(rgb(0.041,0.271,0.537,0.8), rgb(0.341,0.671,0.937,0.4), rgb(0.1,0.5,0, 0.8)),
plas=c(rgb(1,0,0, 0.8),rgb(0.91,0.431,0.437,0.6)),
```

```r
gstr = c(rgb(0.4,0.4,0,0.6),  rgb(0.8,0.8,0,0.4)),
evol= c(rgb(0.814,0.41,0.74, 0.5),rgb(0.314,0.31,0.584, 0.8),rgb(0.514,0.51,0.984, 0.5)),
unex=c(rgb(0.141,0.871,0.337,0.8),rgb(0.141,0.271,0.237,0.4)))

sublegend = list('\\textbf{Demography}' = list(c("Mother age", "Mother reproductive status")),
         '\\textbf{Plasticity}' = list("Temperature", "Offspring sex"),
        '\\textbf{Genetic structure}' = list(c("Gene flow", "Pedigree inbreeding")),
         '\\textbf{Estimates of evolution}'= list(c("BLUPs year", "BLUPs no-year", "STS")),
        '\\textbf{Total (using BLUPs year)}' = list("Explained", "Unexplained"))

par(xpd=FALSE)
ftrs <- function(x){ifelse(x>1.7,1.7,x)}
plotdensities(distributions = distributions,
          sublegend=sublegend, transform = ftrs, btylegend = "n",
          globalmaxdensity = TRUE, violine = FALSE,
          xshift = c(3.5,-3), yshift = c(0.15,0),cexsubl = 0.7,
          legendncol=1, xlab = "Contribution to change (days)", adjust = 1.5,
          col = Dcolors, minden= 0.33, ylab="Probability density",
          main="Components of change", cex.lab=1.1, cex.axis=1.1)

arrows(x0 = -12.3, y0=-2.25, y1=-2.05, xpd = TRUE, col="black", length = 0.15, lwd=5)
```

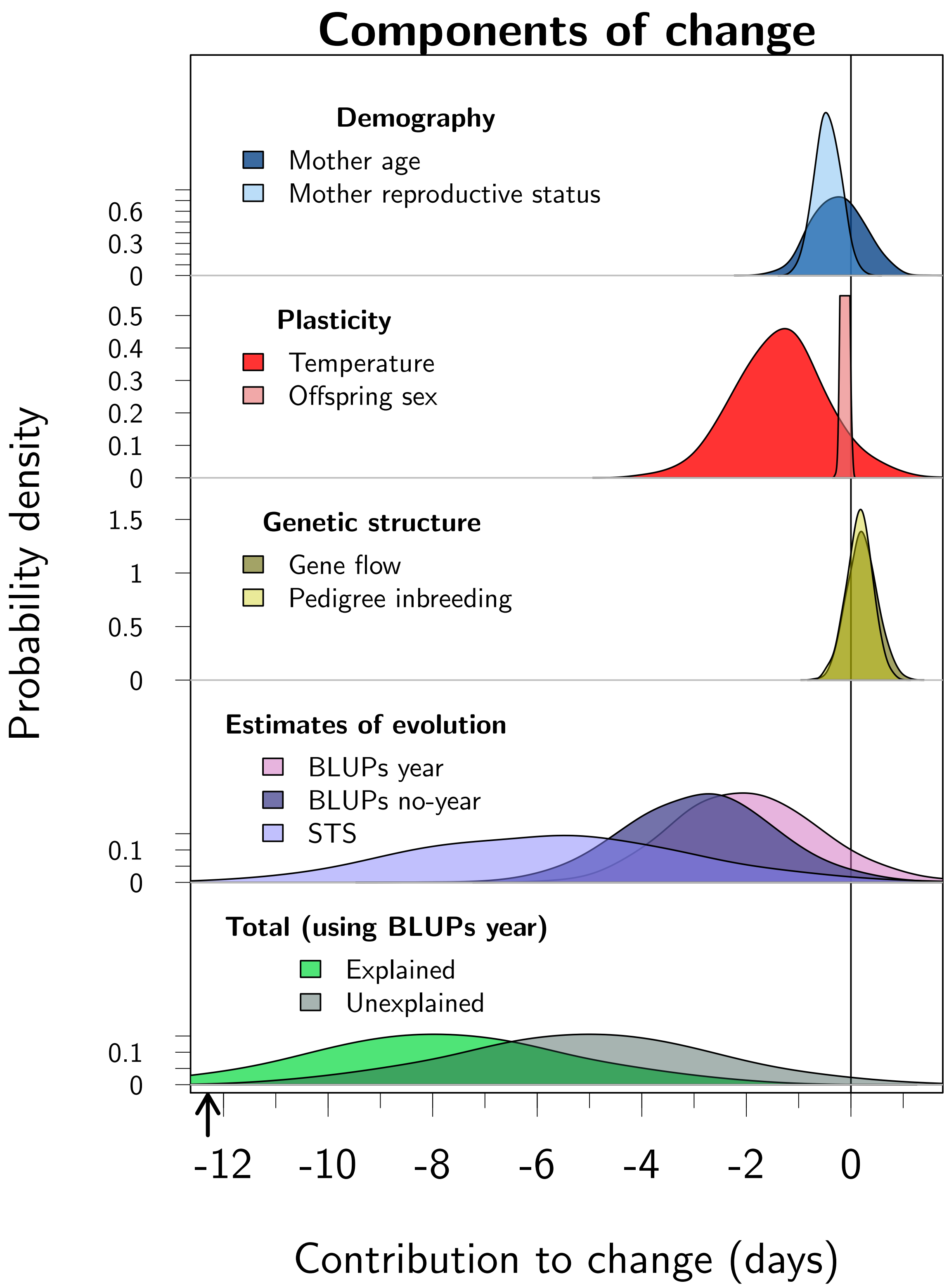

Final selection and evolution graph

```
setPar()
opts_chunk$set(dev='tikz', dev.args=list(bg="white"))

#install_github("timotheenivalis/ViolineHills/ViolineHills")
source("violinegraph.R")

distributions <- list(
```

```
    Drift = list(funtrans(lmBVdrift*(max(birthdata$MumYOB)-min(birthdata$MumYOB)))),
    Predictions = list(funtrans(univarMVr*(max(birthdata$MumYOB)-min(birthdata$MumYOB))),
    funtrans(RmvBD_lbs*(max(birthdata$MumYOB)-min(birthdata$MumYOB)))),
    Estimates = list(daychanges, daychangesanti, daychangescovA))

Dcolors <-list(
  drift=c(rgb(0.141,0.471,0.337,0.4)),
  sel=c(rgb(1,0.694,0.275, 0.7), rgb(0.753,0.435,0, 0.7)),
  evol= c(rgb(0.814,0.41,0.74, 0.5),rgb(0.314,0.31,0.584, 0.8),rgb(0.514,0.51,0.984, 0.5)))

sublegend = list('\\textbf{Drift}' = list("Drift simulations"),
   '\\textbf{Predicted response}' = list(c("Univariate BE", "Multivariate BE")),
   '\\textbf{Estimates of evolution}'= list(c("BLUPs year", "BLUPs no-year", "STS")))

distrilayout <- c(1,2,2,3,3,3)
colgroups <-  1:length(distrilayout)#c(1,2,3,4,4,4)

par(xpd=FALSE)
plotdensities(distributions = distributions,
              sublegend = sublegend,
              sublegside = "left", cexsubl=0.7, btylegend = "n",
              globalmaxdensity = TRUE, violine = FALSE,
              legendncol=2, xlab = "Evolution (days)", adjust = 1.5,
              col = Dcolors, xshift = c(3,-3), yshift = c(0.08,0),
              main="Estimated and predicted evolution",
              ylab="Probability density", cex.lab=1.1, cex.axis=1.1)
```

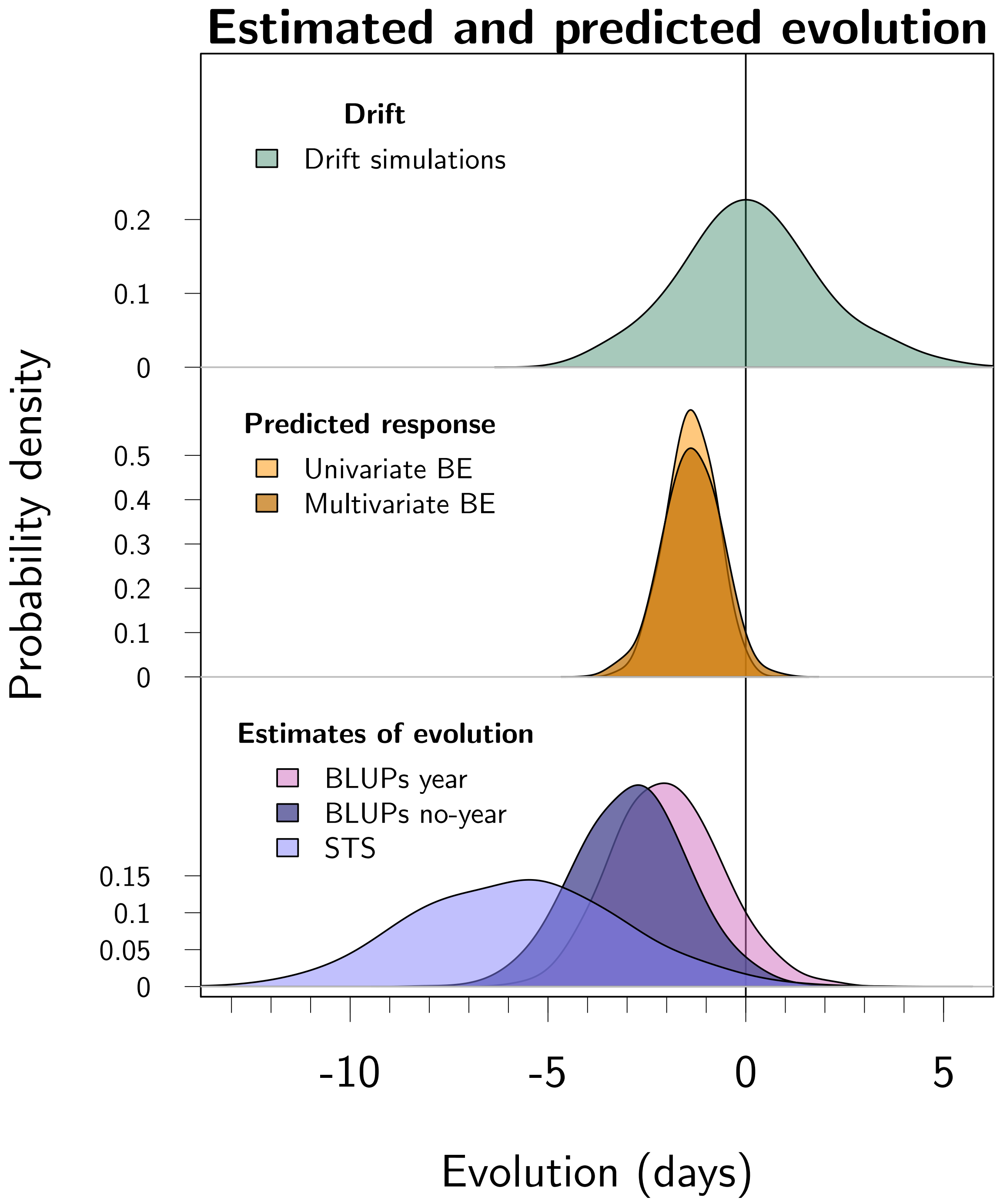

Estimated and predicted evolution
Drift
Drift simulations
Predicted response
Univariate BE
Multivariate BE
Estimates of evolution
BLUPs year
BLUPs no-year
STS
Probability density
0.2
0.1
0
0.5
0.4
0.3
0.2
0.1
0
0.15
0.1
0.05
0
-10
-5
0
5
Evolution (days)
